# Modular Vinyl Phosphonamidates for Cysteine-Directed Protein Targeting

**DOI:** 10.1021/jacs.5c22349

**Published:** 2026-02-10

**Authors:** Christian E. Stieger, Charlotte Völkel, Mathias B. Bertelsen, Michael Lisurek, Jan Vincent V. Arafiles, Jonathan Franke, Leander Crocker, Karl Schuppe, Yunjae Lim, Christiane Groneberg, Mathias Christmann, Han Sun, Christian P. R. Hackenberger

**Affiliations:** † Leibniz-Forschungsinstitut für Molekulare Pharmakologie (FMP), Robert-Rössle-Straße 10, 13125 Berlin, Germany; ‡ Department of Chemistry, Humboldt Universität zu Berlin, Brook-Taylor-Straße 2, 12489 Berlin, Germany; § Institute of Chemistry and Biochemistry, Freie Universität Berlin, Takustraße 3, 14195 Berlin, Germany; ∥ Institute of Chemistry, Technische Universität Berlin, Straße des 17. Juni 135, Berlin 10623, Germany

## Abstract

Covalent inhibitors and chemical probes targeting ligandable cysteine residues have emerged as powerful tools for drug discovery and proteomics. In this study, we introduce vinyl phosphonamidates (VPAs) as a novel class of latent cysteine electrophiles and assess their reactivity, selectivity, and potential for developing covalent inhibitors. Compared to well-established chloroacetamide and acrylamide electrophiles, VPAs exhibit a significantly lower intrinsic reactivity toward the model thiol glutathione. Moreover, VPA-derived covalent fragments displayed only very limited nonspecific reactivity in human cell lysate. Encouraged by these results, we developed VPA-functionalized derivatives of the FDA-approved covalent inhibitors Afatinib and Ibrutinib and evaluated their ability to engage the target protein by gel-based and mass spectrometry-based activity-based protein profiling (ABPP). Compared to commonly employed Michael acceptor-based electrophilic groups, VPA-functionalized drug ligands displayed significantly less off-targets while maintaining inhibitor efficiency. Furthermore, we leveraged the modular nature and accessibility of VPAs to develop a bifunctional proteolysis targeting chimera (PROTAC) for targeted protein degradation. The demonstrated selectivity and modularity, as exemplified by the incorporation of various ligands on the phosphorus *O*-substituent, of the vinyl phosphonamidate group as a cysteine-directed electrophile highlight its ability to expand the chemical space in the development of covalent inhibitors with a favorable proteome-wide reactivity profile.

## Graphical abstract

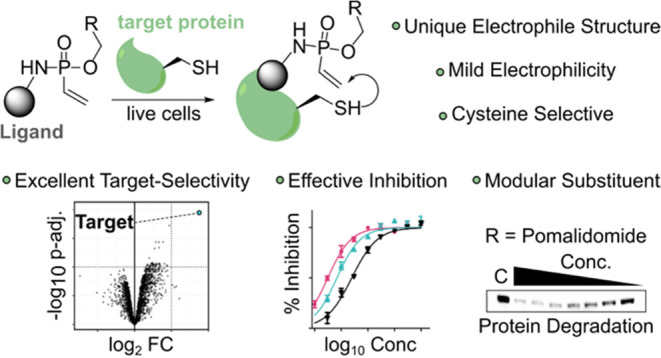

## Introduction

In recent years, the use of electrophilic groups that target nucleophilic side chains of proteins has become increasingly important in chemical biology and drug discovery.^1–3^ While highly reactive, residue-specific reagents are desirable in protein bioconjugation^2,4^ and chemoproteomics,^5^ mild electrophiles find application in targeted covalent inhibition (TCI) and protein-selective probe development.^6–9^ Owing to its high nucleophilicity under physiological conditions and its relatively low natural abundance, cysteine is the primary target for covalent probes and inhibitors.^10^

The covalent, often irreversible, mode of action introduced by the addition of electrophiles to existing ligands can lead to enhanced and sustained drug efficacy,^11^ the overcoming of acquired drug resistance,^12^ and even isoform selectivity compared to the noncovalent counterpart.^13^ However, their potential off-target reactivity has raised concerns about latent toxicity.^14,15^ While historic covalent inhibitors like Aspirin or penicillin were discovered serendipitously, more recent examples of TCI follow the ligand-first principle, where a highly optimized drug scaffold is further functionalized with an electrophilic group to target a nearby amino acid side chain.^1,6,10^ Thereby, considerable progress has been made in developing covalent inhibitors for several cancer-associated protein kinases like Bruton’s tyrosine kinase^16^ or epidermal growth factor receptor (EGFR).^17^ More recently, emerging technologies made it possible to perform TCI discovery from an electrophile-first perspective. In this case, covalent ligands against protein targets of interest are identified before structure-based optimization.^6^ This route has led to the development of a variety of covalent compounds currently investigated in the clinics, as well as to FDA-approved drugs against the KRAS GTPase with the cancer-associated G12C mutation.^18,19^ For both approaches, the electrophilic reactive group needs to be fine-tuned to enable fast and efficient proximity-induced on-target reactivity while minimizing unwanted reactions with off-targets and depletion by ubiquitous thiol nucleophiles like glutathione. While substituted and unsubstituted acrylamides remain the most prominently used electrophiles, new types of warheads are also becoming increasingly popular.^1,20,21^ Recent examples for cysteine-directed reactive groups used in the development of TCIs include strain-release-type electrophiles,^22,23^ next-generation S_N_2-type electrophiles,^24–26^ S_N_Ar-type electrophiles,^27–29^ and other electrophilic heterocycles^30–32^ (Figure 1a–d).

**Figure 1:**
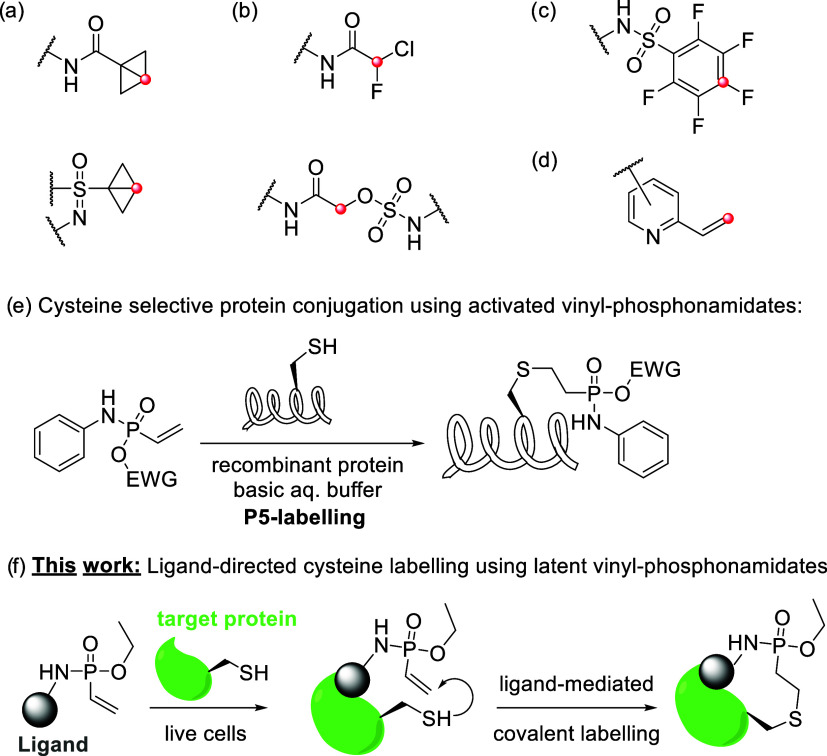
(a–d) Structure of cysteine-targeting electrophiles employed in TCI. (e) Schematic representation of cysteine-selective P5-labeling of proteins using activated vinyl phosphonamidates. (f) Schematic representation of the ligand-directed and cysteine-selective protein labeling using latent vinyl phosphonamidate electrophiles.

Previously, our group has introduced unsaturated P­(V) compounds for cysteine-selective protein conjugation (P5-labeling, Figure 1e).^33,34^ In these studies, we observed that depending on the substitution pattern of the phosphorus center, the electrophilicity of the β-carbon can be significantly altered.^33,35^ While previous applications were mainly focused on the generation of highly reactive reagents for protein or antibody bioconjugation^34^ or proteome-wide cysteine profiling,^36^ in this manuscript, we wanted to explore the versatility of the less reactive P­(V) electrophiles in ligand-directed cysteine labeling (Figure 1f). Compared to other electrophiles employed in TCI, thanks to the modular composition of the P­(V) center, vinyl phosphonamidates (VPAs) come with several add-ons. First, the tetrahedral geometry offers a unique, currently underexplored structural feature for cysteine-directed targeting. Moreover, the additional O-substituent allows for additional structural and functional alterations as well as reactivity fine-tuning.^33,37^

## Results and Discussion

### General Reactivity of VPAs

At the onset of our studies, the general electrophilicity of VPA reagents **1** was compared to the well-established acrylamide **2** and chloroacetamide **3** electrophiles. For this aim, we tested the reactivity of model compounds **1a**–**3a** toward reduced glutathione (GSH) as a model thiol at physiological pH (7.4) (Figure 2a). While *N*-phenyl-substituted chloroacetamide **3a** was almost fully consumed after 3 h of reaction (resulting in a *t*_1/2_ of 0.8 h, Figure 2b), analogous acrylamide **2a** showed a more tempered reactivity, yielding a *t*_1/2_ of 2.6 h. The values obtained are in good agreement with similar experiments that were previously conducted by others.^22,24^ In contrast to these more classic cysteine electrophiles, *N*-phenyl-*O*-ethyl-substituted VPA **1a** showed barely any consumption over the course of 3 h (*t*_1/2_ = 235 h, Figure 2b). Moreover, we previously demonstrated that **1a** reacts only very slowly with glutathione, even at higher concentrations and elevated pH, underscoring its low intrinsic reactivity.^33^ In addition to assessing the cysteine reactivity, we also examined the reactivity of **1a** toward l-lysine. To this end, unprotected lysine (10 equiv) was incubated with **1a** for 5 days (pH 7.4 and pH 8.5, room temperature). Throughout the entire incubation period, no reaction or degradation products were detected, highlighting both the cysteine selectivity and buffer stability of vinyl phosphonamidates (Figure S16).

**Figure 2:**
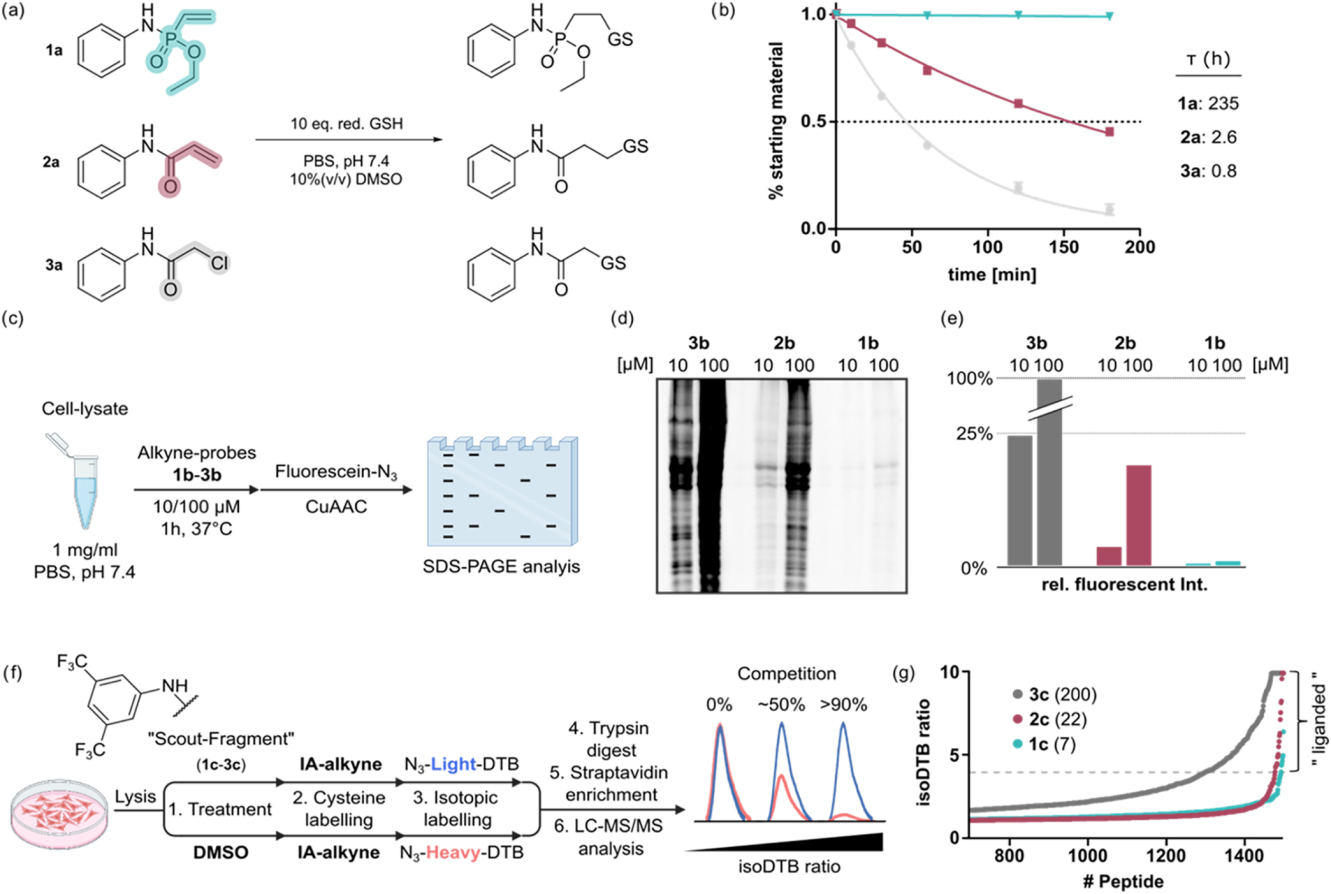
Cysteine reactivity of weak thiol electrophiles. (a) Schematic representation of the reactivity of model compounds **1a**–**3a** toward reduced glutathione (GSH, 10 equiv) in PBS (pH 7.4, 10% DMSO) at room temperature. (b) Time-dependent decay of **1a**–**3a** in the presence of GSH. The half-life was determined by fitting the consumption of the starting material to a one-phase decay. (c) Cysteine reactivity of alkyne-functionalized probes **1b**–**3b** in RAMOS cell lysate (1 mg/mL, PBS pH 7.4, 10/100 μM compound, 1 h 37 °C). (d) In-gel fluorescence analysis of global cysteine reactivity. (e) Relative densitometry analysis of the fluorescent gel depicted in (d). (f) Schematic representation of the employed isoDTB workflow. (g) Waterfall plot representing the top 750 cysteine-containing peptides and their corresponding competition ratio (isoDTB ratio = DMSO/compound). Greater reactivity yields a higher isoDTB ratio. Ratios ≥ 4 (dashed line) are considered liganded. Number of liganded cysteines for **1c**–**3c** indicated in brackets.

Nonetheless, since the formation of the glutathione conjugate was clearly notable, we wanted to investigate the reactivity of **1a** with a more diverse set of thiols. Therefore, we examined all three electrophilic groups for their ability to label cysteines in whole-cell lysates. First, alkyne-functionalized reagents (**1b**–**3b**, for structures, see Scheme S1) were synthesized to allow for gel-based activity-based protein profiling (ABPP). Next, RAMOS cell lysate (1 mg/mL, PBS pH 7.4) was incubated with 10 and 100 μM of the corresponding compounds for 1 h at room temperature, followed by labeling with fluorescein azide via CuAAC and subsequent analysis by SDS-PAGE (Figure 2c). Since all compounds share the same scaffold and differ only by their reactive functional group, we anticipate that any differences in labeling intensity should primarily stem from differences in electrophilicity. For all compounds, a concentration-dependent labeling could be observed, with **3b** yielding the most intense fluorescent signal. In accordance with the glutathione reactivity, acrylamide probe **2b** was the second most reactive reagent, reaching a labeling intensity of approximately 20%. PA probe **1b**, however, only produced a very faint signal (<1%, Figures 2d,e and S1). The same experiment was also performed using live RAMOS cells, and a similar trend was observed (Figure S2). To rule out that VPA compounds show impaired cell permeability, as it was observed for phosphine oxides,^38^ we performed an adopted chloroalkane permeability assay (CAPA, Figure S3a).^39,40^ To this end, we synthesized a chloroalkane-functionalized acrylamide as well as the corresponding VPA (**S10** and **S12**, Figure S3b). CAPA reporter cells were prepared by transiently transfecting HEK293T with a plasmid enabling the cytoplasmic expression of the Halotag protein, and concentration-dependent cell permeability was assessed. While both compounds showed excellent cell permeability at 10 μM, resulting in full labeling of the HaloTag reporter (Figure S3c), the CP_50_ for VPA **S12** was approximately 10× lower than the acrylamide version **S10** (Figure S3d). We therefore conclude that conjugation of VPA to small molecules is unlikely to compromise the cell permeability.

This overall low reactivity can be seen as an advantage, since it suggests that the reactive group itself is unlikely to favor unspecific cysteine reactivity. However, PAs might still be reactive enough to label cysteine residues in a ligand-directed manner. To investigate this hypothesis, we adopted a quantitative cysteine chemoproteomics workflow as introduced by Backus et al. (Figure 1f).^41^ Therefore, electrophilic “scout fragments” (**1c**–**3c**, for structures, see Scheme S1) containing the corresponding reactive group were synthesized and employed in competitive quantitative cysteine proteomics. For this purpose, RAMOS cell lysate was either treated with 100 μM of the corresponding fragment or with DMSO as the nontreated control, and samples were further processed according to Zanon et al.^42^ In accordance with the previous experiments, phosphonamidate-modified scout-fragment **1c** liganded the lowest amount of cysteines, while especially chloroacetamide **3c** showed very high promiscuity. Due to the low intrinsic reactivity of the phosphonamidate electrophile, we reasoned that the functionalization of a more elaborate ligand would facilitate the efficient targeting of cysteines within biological systems.

### Inhibition and In Cellulo Labeling of EGFR Using VPA-Based Inhibitors

To continue with this endeavor, we chose the first approved TCI-based pharmaceutical as a scaffold, namely, the second-generation EGFR-targeting drug Afatinib.^43^ Afatinib and the third-generation derivative Osimertinib^44^ are powerful modalities to treat non-small-cell lung cancer, designed to irreversibly inhibit EGFR kinase activity by forming a covalent bond with a noncatalytic cysteine (Cys797) in the ATP-binding pocket. These TCIs are particularly effective in overcoming acquired resistance to conventional reversible inhibitors, such as Erlotinib and Gefitinib, in lung cancer patients with the T790 M resistance mutation in EGFR.^45,46^ We proposed replacing the original 4-dimethylaminocrotonamide (DMAC) warhead with a VPA in **4a** (Figure 3a) to probe the use of VPAs in engineering TCIs. In addition, since the anticipated vinyl phosphonamidate-quinazoline contains a chiral *P-*center, we also planned to investigate whether one of the two stereoisomers (here, diastereomers because of the chiral furanyl substituent) would be favored. Covalent docking on the publicly available crystal structure of EGFR-bound Afatinib indicated that both stereoisomers of **4a** should be able to form a covalent bond with Cys797 of EGFR (PDB: 4G5J, Figure S4).^47^ Moreover, the molecular docking suggested that the PA-functionalized ligand interacts with the target protein in a similar manner as the original drug (Figure 3b).

**Figure 3:**
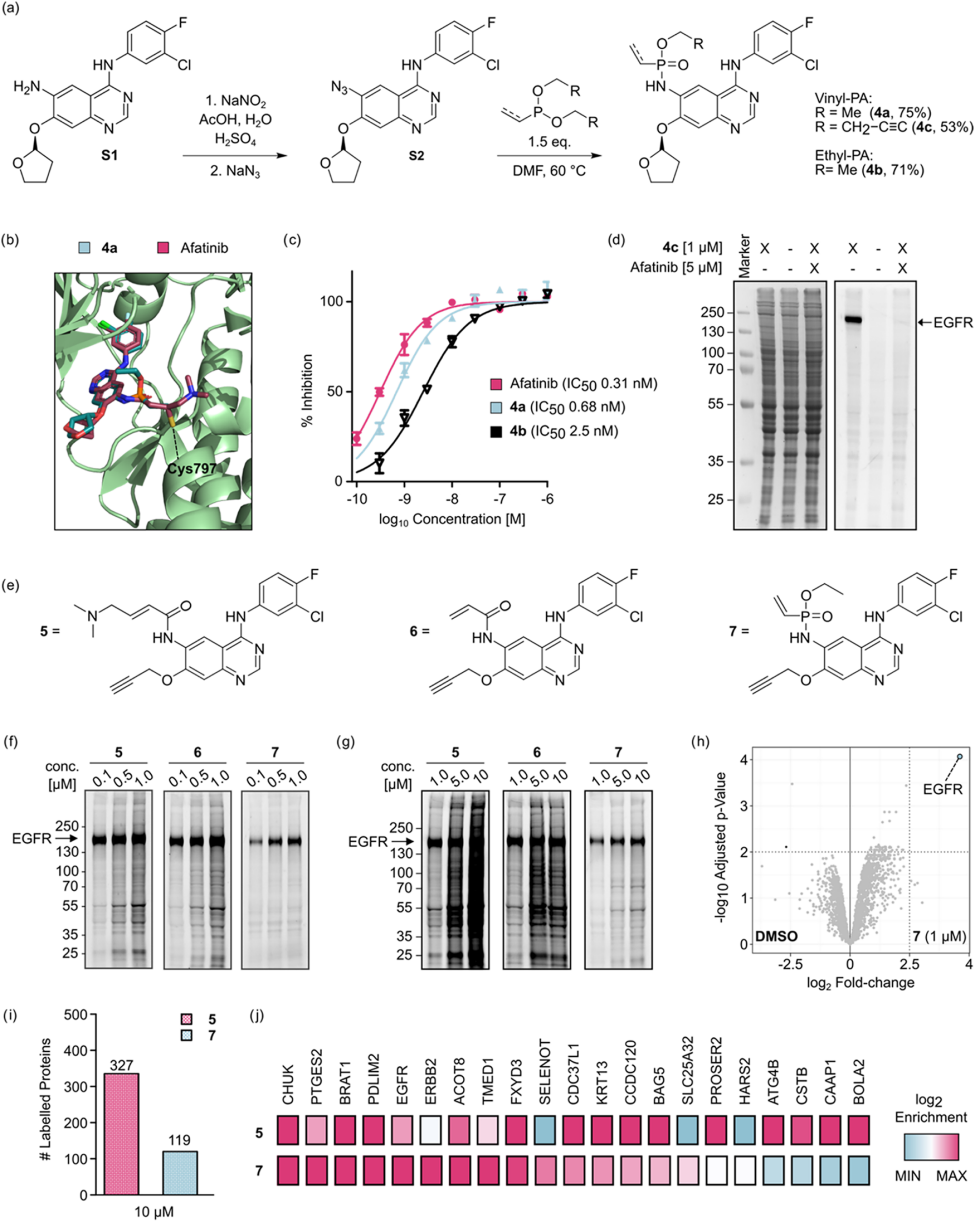
(a) Synthetic route toward PA-functionalized Afatinib analogues **4a**–**4c**. (b) Superposition of Afatinib and the phosphonamidate analogue **4a** covalently docked onto the crystal structure of wt EGFR (PDB: 4G5J). (c) In vitro kinase inhibition assay performed with the corresponding covalent inhibitors (**4a** and Afatinib) and noncovalent inhibitor (**4b**). (d) Competitive labeling of probe **4c** and Afatinib in A431 cells. (e) Chemical structure of the employed EGFR-targeting alkyne probes **5**–**7**. (f, g) Concentration-dependent labeling of probes **5–7** in A431 cells. (h) Label-free quantification of the pulldown performed with 1 μM **7**. Proteins with a log_2_-fold change >2.5 compared to the DMSO control and an adjusted *p*-value <0.01 were considered to be significantly enriched. (i) Total number of significantly enriched proteins obtained from the pulldown with 10 μM probes **5** and **7**. (j) Heatmap comparison of the enrichment efficiency for selected proteins obtained from the pulldown with 10 μM probes **5** and **7**.

Compound **4a** was synthetically accessed by transforming the Afatinib precursor **S1** into the corresponding aryl azide (**S2**), followed by the chemoselective Staudinger phosphonite reaction (Figure 3a).^33^ Following this route, the desired drug analogue **4a** was obtained in good yield (75%). With this molecule in hand, we wanted to investigate the ability of the PA-equipped ligand to inhibit the kinase activity of EGFR. To our delight, **4a** inhibited wild-type EGFR in an in vitro kinase assay with an IC_50_ of 0.67 nM. This inhibitory effect was significantly stronger than that of the nonreactive ethyl phosphonamidate analogue **4b** (IC_50_ = 2.5 nM, Figure 3c), and it was in a comparable range to Afatinib (0.31 nM). These results indicate that **4a** engages EGFR in a covalent manner similar to the original drug. In addition, we also determined the kinetic parameters governing the covalent inhibition of **4a** (Figure S5). Using a previously used and commercially available sensor-peptide-based assay^48^ with varying concentrations of **4a** (2–50 nM), we obtained a *k*_inact_ of 0.24 × 10^–3^ s^–1^ and a *K*_I_ of 2.43 nM, which is in a comparable range to previously reported values for Afatinib (*k*_inact_ = 0.9 × 10^–3^ s^–1^ and a *K*_I_ = 0.15 nM).^49^ In direct comparison, we obtained a *k*_inact_/*K*_I_ ratio of 164 × 10^3^ M^–1^ s^–1^ for Afatinib, which is approximately two times faster than **4a** (97 × 10^3^ M^–1^ s^–1^).

To assess whether the PA analogue, which is also able to engage EGFR in cellulo, we synthesized an alkyne-functionalized variant and probed it in gel-based ABPP. Usually, more sophisticated early-stage chemical optimization is required to obtain an alkyne-functionalized probe with unperturbed target-binding abilities. However, the chemoselective Staudinger phosphonite reaction allowed us to easily incorporate an alkyne-containing substituent into target inhibitor **4c** in 53% yield (Figure 3a). With this probe in hand, we treated wild-type EGFR overexpressing A431 cells at a probe concentration of 1 μM for 1 h at 37 °C, followed by lysis and fluorescent labeling via CuAAC. In-gel fluorescence analysis showed an intense signal between 130 and 250 kDa, indicative of EGFR, in the probe-treated sample, whereas no distinct signal was visible in the DMSO control (Figure 3d). Moreover, the intense fluorescent signal disappeared after pretreatment with excess Afatinib (5 μM, 1 h, 37 °C), indicating selective covalent engagement of EGFR.

For a fair comparison to the previously validated Afatinib-derived probes **5** and **6**,^50^ we synthesized the corresponding phosphonamidate analogue **7** (Scheme S2 and Figure 3e). In this case, the probe molecule solely differs in the nature of the electrophilic group. Similar to **4c**, this probe showed EGFR-selective labeling in A431 cells that was abolished by pretreatment with excess Afatinib (Figure S6). Moreover, a concentration-dependent labeling experiment revealed a detectable signal at concentrations as low as 5 nM (Figure S7). When comparing probes **5**–**7**, an intense band could be detected around 150 kDa (indicative of EGFR) for all probes and concentrations tested (Figure 3,g). In contrast to the Michael acceptor-based probes, VPA probe **7** showed outstanding target selectivity, even at probe concentrations >1 μM. Densitometric analysis of the in-gel fluorescence revealed an almost 10-fold improvement compared to DMAC-containing probe **5** and a more than 3-fold increased selectivity compared to probe **6** at 10 μM (Figure 3g). In addition to the concentration-dependent labeling, we performed a time-course labeling experiment using **5** and **7**. While especially at shorter reaction times (30–120 min), both probes display excellent selectivity for the target protein, prolonged incubation times resulted in a significantly higher off-target labeling for **5** (Figure S8). It should be stated that the target selectivity of **7** did not decrease over 6 h of incubation. Even in an EGFR-negative cell line (RAMOS), the comparably high reactivity of the DMAC electrophile resulted in significant fluorescent labeling of off-targets, especially at elevated concentrations (5 and 10 μM). Moreover, even at a lower concentration of 1 μM, a pronounced signal could be observed with increased incubation time. In contrast, the treatment with phosphonamidate **7** resulted in almost no labeling under the tested conditions, further highlighting the excellent selectivity of the developed probe. (Figure S9).

To gain a more detailed understanding of which proteins are labeled by the corresponding probe, we performed a proteomic pulldown experiment. Following a 2 h incubation at 37 °C (1 μM compound, 1% DMSO), cells were washed, lysed, and clicked to biotin-N_3_, followed by sample preparation according to the SP2E workflow introduced by Kielkowski and co-workers (see SI 4.6 for a detailed procedure).^51^ Label-free quantification (LFQ) of the proteomic analysis revealed that EGFR is the only protein significantly enriched for **7** compared to the DMSO-treated control (Figures 3h and S10). This result once more highlights the excellent selectivity of the PA electrophile. Additionally, we also wanted to investigate proteomic engagement under more extreme conditions, using a 10 μM probe concentration. In this case, both **5** and **7** enriched a significantly larger portion of proteins (118 and 327 off-targets, respectively; Figure 3i). Even though both probes enriched a large portion of proteins, EGFR was still among the most prominent hits for the phosphonamidate probe and only to a lesser extent for **5** (Figure S11). Overall, we observed a certain degree of complementarity between the off-targets obtained from the corresponding probes (Figures 3j and S12). While some proteins were equally highly enriched among both probes (e.g., CHUK, BRAT1, and PDLIM2), others showed a certain preference for one of the electrophilic groups. Most prominently, selenoprotein SELENOT and solute carrier protein SLC25A32 were only enriched by phosphonamidate probe **7**. On the other hand, several proteins like the cysteine proteases ATG4B, CTSB, and BOLA2 were exclusively enriched by DMAC probe **5**. Taken together, these results underline that VPAs are excellent electrophiles for the development of selective chemical probes, cysteine-targeting covalent inhibitors.

### Labeling and Inhibition of BTK Using VPA-Based Probes

Motivated by these promising results, we wanted to extend the scope toward a different class of kinase inhibitors. Ibrutinib is an FDA-approved Bruton’s tyrosin kinase (BTK) inhibitor that is commonly used for the treatment of B-cell malignancies.^16^ In contrast to Afatinib, Ibrutinib does not contain an aromatic but an aliphatic acrylamide as the electrophilic group. Since aliphatic VPAs were found to be less reactive and prone to hydrolysis under acidic conditions,^33^ we had to undertake some structural alterations. Previous reports demonstrated that the Ibrutinib scaffold can tolerate structural modifications in the region of the electrophilic warhead.^22^ Inspired by these findings, we synthesized a series of VPA probes based on the pyrazolo­[3,4-*d*]­pyrimidine scaffold (**8**–**11**, Figure 4a), each harboring a different aromatic linker. To compare their reactivity and selectivity for BTK, we performed a gel-based ABPP experiment using RAMOS cells. The well-established BTK probe PF-06658607 (**12**) showed a prominent band for BTK around 80 kDa with an additional prominent off-target band around 60 kDa (Figure 4b). In contrast, for all four PA probes, only one intense band was obtained at the same height as BTK. Among those, probe **9** yielded the most intense labeling, comparable to the signal of acrylamide probe **12**. Performing the same experiment in RAMOS cell lysate resulted in a similar outcome, with an even more pronounced labeling for **9** (Figure S13). Analogously to Afatinib-derived probe **4a**, we wanted to investigate whether one stereoisomer might show preferential binding. Covalent docking onto the crystal structure of Ibrutinib-bound BTK suggests that both isomers of **9a** (Scheme S3) are positioned in the ATP-binding pocket in a configuration similar to that of Ibrutinib. Moreover, since the *O*-substituent of VPA points outward of the binding pocket, we do not expect any significant alterations of the binding affinity for one of the isomers, even with bulkier substituents (Figure 4c).

**Figure 4:**
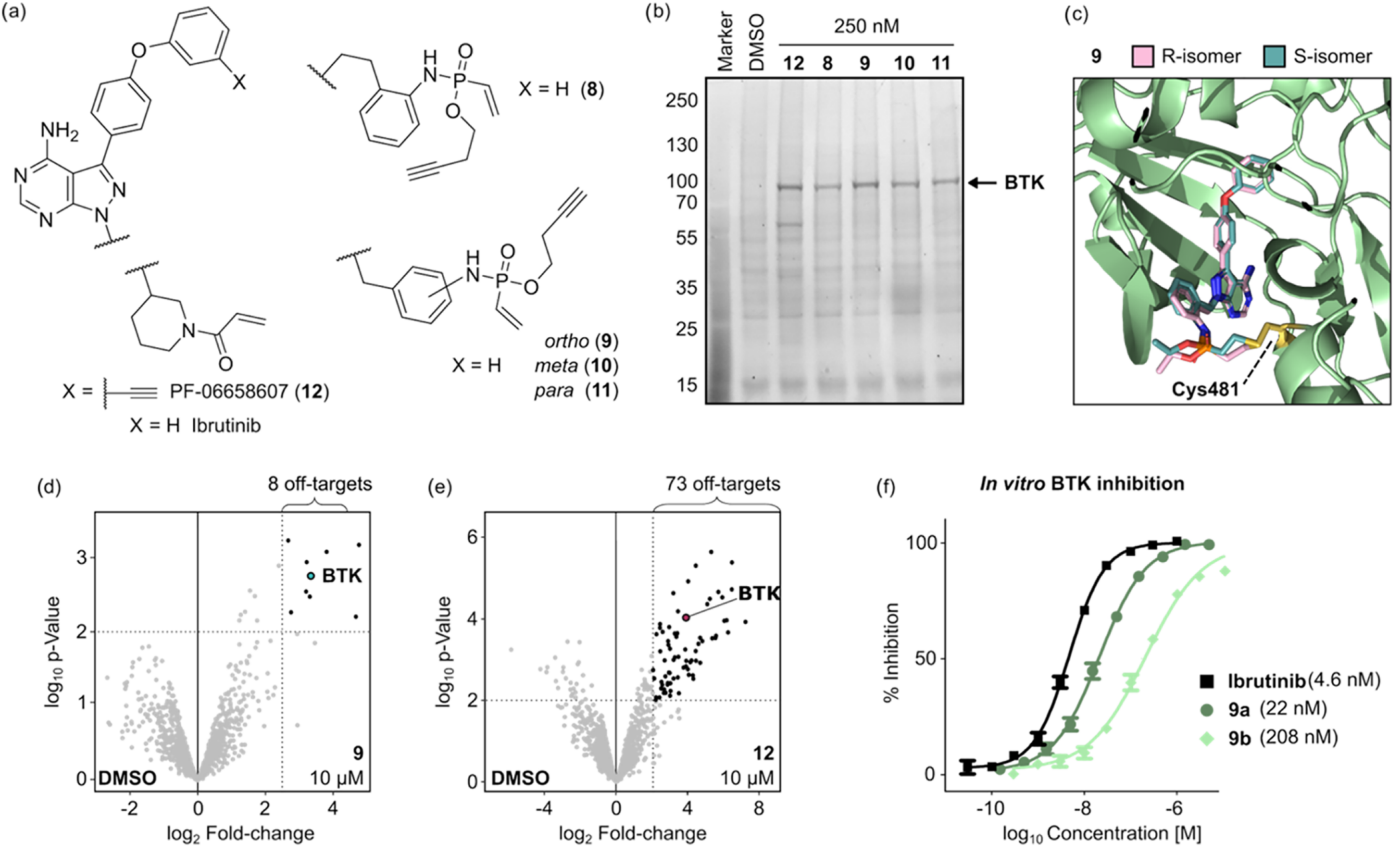
(a) Chemical structures of Ibrutinib and corresponding reactive probes (**8**–**11**) employed in BTK targeting. (b) Comparison of the in-cell reactivity profiles of BTK probes **8**–**12** in RAMOS cells (250 nM, 1 h, 37 °C) analyzed by gel-based ABPP. (c) Superposition of the two stereoisomers of **9** covalently docked onto the crystal structure of BTK (PDB: 5P9J). (d, e) Label-free quantification of proteins significantly enriched in the pull-down with 10 μM of either probe **9** (d) or **12** (e). Proteins with a log_2_-fold change >2.5 compared to the DMSO control and a *p*-value <0.01 were considered to be significantly enriched. (f) In vitro kinase inhibition assay performed with the corresponding covalent inhibitors (**9a** and Ibrutinib) and a noncovalent inhibitor (**9b**).

To verify the identity of the protein(s) labeled by our best-performing probe **9**, we performed in-cell labeling followed by streptavidin pull-down coupled to LC-MS/MS analysis. Since our previous data obtained from EGFR probes **5** and **7** indicated that at low concentration differences in the proteome-wide off-targets are less apparent, we performed the experiment with 10 μM of the corresponding probe (Figure 4d,e). While VPA **9** managed to enrich BTK in a highly selective manner with only three off-target proteins, acrylamide-based probe **12** was by far more promiscuous (73 off-targets).

Finally, the inhibitory efficiency of the developed vinyl phosphonamidate-based Ibrutinib analogue was investigated (Figure 4f). To this end, we synthesized the *O*-ethyl-substituted counterpart of BTK probe **9** (**9a**, Scheme S3). The in vitro kinase inhibition assay demonstrated that VPA **9a** significantly inhibited BTK kinase activity with an IC_50_ in the lower nanomolar range (22 nM). Even though Ibrutinib was almost five times more potent than **9a**, this inhibition efficiency is comparable to Ibrutinib analogues equipped with a bicyclobutane carboxylic amide^22^ or a 3-bromo-4,5-dihydroisoxazole^31^ electrophile (both 17 nM). Additionally, **9a** was markedly more potent than nonreactive ethyl phosphonamidate **9b** (Scheme S3), which had an IC_50_ of only 208 nM. Taken together, these results strongly suggest that VPA **9a** effectively inhibits the kinase activity of BTK through covalent interactions.

### Leveraging the Modularity of VPAs for Single-Step Labeling and PROTAC Development

Based on our docking studies, which indicate that the *O*-substituent points out of the binding pocket, we sought to exploit this structural feature to our advantage. Many protein-specific covalent probes rely on an alkyne tag as a minimal biorthogonal recognition handle for downstream applications. These minimal tags are usually introduced in a way that perturbs the binding of the ligand as little as possible. However, prefunctionalized probes might be advantageous, as they can facilitate high-throughput screening and imaging applications.^21,52^ To test if large substituents such as fluorophores can be introduced without interfering with the covalent labeling of BTK, we synthetically functionalized our probe **9** with fluorescein azide (**13**, 82% yield, Figure 5a). Since fluorescein is known to be cell-impermeable, we incubated RAMOS cell lysate with increasing concentrations of **13** and analyzed it via gel-based ABPP (Figures 5b and S15). Even at low nanomolar concentrations, a distinct fluorescent band corresponding to the molecular weight of BTK was visible. The labeling intensity increased in a dose-dependent manner with excellent target selectivity, even at higher probe concentrations of 1 μM.

**Figure 5:**
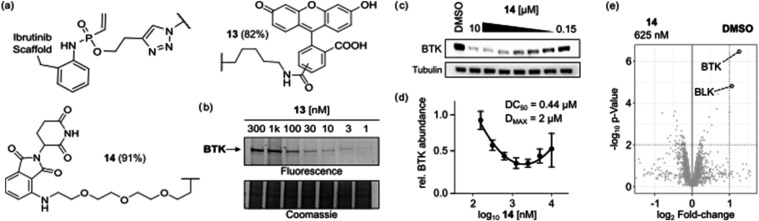
(a) Chemical structures of compounds **13** and **14** obtained via late-stage functionalization of probe **9**. (b) Concentration-dependent in-gel ABPP experiment using prefunctionalized probe **13** in RAMOS cell lysate (1 h, 37 °C). (c) Anti-BTK Western blot of RAMOS cells incubated with the indicated concentration of PROTAC **14** for 24 h at 37 °C. Data is representative of independent triplicates. (d) Quantitative analysis of the relative BTK intensity obtained from independent experiments (*n* = 3). The obtained data was fitted to a second-order polynomial curve to obtain DC_50_ and D_MAX_ (e) Label-free quantification RAMOS cells after treatment with 0.625 μM **14** or DMSO for 24 h at 37 °C. Proteins with a log_2_-fold change >1 and a *p*-value <0.01 compared to the DMSO-treated control were considered to be significantly degraded.

Given the fact that bulkier *O*-substituents are well tolerated by the VPA-functionalized ligand scaffold, we were eager to develop a bifunctional protein degrader. Proteolysis targeting chimera (PROTACs) represent a promising class of therapeutics that act by inducing proximity between the target protein and an E3 ubiquitin ligase, which in turn leads to polyubiquitination and eventually proteasome-mediated degradation of the target.^53^ To build a covalent VPA-based PROTAC, we used the commonly used E3 ligase recruiter pomalidomide targeting CRBN and linked it to **9** via a short PEG spacer (**14**, 91% yield). We then evaluated whether **14** could induce BTK degradation in the RAMOS cells. Western blot analysis indicated a concentration-dependent degradation of BTK after 24 h (Figure 5c). Quantitation of the obtained results revealed a DC_MAX_ of around 77% and a DC_50_ of 0.44 μM (Figure 5d). Finally, we investigated the proteome-wide selectivity of **14** by using label-free quantitative proteomics. Among all quantified proteins, only BTK and the known Ibrutinib off-target BLK were depleted by more than 50% and a *p*-value <0.01 (Figure 5e). These results furthermore highlight the excellent selectivity of the herein presented VPA electrophile.

## Conclusion

In summary, we have introduced VPAs as a promising class of cysteine-targeting electrophiles for the development of covalent chemoproteomic probes and covalent inhibitors. Initial investigations revealed that VPAs exhibit significantly lower intrinsic electrophilicity compared to traditional chloroacetamide and acrylamide electrophiles, highlighting their potential to increase proteome-wide selectivity. By leveraging the straightforward accessibility and modularity of unsaturated P­(V) derivatives, we successfully incorporated the VPA electrophile into targeted covalent inhibitors (TCIs) of EGFR and BTK, which effectively inhibited kinase activity and selectively engaged their respective targets in living cells, as confirmed by gel-based and proteomic profiling. Notably, the PA-functionalized probes exhibited significantly reduced off-target engagement compared with their acrylamide counterparts, underscoring their potential for enhanced target specificity in covalent drug discovery. Furthermore, the modular structure of VPA electrophiles allowed us to accommodate diverse *O*-substituents without significantly compromising the ability to engage the target protein. Moreover, we were able to utilize this unique structural feature to incorporate an alkyne handle, fluorescein, and even the E3 ligase recruiter pomalidomide, furnishing a selective covalent PROTAC. Altogether, our findings establish VPAs as a unique and versatile class of electrophiles for covalent drug design, with broad applications in chemical biology, targeted covalent inhibition, and protein degradation strategies.

## Supplementary Material





## Data Availability

Data generated in this study are provided in the manuscript and Supporting Information. Additional data supporting the findings of this study are available from the corresponding author upon reasonable request. The mass spectrometry proteomics data have been deposited to the ProteomeXchange Consortium via the PRIDE^54^ partner repository with the data set identifier PXD064073.

## Notes

Schemes S1–S3, Figures S1–S15, general information, experimental procedures, synthetic procedures and additional references (PDF)
NMR spectra, uncropped gel images, and Western blots (PDF)
