# Supplementary material for: Modular Vinyl Phosphonamidates for Cysteine-Directed Protein Targeting
Source: J Am Chem Soc. 2026 Feb 10;148(7):7772–81. doi: 10.1021/jacs.5c22349 (PMC12951430; doi:10.1021/jacs.5c22349)
Supplement: Supplementary file 1 [file ja5c22349_si_001.pdf]

Supporting Information

Modular Vinyl-Phosphonamidates for Cysteine Directed Protein Targeting

Christian E. Stieger[1,2*], Charlotte Völkel[1,3], Mathias B. Bertelsen[1], Michael Lisurek[1], Jan Vincent V. Arafiles[1], Jonathan Franke[1,2], Leander Crocker[1], Karl T. Schuppe[1,2], Yunjae Lim[1], Christiane Groneberg[3], Mathias Christmann[3], Han Sun[1,4], Christian P.R. Hackenberger[1,2*]

[1] Leibniz-Forschungsinstitut für Molekulare Pharmakologie (FMP), Robert-Rössle-Straße 10, 13125 Berlin, Germany.
E-mail: stieger@fmp-berlin.de, hackenbe@fmp-berlin.de

[2] Department of Chemistry, Humboldt Universität zu Berlin, Brook-Taylor-Straße 2, 12489 Berlin, Germany

[3] Institute of Chemistry and Biochemistry, Freie Universität Berlin, Takustraße 3, 14195 Berlin, Germany

[4] Institute of Chemistry, Technische Universität Berlin, Straße des 17. Juni 135, Berlin 10623, Germany

Contents

1. Supplementary Schemes 4

2. Supplementary Figures 5

2.1 Supplementary Figure S1: Proteome-wide cysteine labeling using 1a, 2a & 3a in RAMOS lysate 5

2.2 Supplementary Figure S2: Live-cell cysteine labeling using 1a, 2a & 3a 5

2.3 Supplementary Figure S3: Covalent docking of the two different isomers of 4a onto the crystal structure of EGFR 6

2.4 Supplementary Figure S4: Labeling of A431-cells by phosphonamidate-probe 7 8

2.5 Supplementary Figure S5: Concentration dependent EGFR labeling in A431 cells by 7 9

2.6 Supplementary Figure S6: Time-dependent labeling of A431-cells by 5 & 7 10

2.7 Supplementary Figure S7: Off-target reactivity of 5 & 7 in EGFR-negative RAMOS-cells 11

2.8 Supplementary Figure S8: Proteome-wide selectivity of 5 & 7 in A431 cells (1). 12

2.9 Supplementary Figure S9: Proteome-wide selectivity of 5 & 7 in A431 cells (2). 12

2.10 Supplementary Figure S10: Direct comparison of the proteome-wide reactivity of 5 & 7. . 13

2.11 Supplementary Figure 11: Comparison of Ibrutinib-probe 12 and PA-analogues 8-11 in RAMOS cell lysate 14

2.12 Supplementary Figure 12: Direct comparison of the proteome-wide reactivity of 12 & 9. . 15

2.13 Supplementary Figure 13: RAMOS cell lysate labeling using fluorescein-functionalized probe 13 16

3. General Information 17

3.1 Chemicals and Solvents 17

3.2 Flash- and thin layer chromatography 17

3.3 Semi-preparative HPLC 17

3.4 NMR-Spectroscopy 17

3.5 UPLC-UV/MS 17

3.6 HR-MS 18

3.7 Cell culture 18

3.8 Protein concentration determination 18

3.9 Covalent Docking 18

4. Experimental procedures 19

4.1 Determination of the glutathione half-life of different electrophiles 19

4.2 Testing the reactivity of different electrophilic groups on live cells and in lysate 19

4.3 Sample preparation and analysis of scout-frgament ABPP 22
4.4 In-cell protein labeling and in-gel fluorescence analysis 23
4.5 In-cell protein labeling and sample preparation for proteomic pull-down 24

5. Chemical Synthesis 26
5.1 Synthesis of Small-Molecule Electrophiles 26
5.2 Electrophilic “scout-fragments” 27
5.3 General procedure for phosphonite synthesis: 30
5.4 General Procedure for Staudinger-phosphonite reaction: 30
5.5 Synthesis of Phosphonamidate Analogues of Afatinib 31
5.6 Synthesis of vinyl-phosphonamidate based EGFR-probes 33
5.7 Synthesis of azide precursors for Ibrutinib analogues: 34
5.8 General Procedure for aryl-azide synthesis: 34
5.9 General Procedure for the synthesis of benzyl chlorides: 34
5.10 Synthesis of phosphonamidate-analogues of Ibrutinib 37

6. Supporting References 42

1. Supplementary Schemes

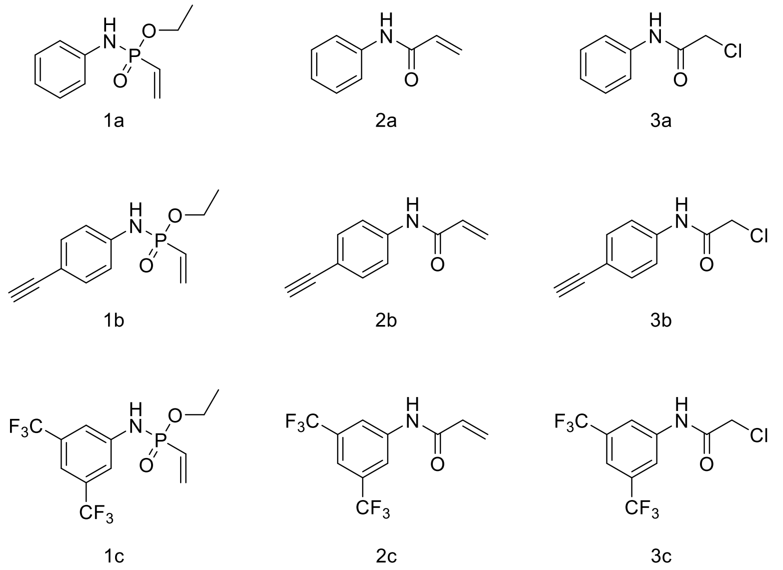

Scheme S1: *Chemical structure of compounds used in Figure 2.*

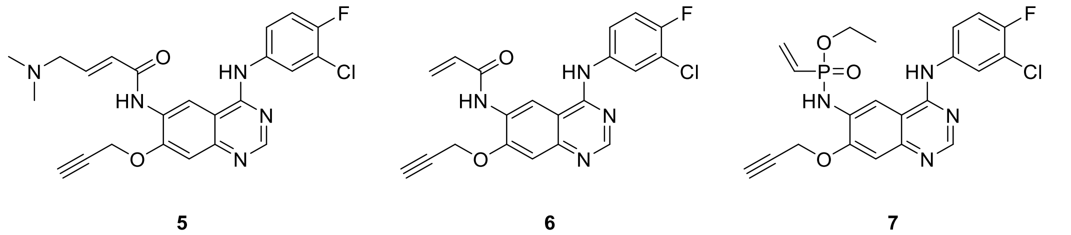

Scheme S2: *Chemical structure of additional compounds used in Figure 3.*

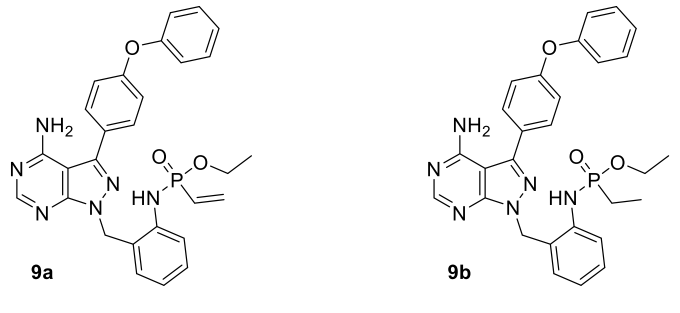

Scheme S3: *Chemical structure of additional compounds used in Figure 4.*

2. Supplementary Figures

2.1 **Supplementary Figure S1:** Proteome-wide cysteine labeling using **1a**, **2a** & **3a** in RAMOS lysate

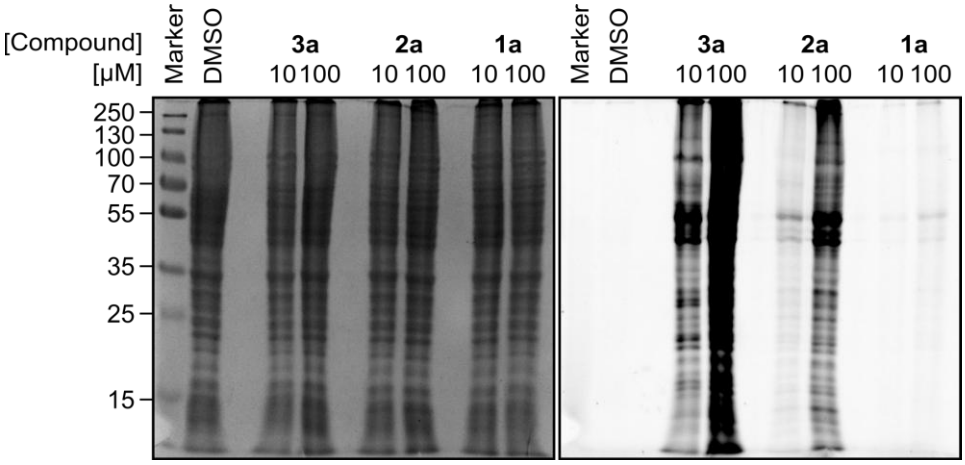

Figure S1: *RAMOS cell-lysate was treated with the indicated concentration of electrophiles for 1 h at 37 °C followed by CuAAC with Fluorescein-N_3 (See 0 for detailed procedure) and analyzed via SDS-PAGE. (left: Commassie stainig; right: In-gel fluorescence signal)*

2.2 **Supplementary Figure S2**: Live-cell cysteine labeling using **1a, 2a & 3a**

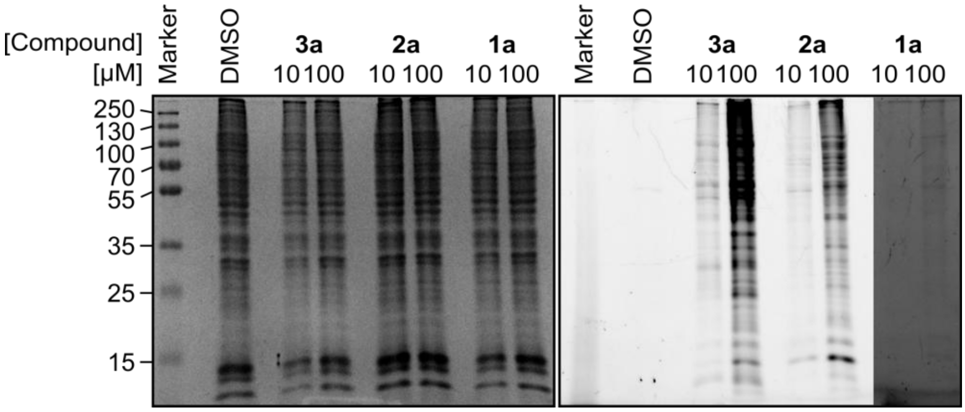

Figure S2: *RAMOS cells were treated with the indicated concentration of electrophiles for 1 h at 37 °C followed by lysis and CuAAC with Fluorescein-N_3 (See 0 for detailed procedure). Lysates were analyzed via SDS-PAGE. Contrast for **1a** had to be adjusted differently to see signal. (left: Commassie stainig; right: In-gel fluorescence signal)*

2.3 **Supplementary Figure S3:** Investigating the cellular permeability of VPAs using a chloroalkane permeability assay (CAPA)

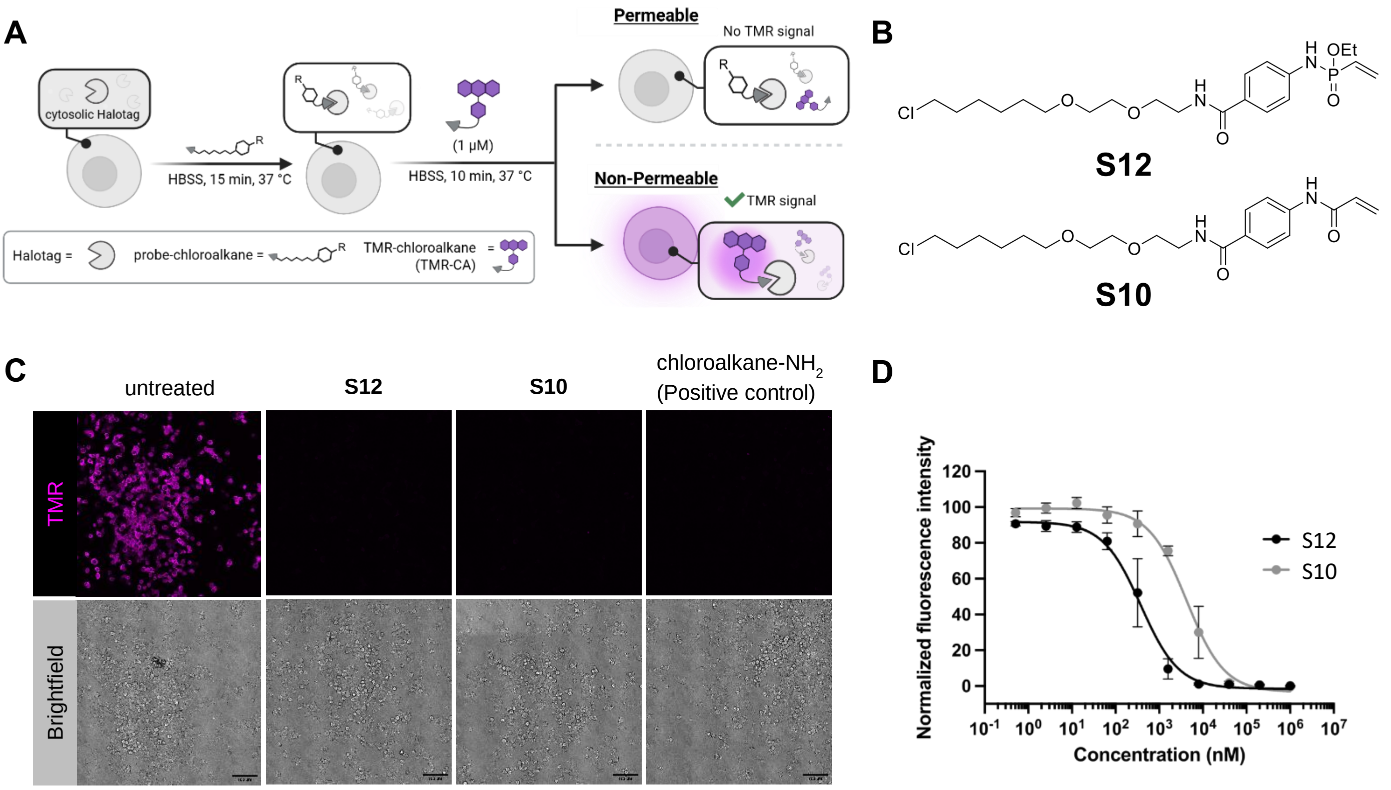

Figure S3: *a) Schematic representation of the CAPA assay used in this study. Permeability is validated by treatment with TMR-CA. b) Chemical structure of the used VPA and the corresponding acrylamide HaloTag-ligand. c) Tile-scan confocal microscopy images of modified CAPA reporter HeLa cells treated with **S12**, **S10**, or chloroalkane-amine positive control (10 µM) in Hank's balanced salt solution (HBSS) for 15 min, followed by TMR-CA (1 µM) for 10 min. (Scale bars = 100 µm). f) Normalized fluorescence intensity (TMR) of reporter HeLa cells following treatment with **S12** and **S10** ($0.5e^{-3}$, $2.56e^{-3}$, $12.8e^{-3}$, 0.064, 0.32, 1.6, 8, 40, $2e^{2}$ and $1e^{3}$ µM), monitored using TMR-CA (1 µM) were determined via flow cytometry. (**S12** CP_{50} = 0.36 µM; **S10** CP_{50} = 4.2 µM) Data points are presented as the mean ± standard deviation of three biological replicates. Fore experimental details see section 0.*

2.4 **Supplementary Figure S4:** Covalent docking of the two different isomers of **4a** onto the crystal structure of EGFR

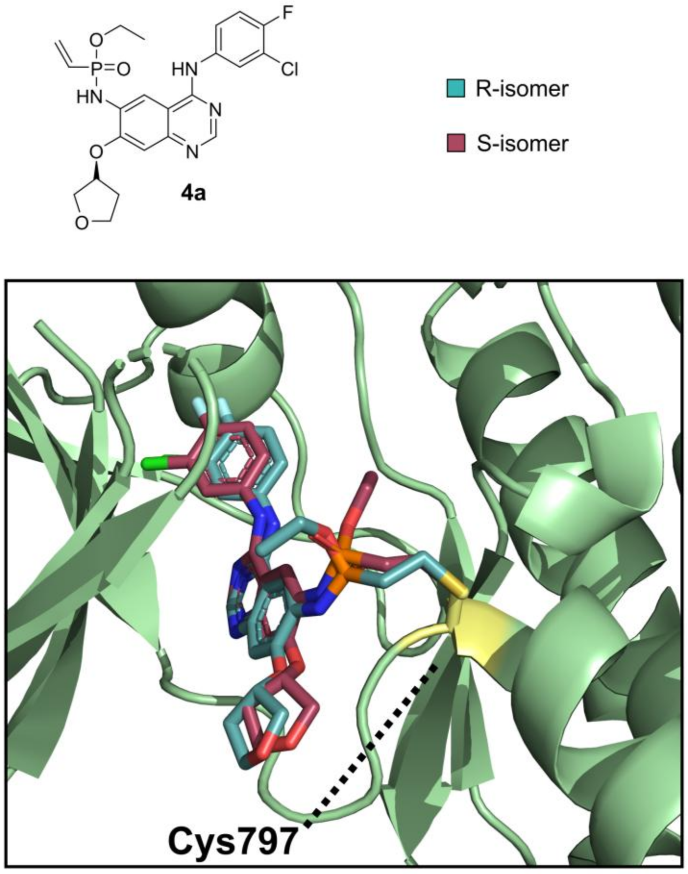

Figure S4: *Super positioning of the best pose obtained from the covalent docking of R- & S-**4a** onto the crystal structure of human EGFR (PDB: 4G5J). Both isomers fit equivalently well into the ATP-binding pocket indicating no significant difference in their ability to target EGFR.*

2.5 **Supplementary Figure S5:** Determination of the labeling kinetics of **4a** and Afatinib with wt EGFR

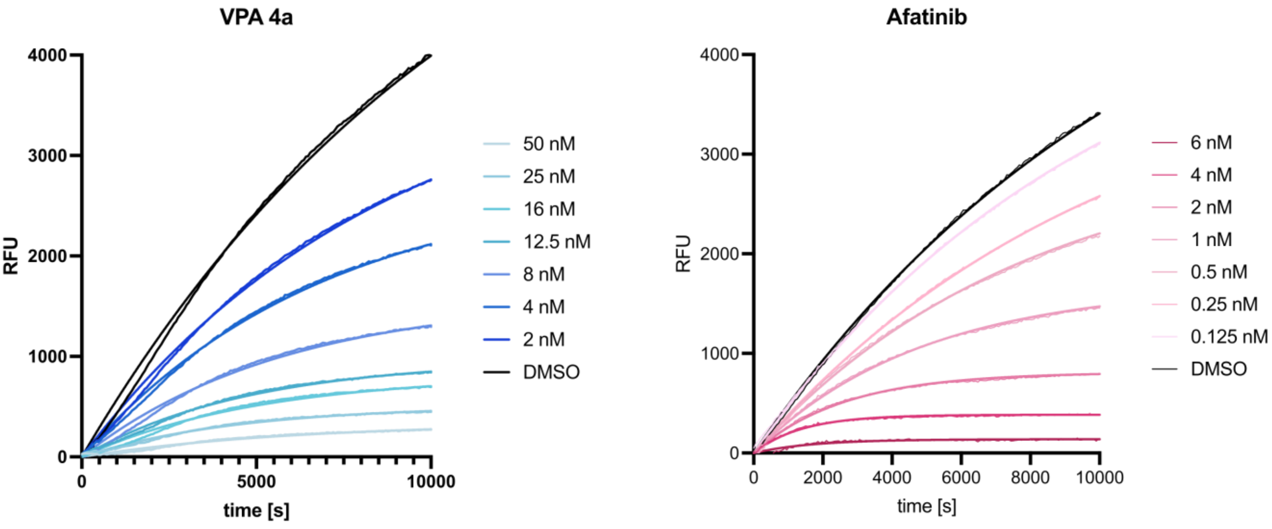

Figure S5: *Determination of k_{inact} and K_I parameters of probe **4a** and Afatinib using the PhosphoSense® kinase assay kit (based on Shults et al.[1]) Graphs show the global fit of the reaction progress from inhibition of wt EGFR by **4a** or Afatinib. Respective inhibitor concentrations are shown in the inset. For experimental details see section 0.*

2.6 **Supplementary Figure S6:** Labeling of A431-cells by phosphonamidate-probe **7**

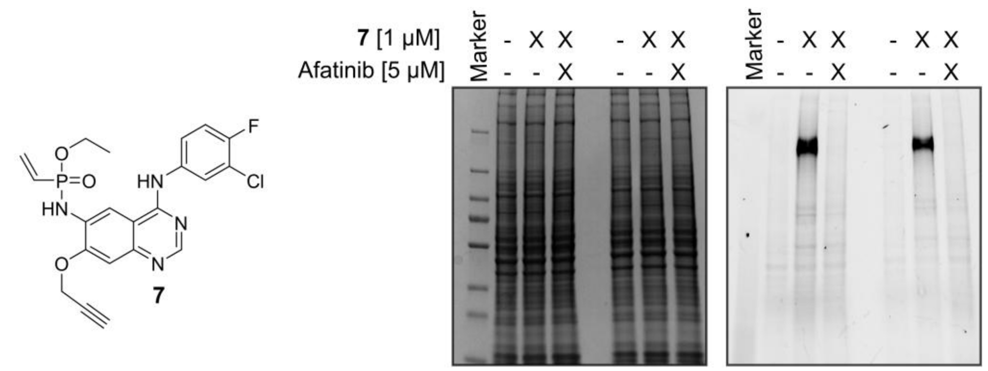

Figure S6: *Investigating the EGFR-selective labeling of vinyl-phosphonamidate probe **7** on A431 cells. Cells were grown to 80-90% confluence and treated with the indicated compound concentration for 1 h at 37 °C in RPMI w/o FCS. Afterwards, cells were processed according to the procedure described in 4.7. (left: Commassie stainig; right: In-gel fluorescence signal)*

2.7 **Supplementary Figure S7**: Concentration dependent EGFR labeling in A431 cells by **7**

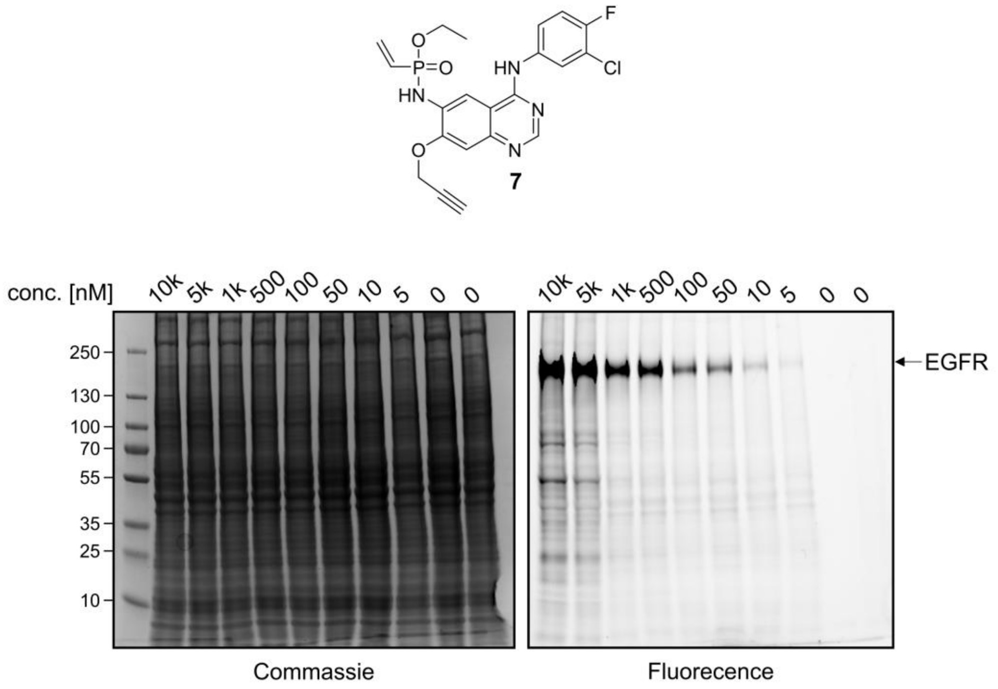

Figure S7: *Concentration dependent labeling of EGFR in A431 cells by the phosphonamidate based probe **7**. Cells were grown to 80-90% confluency and treated with the indicated probe-concentration for 1 h @ 37 °C in RPMI w/o FCS. After treatment, cells were processed according to the procedure described in 4.7. (left: Commassie stainig; right: In-gel fluorescence signal)*

2.8 **Supplementary Figure S8:** Time-dependent labeling of A431-cells by **5** & **7**

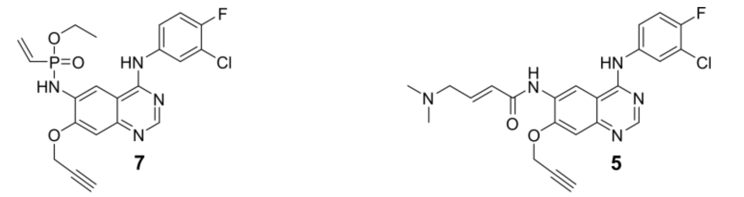

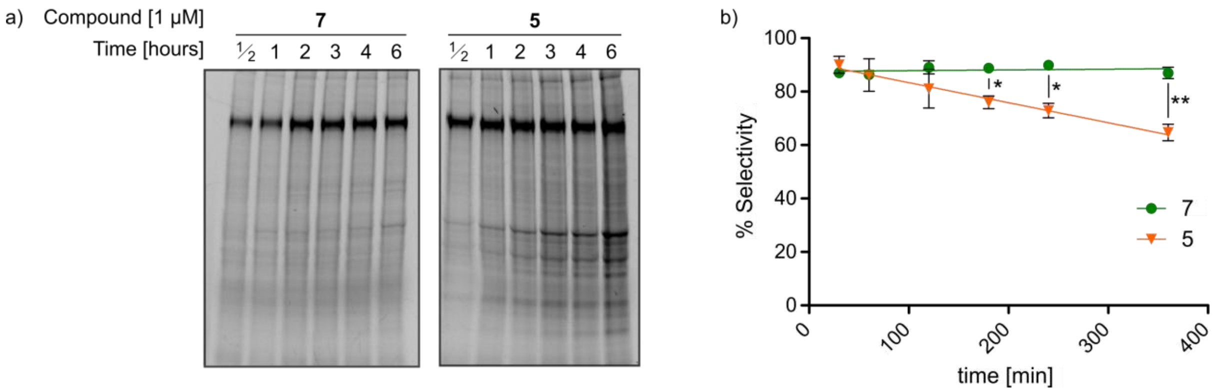

Figure S8: a) *Time course experiment of A431-cells incubated with 1 µM of the corresponding compound. Cells were lysed at the indicated time point and processed according to the procedure described in 4.7. Gels are representative for two independent experiments.* ***b)*** *Averaged target selectivity for replicate experiments. Selectivity = (Intensity EGFR band)/(Total lane intensity)*

2.9 **Supplementary Figure S9:** Off-target reactivity of **5** & **7** in EGFR-negative RAMOS-cells

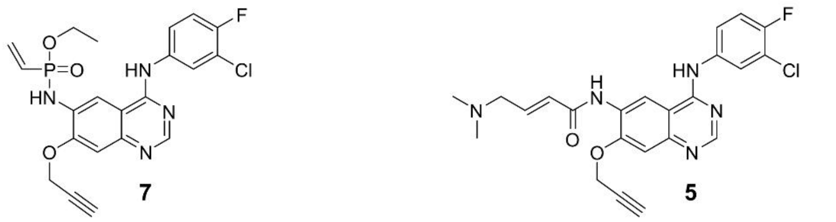

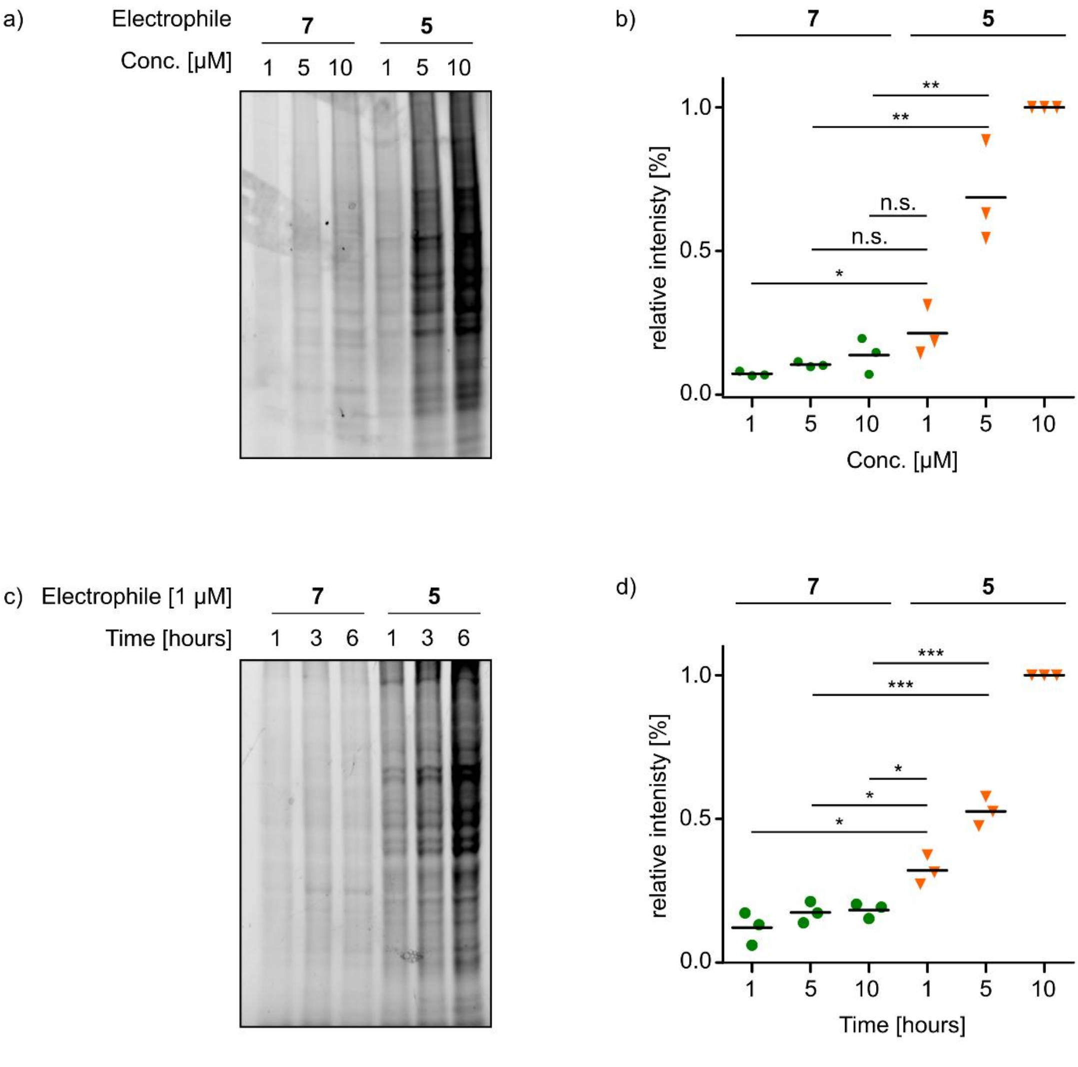

Figure S9: *Concentration dependent off-target reactivity of vinyl-phosphonamidate-probe **7** & Afatinib-probe **5** in EGFR-negative RAMOS-cells. Cells (3x10^6/ml) were treated with the indicated probe-concentration for 1 h @ 37 °C in RPMI –FCS. After the treatment, cells were processed according to the procedure described in 4.7. Data is representative of three independent experiments. a) Fluorescent gel of RAMOS-cells treated with the indicated probe-concentration. b) Relative fluorescent intensity of three independent experiments c) Fluorescent gel of RAMOS-cells treated 1 µM probe **5** or **7** for the indicated time. b) Relative fluorescent intensity of three independent experiments (quantitative results were normalized to the most intense band in each replicate)*

2.10 **Supplementary Figure S10:** Proteome-wide selectivity of **5** & **7** in A431 cells **(1).**

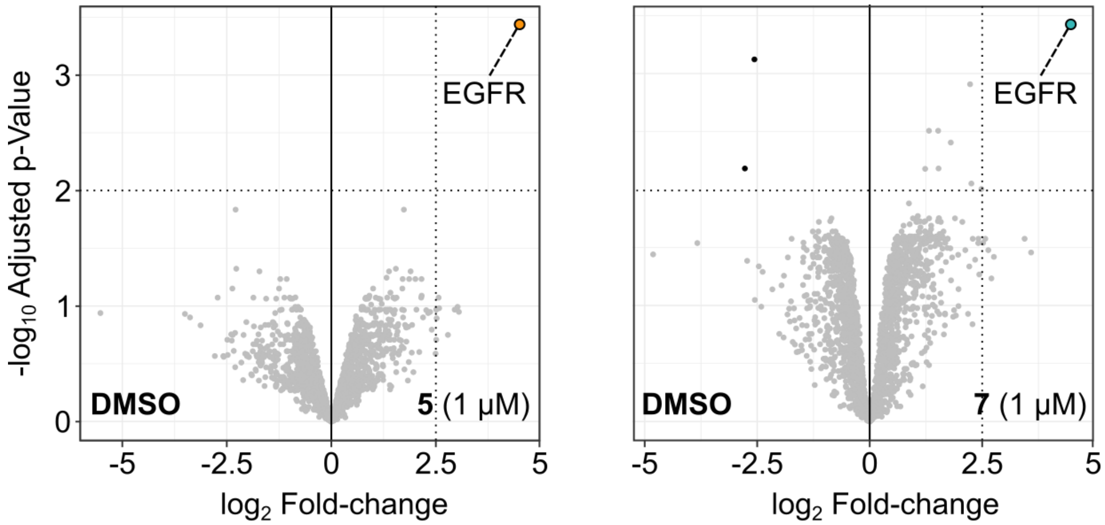

Figure S10: *Volcano blots obtained from label free quantification of the proteins from streptavidin pulldowns performed with 1 µM probe **5** or **7** (in comparison to the DMSO treated sample). Proteins were identified and quantified and blotted as described in 4.8: Variance stabilization and Perseus-type imputation were enabled. Proteins surpassing a log_2 fold-change of 2.5 and a $-log_{10}$ adjusted p-value of 2 were considered to be significantly enriched. At the tested concentration, both probes solely enrich EGFR. Data was obtained from 4 independent replicates.*

2.11 **Supplementary Figure S11:** Proteome-wide selectivity of **5** & **7** in A431 cells **(2).**

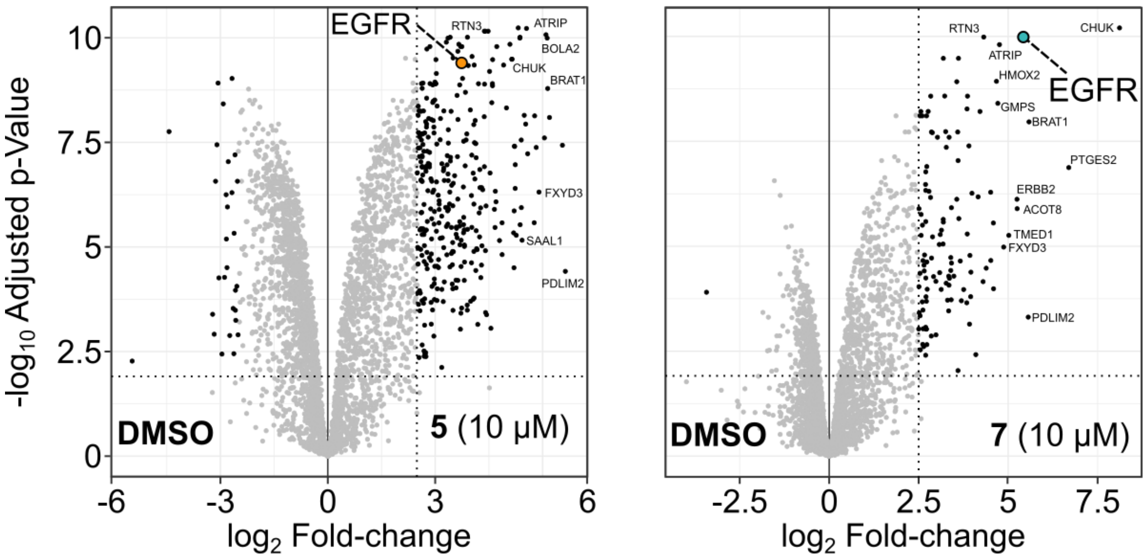

Figure S11: *Volcano blots obtained from label free quantification of the proteins from streptavidin pulldowns performed with 10 µM probe **5** or **7** (in comparison to the DMSO treated sample). Proteins were identified and quantified and blotted as described in 4.8: Variance stabilization and Perseus-type imputation were enabled. Proteins surpassing a log_2 fold-change of 2.5 and a $-log_{10}$ adjusted p-value of 2 were considered to be significantly enriched. A full list of identified and significantly enriched proteins can be found in Supplementary Table 1. Data was obtained from 4 independent replicates.*

2.12 **Supplementary Figure S12:** Direct comparison of the proteome-wide reactivity of **5** & **7.**

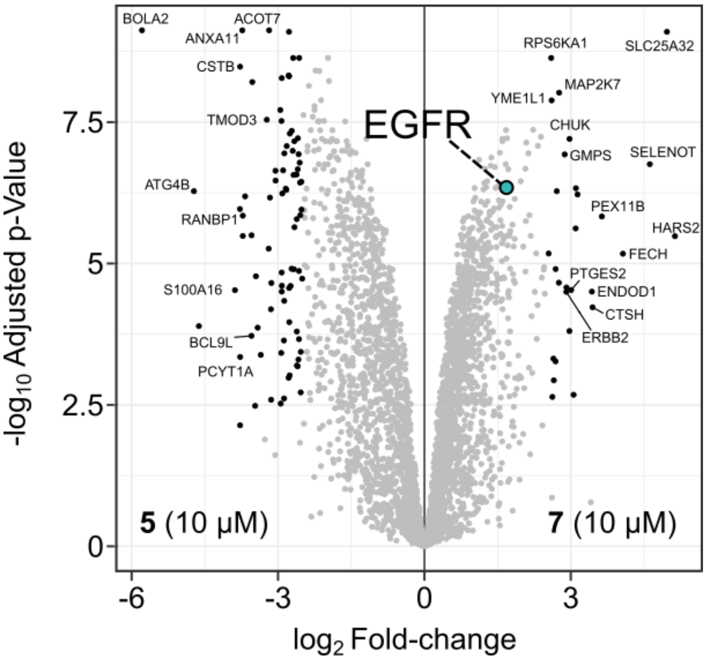

Figure S 12: *Volcano blot was obtained from a direct comparison of proteins pulled down by probes **5** and **7** (10 µM probe concentration). Proteins were identified, quantified and blotted as described in 2.11 and 4.8. A full list of identified and significantly enriched proteins can be found in Supplementary Table 1. Data was obtained from 4 independent replicates.*

2.13 **Supplementary Figure 13:** Comparison of Ibrutinib-probe **12** and PA-analogues **8-11** in RAMOS cell lysate

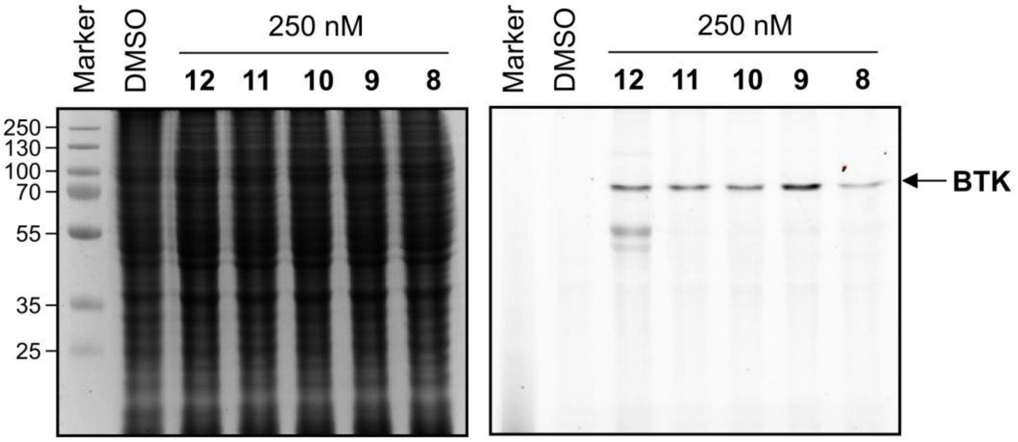

Figure S13: *Labeling of BTK in RAMOS cell lysate (1 mg/ml) by PF-06658607 (**12**) and phosphonamidate based probe **8-11** with the indicated probe-concentration for 1 h @ 37 °C. After treatment, lysate was clicked to FAM-N_3 and analyzed by SDS-PAGE. (left: Commassie stainig; right: In-gel fluorescence signal)*

2.14 **Supplementary Figure 14:** Direct comparison of the proteome-wide reactivity of **12** & **9.**

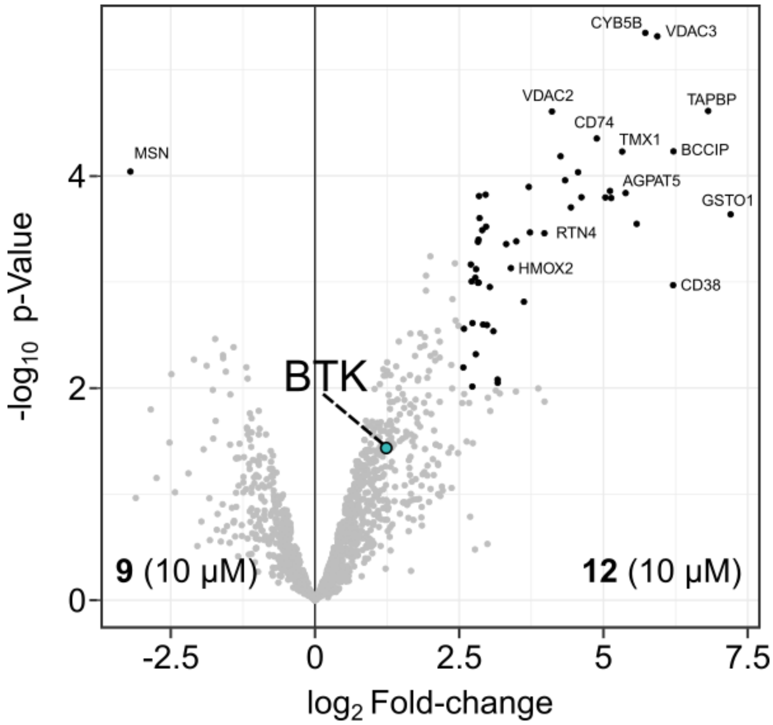

***Figure S14:** Volcano blot was obtained from a direct comparison of proteins pulled down by probes **12** and **9** (10 µM probe concentration) from RAMOS cells. Proteins were identified, quantified and blotted as described in 4.5 (imputation was disabled) and proteins with a log_2 fold-change >2.5 and a p-value <0.01 were considered to be significantly enriched. Data was obtained from 3 independent replicates.*

2.15 **Supplementary Figure 15:** RAMOS cell lysate labeling using fluorescein-functionalized probe **13**

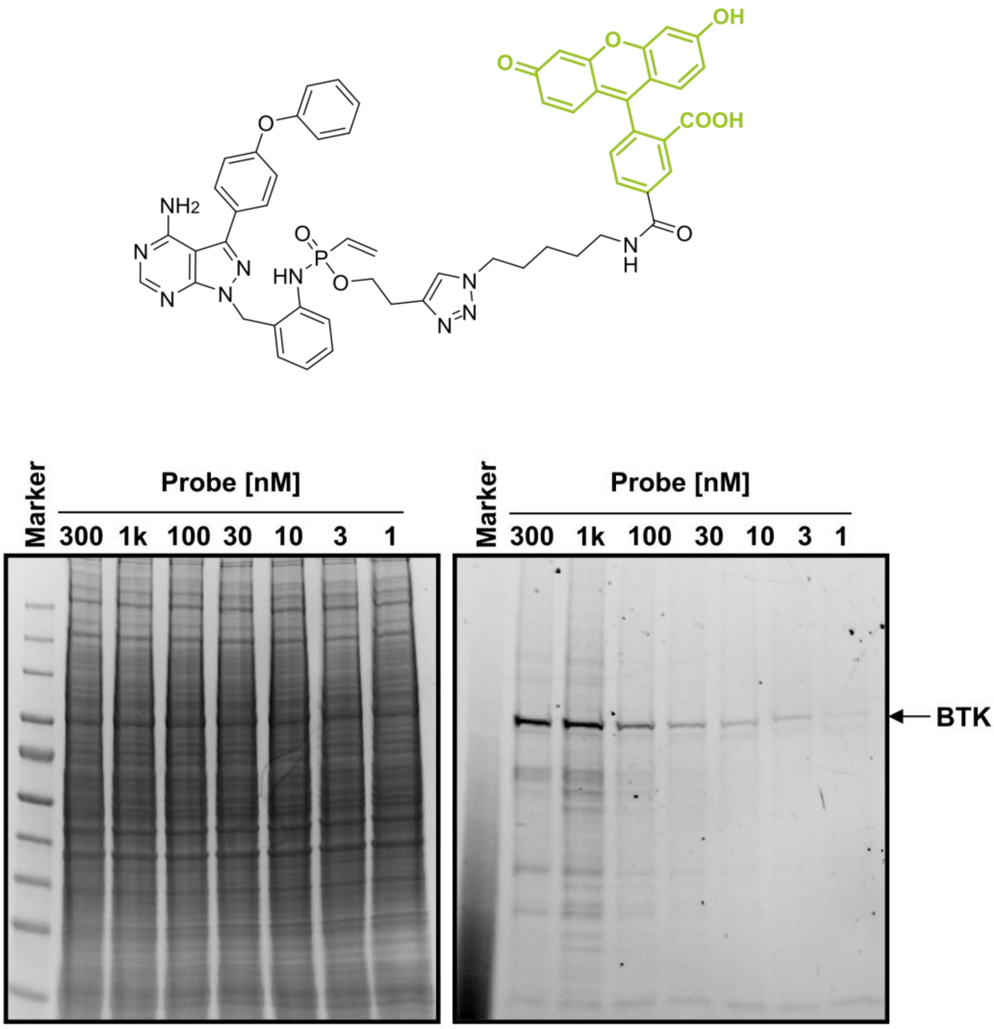

Figure S15: Concentration dependent labeling of BTK in RAMOS cell lysate (1 mg/ml in PBS) by the phosphonamidate based probe **13**. *Lysate was incubated with the indicated probe-concentration for 1 h @ 37 °C followed by CuAAC to FAM-N_3 and analysis by SDS-PAGE. (left: Commassie stainig; right: In-gel fluorescence signal)*

3. General Information

3.1 Chemicals and Solvents

Chemicals and solvents were purchased from Merck (Merck group, Germany), TCI (Tokyo chemical industry CO., LTD., Japan), BLDpharm (BLD Pharmatech GmbH, Germany) and Acros Organics (Thermo Fisher scientific, USA) and used without further purification. Dry solvents were purchased from Acros Organics (Thermo Fisher scientific, USA). Amino acids and resins for SPPS were purchased from Novabiochem (Merck, USA) or Iris Biotech GmbH (Germany).

3.2 Flash- and thin layer chromatography

Flash column chromatography was performed, using NORMASIL 60® silica gel 40-63 µm (VWR international, USA). Glass TLC plates, silica gel 60 W coated with fluorescent indicator F254s were purchased from Merck (Merck Group, Germany). Spots were visualized by fluorescence depletion with a 254 nm lamp or manganese staining (10 g K_2CO_3, 1.5 g $KMnO_4$, 0.1 g NaOH in 200 ml H_2O), followed by heating.

3.3 Semi-preparative HPLC

Semi-preparative HPLC was performed on a Shimadzu prominence HPLC system (Shimadzu Corp., Japan) with a CBM20A communication bus module, a FRC-10A fraction collector, 2 pumps LC-20AP, and a SPD-20A UV/VIS detector, using a VP250/21 Macherey-Nagel Nucleodur C18 HTec column (Macherey-Nagel GmbH & Co. Kg, Germany).

3.4 NMR-Spectroscopy

NMR spectra were recorded with a Bruker Ultrashield 300 MHz spectrometer and a Bruker Avance III 600 MHz spectrometer (Bruker Corp., USA) at ambient temperature. Chemical shifts δ are reported in ppm relative to residual solvent peak ($CDCl_3$: 7.26 [ppm]; DMSO-d_6: 2.50 [ppm]; acetone-d_6: 2.05 [ppm]; CD_3CN 1.94 [ppm]; 4.79 D_2O [ppm] for ^{1}H-spectra and $CDCl_3$: 77.16 [ppm]; DMSO-d_6: 39.52 [ppm]; acetone-d_6: 29.84 [ppm]; CD_3CN 1.32 [ppm]; for ^{13}C-spectra. Coupling constants J are stated in Hz. Signal multiplicities are abbreviated as follows: s: singlet; d: doublet; t: triplet; q: quartet; m: multiplet.

3.5 UPLC-UV/MS

UPLC-UV/MS traces were recorded on a Waters H-class instrument equipped with a quaternary solvent manager, a Waters autosampler, a Waters TUV detector and a Waters Acquity QDa detector with an Acquity UPLC BEH C18 1.7 µm, 2.1 x 50 mm RP column with a flow rate of 0.6 mL/min (Waters Corp., USA). The following gradient was used: Gradient A: 0.1% TFA in H_2O; B: 0.1% TFA in MeCN. 5% B 0 - 1.5 min, 5-95% B 1.5-13 min, 95% B 13-13.9 min, 5% B 13.9-15 min. Gradient B: 0.1%

TFA in H_2O; B: 0.1% TFA in MeCN. 5% B 0 -0.5 min, 5-95% B 0.5-3 min, 95% B 3-3.9 min, 5% B 3.9-5 min.

3.6 HR-MS

High resolution ESI-MS spectra were recorded on a Waters H-class instrument equipped with a quaternary solvent manager, a Waters sample manager-FTN, a Waters PDA detector and a Waters column manager with an Acquity UPLC protein BEH C18 column (1.7 µm, 2.1 mmx 50 mm). Samples were eluted with a flow rate of 0.3 mL/min. The following gradient was used: "QTof": 0.01% FA in H_2O; B: 0.01% FA in MeCN. 5% B: 0-1 min; 5 to 95% B: 1-4min; 95% B: 4 to 4.5 min. Mass analysis was conducted with a Waters XEVO G2-XS QTof analyzer.

3.7 Cell culture

RAMOS, A431 and H292 cell lines were purchased from the American Type Culture Collection (ATCC, USA) and cultured at 37 °C under a humidified 5% CO_2 atmosphere in RPMI1640 medium (Invitrogen) supplemented with 10% FCS and 0.5% Penicillin-Streptomycin (Invitrogen). Cells were cultured in T75 cell culture flasks and used up until passage 20.

3.8 Protein concentration determination

Protein concentrations were determined by BCA assay (Thermo Fisher Scientific, USA) according to the manufacturer's protocol.

3.9 Covalent Docking

The binding mode of Afatinib-derived ligands (afatinib, **4**(*R*) and **4**(*S*)) to wt-EGFR were modelled using the docking program GOLD (version 2021.2.0, Cambridge Crystallographic Data Center)[2] integrated in the software package DiscoveryStudio (BIOVIA), the ChemPLP scoring function was employed.[3] The binding site was selected from the PDB site record from 4G5J[4] and a covalent restraint on the thioether bond between the ligands and Cys797 was applied. Structures were visualized using PyMOL Molecular Graphics System (Version 2.0, Schrödinger). The same procedure was performed using Ibrutinib and 9(*R*/*S*) with the PDB site record from 5P9J.[5]

4. Experimental Procedures

4.1 Determination of the glutathione half-life of different electrophiles

Freshly dissolved red. glutathione (50 mM) was neutralized using 2 M NaOH. Comopunds were dissolved in PBS (10 mM, 100 mM NaCl, pH 7.4, 10 vol% MeCN) at a concentration of 1.25 mM alongside 1-2 mM coffeine (depending on the UV absorbtion of the corresponding compound) as internl standard. The reaction was initiated by the addition of red. glutathione (10 mM final concentration) and incubated at room temperature. At the indicated time points, an aliquot was taken, quenched by the addition of Na-acetate (pH 4) and analyzed via UPLC-UV/MS. The t_0 timepoint was taken before the addition of GSH. Data was blotted in Graphpad Prism 5.04 and fitted to a one-phase decay. Results were obtained from independent triplicates.

4.2 Determination of lysine reactivity of compound 1a

The assay was performed analogously to glutathion reactivity using L-lysine. In addition to pH 7.4 we also evaluated the reactivity at a more basic pH of 8.5. The reaction was monitored for a total of 5 days and aliquotes were taken at indicated timepoints and analyzed via UPLC-UV/MS.

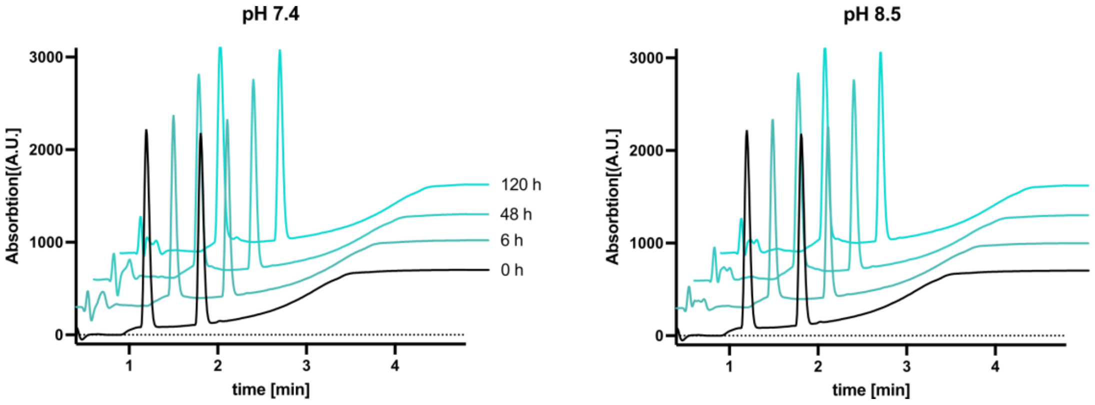

Figure S16: *Reactivity of VPA **1a** with unprotected L-lysine. Time-course of the reaction between **1a** and 10 eq. unprotected L-lysine at pH 7.4 (left) and pH 8.5 (right). Over the monitored periode of 120 h, no reaction- or degradation-products could be detected. (the first peak corresponds to the internal standard caffeine, the second peak to **1a**)*

4.3 Testing the reactivity of different electrophilic groups on live cells and in lysate

Labeling in lyasate:

RAMOS cells were grown in T75 suspension cell culture flasks to an approx. density of $3x10^6$ cells/ml. Cells were harvested by centrifugation, washed trice with ice cold PBS (Ph 7.4) and stored at -70°C until lysis. For cell-lysis, the pellet was resolubilized in ice cold PBS, transferred to 1.5 ml Eppendorf-flasks and sonicated with a Bioruptor (5 min, cycle 30/30 s, 4°C). Lysates were cleared by centrifugation (15'000 rcf) and the proteinconcentration was determined via BCA assay (Pierce BCA Protein Assay Kit, Thermo Fisher Scientific) according to the vendors instructions. The protein concentration was adjusted to 1 mg/ml with PBS and aliquotes were frozen in liquid nitrogen and stored at -70°C until further usage.

Aliquotes of RAMOS cell lysate were thawed on ice and aliquoted à 100 µg of protein and samples were incubated with either the chloroacetamide (**1b**), acrylamide (**2b**) or phosphonamidate-based alkyne (**3b**) at a final concentration of 100 or 10 µM (1 h, 37 °C, 800 rpm). Subsequently, lysates were clicked to fluorescein-N_3 (final concentration of 1.5 mM $CuSO_4$, 0.1 mM TBTA, 0.5 mM TCEP, 0.05 mM fluorescein-N_3) in a shaker (800 rpm, 37 °C) for 45 minutes. Finally, lysates were precipitated with the 4-fold volume of acetonitrile and cetrifuged (14'000 rcf, 5 min). The solvent was removed and precipitates were resuspended in 80% EtOH followed by another round of centrifugation (10'000 rcf, 5 min).

Labeling in cells:

$6x10^6$ Ramos cells (in 2 mL RPMI 1640 without FCS) were treated with either the chloroacetamide (**1b**), acrylamide (**2b**) or phosphonamidate-based alkyne (**3b**) at a final concentration of 100 or 10 µM (1 h, 37 °C, 800 rpm). Afterwards, the cells were collected by centrifugation (1000 rcf for 5 min.), and washed once with PBS. Next, cells were lysed using RIPA lysis buffer (80 uL, containing 50 mM *N*-ethylmaleimide, 1x protease inhibitor (cOmplete^{TM}, Roche) and 1:2000 benzoase) for 30 minutes at 37 °C. Lysates were transferred into a 2 ml Eppendorf tube and clicked to fluorescein-N_3 (final concentration of 1.5 mM $CuSO_4$, 0.1 mM TBTA, 0.5 mM TCEP, 0.05 mM fluorescein-N_3) in a shaker (800 rpm, 37 °C) for 45 minutes. Frome here, samples were treated as described for cell lysate experimets.

4.4 Modified chloroalkane penetration assay (CAPA)

This method was adopted from previous reports.[6,7] CAPA reporter cells were prepared by transfecting HEK293T with pcDNA5-HaloTag using Lipofectamine 3000 (Thermo) according to manufacturer's protocol. After 24 h, reporter cells were trypsinized and seeded into specified vessels below.

For confocal imaging, reporter cells were seeded onto 8-well glass bottom slides (IBIDI) and allowed to attach for 24 h. Cells were washed twice with PBS followed by treatment with 100 µM of **S12**, **S10** or chloroalkane-NH_2 (positive control) in Hank's balanced salt solution (HBSS) for 15 min at 37 °C. Cells were washed with PBS and treated with 1 µM of tetramethyrhodamine-chloroalkane (MedChemExpress) in HBSS for 10 min at 37 °C. Excess treatment reagent were washed off using PBS. Tile images were captured using a Nikon microscope with a CSU-X1 (Andor) confocal laser scanning system and a live-cell incubation chamber (OKOlab). Images shows are acquired using a 40x, 1.4-NA Plan-Apochromat objective (Nikon) and an EMCCD (AU888, Andor). Brightfield images were acquired along with fluorescence images. Standard laser, a quad Dicroic (560-570) and Emission filters were used in the acquisition of confocal fluorescence images [RFP (TMR), ex.: 561 em.:600/50 nm]. Scale bars = 100 µm.

To determine the CP50 (concentration at which 50% cell penetration for specified cell line), reporter cells were seeded into 24-well plates (3 × 105 cells/well). After 24 h, reporter cells were washed twice with PBS followed by the treatment with 10 µM positive control, and 1000000, 200000, 40000, 8000, 1600, 320, 64, 12.8, 2.560, 0.512 nM of **S12** or **S10** in HBSS for 15 min at 37 °C. Cells were washed and treated with 1 µM TMR-CA in HBSS for 10 min at 37 °C. Treated reporter cells were trypsinized, collected in 1.5 mL microcentrifuge tubes, washed with PBS, and pelleted by centrifugation (800 ×g, 5 min, 4 °C). Cell pellets were resuspended in 400 µL PBS and were analysed by flow cytometry.

Median fluorescence intensities (MFI) in TMR of gated cells were determined. The equation below was used to determine the Normalized fluorescence intensity (%) :

$$\text{Normalized fluorescence instensity (\%)} = \frac{(\mathbf{S12}\text{ or }\mathbf{S10}\ \mathrm{MFI_{TMR}} - \text{positive control } \mathrm{MFI_{TMR}})}{(\text{untreated cell } \mathrm{MFI_{TMR}} - \text{positive control } \mathrm{MFI_{TMR}})} \times 100$$

The normalized fluorescence intensities were plotted using GraphPad Prism 10 with a non-linear curve fitting. The IC_{50} determined from this curve represents the CP_{50} value. Data represents the mean of three independent biological replicates ± standard error.

4.5 Sample preparation and analysis of scout-frgament ABPP

HEK293 cells were grown in T75 cell culture flasks to approx. 80% confluency. The cells were washed with PBS and lysed in 50 mM Tris pH 7.5 containing 150 mM NaCl, 5 mM EDTA, 1 mM PMSF and 0.5% Triton X-100 by sonication. The total amount of the protein was determined by BCA assay and stored at -80 °C until usage.

The following procedure represents a combination of procedures described by Zanon et al.[8] and Yan et al.[9] Cell-lysates were dilluted to a final protein concentration of 1 mg/ml using PBS pH 7.4 and incubated with 100 mM of the corresponding electrophilic scout fragment or DMSO (5 vol% DMSO final, 1 h, 37 °C). After this, samples were treated individually with 0.1 mM iodoacetamide-alkyne and after another 45 minutes at r.t. the “click-mix” was added (final concentration of 1.5 mM $CuSO_4$, 0.1 mM TBTA, 0.5 mM TCEP, 0.1 mM of the corresponding heavy or light isoDTB tag). After 45 minutes at 37 °C, the “heavy” and “light” samples were mixed 1:1 and a mixture of prewashed hydrophilic & hydrophobic magnetic beads (Seramag™, Cytavia) was added. Proteins were precipitated onto the beads by the addition of the 4-fold volume absolute EtOH. Magnetic beads were capured, washed 3x with 80% EtOH and redissolved in 8 M urea at pH 8. After reduction (10 mM DTT, 56 °C, 30 min) and alkylation (20 mM iodoacetamide, r.t., 30 min) proteins were once more precipitated onto the magnetic beads and washed 3x with 80% EtOH. Finally, proteins were redissolved in 2 M urea (pH 8) and trypsinized overnight at 37 °C. Peptides were precipitated onto the magnetic beads by the addition of acetonitrile to a final concentration of 95%, followed by three washes with 100% acetonitrile. Tryptic peptides were eluted from the magnetic beads by two sequential elution with PBS containing 2% DMSO and incubated with high-capacity streptavidin (Thermo Fisher Scientific) beads for 1 h at r.t. while shaking at 800 rpm. Beads were collected by centrifugation and washed sequentially with PBS (3x) and water (6x). Bound peptides were eluted by two times the addition of acetonitrile/water (1:1) containing 0.1% TFA (100 µl, 2x). The solvent was removed in a speedvac and peptides were dissolved in water containing 2% acetonitrile and 0.1% TFA and stored at -20 °C until measurement.

4.6 PhosphoSense® kinase assay

PhosphoSense® kinase assay kits were obtained from AssayQuant, containing the appropriate peptide substrate for the respective enzyme. Recombinant EGFR was purchased from Sino Biological. Assays were performed according to the manufacturer's instructions. Briefly, reactions contained peptide substrate (c_{final} = 10 µM), ATP (c_{final} = 1 mM), EGTA (c_{final} = 550 µM), and reaction buffer (50 mM HEPES, pH 7.5, 0.1% Brij-35, 100 mM $MgCl_2$) in the presence of inhibitors at the indicated concentrations. All components were prepared in a white 384-well plate by addition of 10× stock solutions of each reagent (final DMSO concentration = 1%) and equilibrated at 30 °C. Reactions were initiated by adding enzyme to a final concentration of 2.5 nM in 25 µL reaction volume.
Fluorescence was monitored over 4 h at 30 °C every 60 seconds (λ_{ex} = 360 nm and λ_{em} = 492 nm). Background-subtracted fluorescence intensity (RFU) was plotted against time (s), and data were fitted to a single-phase association model:

$$Y = (v_0 / k) \times (1 - e^{\wedge}(-k \times x))$$

using GraphPad Prism. The resulting rate constants (k) were plotted as a function of inhibitor concentration. For Afatinib, a linear regression was applied to determine k_{inact}/K_I from the slope of the fitted line. Data for compound 4a were fitted to the competitive inhibition rate equation:

$$Y = (k_{inact} \times X) / (X + K_I (1 + [S]/K_m))$$

with [S] = 10 µM, yielding values for k_{inact} and K_I.

4.7 In-cell protein labeling and in-gel fluorescence analysis

For EGFR-probes, A431-cells were seeded at 60'000 cells/well in a 6-well plate (Greiner Bio-One) and incubated until 80-100% confluencey. Before treatment, cells were washed twice with PBS. Cells were incubated with the indicated compound concentration (final conc. of 1% DMSO in RPMI -FCS) at 37 °C. After the incubation, cells were washed twice with ice cold PBS and lysed in ice-cold RIPA-buffer containing cOmplete™ protease inhibitor (Roche), 50 mM iodo acetamide (I1149, Sigma Aldrich) and Benzonase (1:1000; 70746, Merck). Following a 10 min incubation at 37 °C lysates were transferred into a 2 ml Eppendorf tube and clicked to fluorescein-N_3 (final concentration of 1.5 mM $CuSO_4$, 0.1 mM TBTA, 0.5 mM TCEP, 0.05 mM fluorescein-N_3) in a shaker (800 rpm, 37 °C) for 45 minutes. Afterwards, proteins were precipitated with the 4-fold volume of acetonitrile and cetrifuged (14'00 rcf, 5 min). The solvent was removed and precipitates were resuspended in 80% EtOH followed by another round of centrifugation (10'000 rcf, 5 min). The pellets were aspirated and left to dry on air for 5-10 minutes. Afterwards, the precipitate was redissolved in 40 µl 8 M urea (100 mM Tris, pH 8), 8 µl Laemmli buffer were added and the samples were denatured at 95 °C for 5 minutes. Proteins were seperated via SDS-PAGE (12% or 4-20% acrylamide gels) and analyzed on a Gel Doc XR+ (Bio-Rad, USA). If indicated, lanes and bands were quantified in ImageLab 6.1.

For BTK-probes, RAMOS-cells were seeded at 3x10^6 cells/ml (RMPI w/o FCS) and incubated with the indicated compound concentration (final conc. of 1% DMSO) at 37 °C. After the incubation, cells were harvested by centrifugation, washed twice with ice cold PBS and lysed in ice-cold RIPA-buffer containing cOmplete™ protease inhibitor (Roche), 50 mM iodo acetamide (I1149, Sigma Aldrich) and Benzonase (1:1000; 70746, Merck). Following a 10 min incubation at 37 °C lysates were further processed as described above.

4.8 In-cell protein labeling and sample preparation for proteomic pull-down

Cells were cultured, treated and lysed as described in **4.4**. Lysates (approx. 200 µg of protein each) were transferred into a 2 ml Eppendorf tube and clicked to biotin-N_3 (final concentration of 1.5 mM $CuSO_4$, 0.1 mM TBTA, 0.5 mM TCEP, 0.05 mM biotin-N_3) in a shaker (800 rpm, 37 °C) for 45 minutes. Afterwards, the samples were processed according to a procedure published by Becker et al.[10]: A total of 50 µl of mixed hydrophobic and hydrophilic carboxylate-coated magnetic beads (1:1, prewashed with PBS; Seramag™, Cytavia) was added to the click reaction mixture followed by 600 µl of absolute ethanol to precipitate the proteins. Beads were washed thrice with of 80% ethanol and proteins were eluted by two sequential washes of 0.2% SDS in PBS. The eluted proteins were directly transferred onto 30 µl of streptavidin-coated magnetic beads (prewashed with 0.2% SDS in PBS; New England Biolabs) and incubated on a shaker for 1 h at r.t. Streptavidin-beads were washed thrice with 8 M urea, PBS pH 7.4 and MilliQ water. Washed beads were resuspended in 100 mM ABC buffer, followed by reduction and alkylation (5 mM TCEP, 10 mM iodoacetamide, 37 °C, 30 min) and proteins were trypsinized overnight at 37 °C (1.5 µg/sample, Promega). The supernatant was removed, acidified to 1% TFA, centrifuged at 10‘000 rcf for 5 minutes and stored at -20 °C until measurement. Before MS/MS analysis a quality-control run was conducted on a Waters XEVO G2-XS QTof instrument to ensure complete digestion.

Samples were diluted based on the QC-runs to ensure equal loading. LC-MS/MS analysis was performed using an UltiMate 3000 RSLC nano LC system coupled on-line to an Orbitrap Fusion mass spectrometer (Thermo Fisher Scientific). For sample loading a PepMap C-18 trap-column (Thermo Fischer Scientific) of 0.075 mm ID x 50 mm length, 3 µm particle size and 100 Å pore size was used. The loading mobile phase A contained 1% acetonitrile and 0.05% TFA acid in water, and mobile phase B 0.05% TFA acid in acetonitrile. Reversed-phase separation was performed using a 50 cm analytical column (in-house packed with Poroshell 120 EC-C18, 2.7µm, Agilent Technologies) with mobile phase A contained 0.1% formic acid in water, and mobile phase B 0.1% formic acid in acetonitrile using a 93 minutes gradient (4-5%B 0-8 minutes; 5-25%B in 8-74 minutes; 25-28%B 74-80 minutes; 28-31%B 80-86 minutes; 31-36%B 86-92 minutes; 36-40%B 92-95 minutes; 40-50%B 95-96 minutes; 50-80%B 96-101 minutes; 80%B 101-104 minutes; 80-4%B 104-104.1 minutes). Data was acquired using survey scans in a range of 375 to 1500 m/z with a resolution of 120k and an AGC target value of 4e5. Precursor ions with charge states 2-4 were isolated with a mass selecting quadrupole (isolation window 1.6 m/z) with 40 sec dynamic exclusion (+/- 10 ppm). Precursor ions were fragmented using higher-energy collisional dissociation (HCD) applying a normalized collision energy (NCE) of 30. The maximum injection time was set to 22 ms to collect 5e4 precursor ions. Fragment ion spectra were acquired in the Orbitrap with a resolution of 15k (FWHM).

The obtained raw-data was analyzed using FragPipe (v20 to 21) using the built-in LFQ-MBR workflow using the whole human proteome as search space and applying the following MSFragger[11] settings: Precursor mass tolerance: +/-10 ppm; Fragment mass tolerance: +/-20 ppm; Mass calibration & parameter optimization enabled; Isotope error: 0/1/2; Enzyme: Trypsin (cuts after K & R, no cut before P), Peptide length: 7-50 AA; Peptide mass range: 500-5000 Da; Variable modifications: Oxidation (M, 15.9949 Da, up to 3x), Acetylation (N-term, 42.0106 Da); Carbamidomethylation of C was set as fixed modification (57.02146 Da). Validation was performed via Percolator & ProteinProphet and an FDR of 1% was applied. IonQuant[12] or MaxQuant were used for label free quantification, with MBR (FDR of 1%) and normalization across runs enabled. Analyzed files were further processed using FragPipe-Analyst[13] as follows: Proteins have to be quantified in >66% of the files for at least one condition and in >66% of the files globally. Variance stabilization was enabled, Perseus-type imputation was enabled for EGFR probes and Benjamin Hochberg type FDR correction was enabled. Unless stated differently the following significance cut offs were used: $\log_2$ fold-change >2.5; $-\log_{10}$ (adjusted) p-value >2.

4.9 Treatment and analysis for PROTAC mediated degradation

6x10^6 Ramos cells (in 2 mL RPMI 1640 +10% FCS) were treated with DMSO or BTK-PROTAC (**14**) at a final concentration of 625 nM (1% DMSO) for 24 h at 37 °C. Afterwards, the cells were collected by centrifugation (1000 rcf for 5 min.), and washed once with PBS. Next, cells were lysed using RIPA lysis buffer (200 uL, containing 50 mM *N*-ethylmaleimide, 1x protease inhibitor (cOmplete^{TM}, Roche) and 1:2000 benzoase) for 15 minutes at 37 °C. The protein amount was normalized and samples were denatured at 95 °C for 5 minutes in 1x Laemmli buffer. Proteins were seperated on precast 4-20% SDS-PAGE and transferred to a polyvinylidene difluoride (PVDF) membrane using a Trans-Blot Turbo (Bio-Rad) semi-dry blotting system according to the manufacturer's instructions. The PVDF membrane was blocked with 5% milk-powder in Tris-buffered saline containing Tween 20 (TBST) solution for 1 hour at room temperature, washed in TBST and probed with primary anti-BTK antibody (#56044s, Cell-Signaling; 1:1000 in TBST + 5% milk), for 1 h at roomtemperature or overnight at 4 °C. Following washes with TBST, the blots were incubated with a horseradish peroxidase (HRP)-conjugated goat polyclonal anti-mouse antibody (Dako Denmark, P0047) at a 1:10000 dilution. The HRP signal was detected using the Pierce ECL Western Blotting Substrate (Thermo Fisher Scientific) and imaged using the Gel Doc XR+ imaging system. After BTK imaging, the blot was stripped and the procedure was repeated using an anti-Tubulin antibody (T5168, Sigma Aldrich, 1:5000 in TBST + 5% milk). Lanes and bands were quantified in ImageLab 6.1 (BioRad) and plotted and analysis using GraphPad PRISM v5.04.

For proteomics analysis, protein concentration was determined by BCA (Pierce 23225) and lysates (1 mg/ml, 100 µg) were precipitated onto magnetic sp3-beads (1:1 hydrophilic/hydrophobic, 10 µl each) using 400 µl abs. EtOH. Beads were washed twice with 80% EtOH and resuspended in 100 µl 8 M Urea (100 mM Tris, pH 8.5). After reduction (10 mM TCEP, 37 °C, 30 min) and alkylation with iodoacetamide (20 mM, r.t., 45 min), samples were precipitated once more using 400 400 µl abs. EtOH. Beads were resuspended in 100 µl 8 M Urea (100 mM Tris, pH 8.5), diluted to 2 M Urea using PBS (10 mM, pH 7.4) and trypsinized overnight at 37 °C (1.5 µg/sample, Promega). Peptides were precipitated onto the magnetic beads by the addition of acetonitrile to a final concentration of 95%, followed by three washes with 100% acetonitrile. Tryptic peptides were eluted from the magnetic beads by the addition on 2% DMSO in MQ. Samples were diluted based on the QC-runs and analyzed as described above.

The obtained raw-data was analyzed using FragPipe (v 21) using the built-in LFQ-MBR workflow using the whole human proteome as search space and applying the following MSFragger[11] settings: Precursor mass tolerance: +/-10 ppm; Fragment mass tolerance: +/-20 ppm; Mass calibration & parameter optimization enabled; Isotope error: 0/1/2; Enzyme: Trypsin (cuts after K & R, no cut before P), Peptide length: 7-50 AA; Peptide mass range: 500-5000 Da; Variable modifications: Oxidation (M, 15.9949 Da, up to 3x), Acetylation (N-term, 42.0106 Da); Carbamidomethylation of C was set as fixed modification (57.02146 Da). Validation was performed via Percolator & ProteinProphet and an FDR of 1% was applied. MaxQuant was used for label free quantification, with MBR (FDR of 1%) and normalization across runs enabled. Analyzed files were further processed using FragPipe-Analyst[13] as follows: Proteins have to be quantified in >66% of the files for at least one condition and in >66% of the files globally. Variance stabilization was enabled and Benjamin Hochberg type FDR correction was enabled. The following significance cut offs were used: $\log_2$ fold-change >1; $-\log_{10}$ p-value >2.

5. Chemical Synthesis

5.1 Synthesis of Small-Molecule Electrophiles

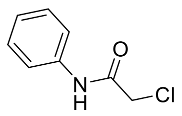

3

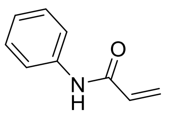

2

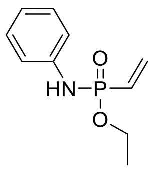

1

Compounds **1a-3a** were synthesized as reported in the literature.[14–16]

2-chloro-*N*-(4-ethynylphenyl)acetamide (3b):

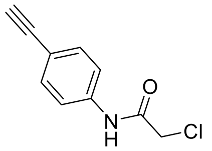

4-Ethynylaniline was dissolved in THF (0.5 M) and cooled to 0 °C with stirring. To this solution was added Et_3N (1 eq), followed by dropwise addition of chloroacetyl chloride (2 eq) in THF (0.5 M). The reaction mixture was allowed to warm to rt and stirred until completion. After removal of THF, the crude was partitioned between 1 M HCl and EtOAc and combined organic layers were washed with sat. aq. $NaHCO_3$, water, brine and dried over Na_2SO_4. After evaporation of the solvent, the product was obtained as a white solid.

^{1}H NMR (600 MHz, Chloroform-*d*) δ 8.30 (s, 1H), 7.56 (d, *J* = 8.6 Hz, 2H), 7.54 – 7.48 (m, 2H), 4.21 (s, 2H), 3.10 (s, 1H).
^{13}C NMR (151 MHz, Chloroform-*d*) δ 163.79, 137.06, 133.05, 119.64, 118.78, 83.07, 77.26, 42.85.
HRMS $C_{10}H_7ClNO$ $[M+H]^+$ calc.: 194.0367 Da; found: 194.0377 Da

N-(4-ethynylphenyl)acrylamide (2b):

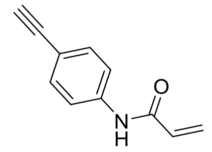

The compound was synthesized analogously to **1b** using acryloylchloride and obtained as a white solid.

^{1}H NMR (600 MHz, Chloroform-*d*) δ 7.72 (s, 1H), 7.59 (d, *J* = 8.2 Hz, 2H), 7.47 (d, *J* = 8.3 Hz, 2H), 6.45 (d, *J* = 16.8 Hz, 1H), 6.29 (dd, *J* = 16.8, 10.2 Hz, 1H), 5.79 (dd, *J* = 10.1, 1.2 Hz, 1H), 3.08 (s, 1H).
^{13}C NMR (151 MHz, Chloroform-*d*) δ 163.69, 138.18, 132.97, 130.92, 128.35, 119.65, 118.01, 83.31, 77.01.
HRMS $C_{11}H_9NO$ $[M+H]^+$ calc.: 172.0757 Da; found: 172.0765 Da

ethyl *N*-(4-ethynylphenyl)-P-vinylphosphonamidate (1b):

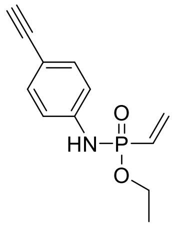

A flame-dried Schlenk flask was charged with 266 mg bis(diisopropylamino)-chloro phosphine (1.0 mmol, 1.0 eq.), dissolved in 1 ml of dry THF at -78 °C. 1.1 ml of vinyl magnesium bromide (1.0 M in THF, 1.10 mmol, 1.1eq.) were added and the reaction was stirred at room temperature for 30 minutes. Next, a solution of ethanol (1 mmol, 1 eq.) in 1 ml of MeCN and 2.45 ml tetrazole (0.45 M in MeCN, 1.1 mmol, 1.1 eq.) was added dropwise at -78 °C. The resulting suspension was heated to room temperature and stirred for 30 minutes. Afterwards, the solution was cooled to -78 °C again, and a new solution of the 4-ethynyl-phenylamine (1.2 mmol, 1.2 eq.) in 1 mL MeCN and 2.45 mL tetrazole (0.45 M in MeCN, 1.1 mmol, 1.1 eq.) was added. When full conversion was reached, 0.6 mL 30% v:v H_2O_2 solution was added and the product was purified by semi preparative HPLC. (35.5 mg, 15%).

^{1}H NMR (600 MHz, Chloroform-*d*) δ 7.42 – 7.36 (m, 2H), 7.05 – 6.90 (m, 2H), 6.53 (d, *J* = 5.6 Hz, 1H), 6.42 – 6.02 (m, 3H), 4.34 – 3.97 (m, 2H), 3.02 (s, 1H), 1.36 (t, *J* = 7.1 Hz, 3H).
^{31}P NMR (243 MHz, Chloroform-*d*) δ 15.25.
^{13}C NMR (151 MHz, Chloroform-*d*) δ 140.75, 135.09, 133.36 (2C), 127.04 (d, *J* = 173.1 Hz), 117.06 (d, *J* = 6.6 Hz, 2C), 114.92, 83.50, 76.17, 60.87 (d, *J* = 6.2 Hz), 16.19 (d, *J* = 6.7 Hz).
HRMS for $C_{12}H_{14}NO_2P$ $[M+H]^+$ calc.: 236.0835 Da; found: 236.0864 Da

5.2 Electrophilic "scout-fragments"

***N*-(3,5-bis(trifluoromethyl)phenyl)-2-chloroacetamide (3c):**

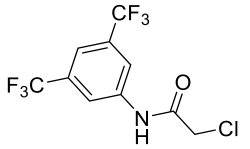

100 mg 3,5-bis(trifluoromethyl)aniline (0.43 mmol, 1 eq.) and 60 µl triethylamine were dissolved in 5 ml DCM and cooled to 0 °C followed by the dropwise addition of 35 µl chloroacetyl chloride (1.1 eq.). The reaction mixture was allowed to warm to r.t. and stirred until completion. The mixture was extracted with 1 M HCl, sat. aq. $NaHCO_3$, water, brine and dried over Na_2SO_4. After evaporation of the solvent, the product was obtained as a white solid (105 mg, 80% yield).

^{1}H NMR (600 MHz, Chloroform-*d*) δ 8.67 (s, 1H), 8.12 – 8.06 (m, 2H), 7.68 (s, 1H), 4.25 (s, 2H).
^{13}C NMR (151 MHz, Chloroform-*d*) δ 164.69, 138.14, 132.53 (q, *J* = 33.7 Hz), 122.90 (q, *J* = 272.9 Hz), 119.92 (d, *J* = 4.2 Hz), 118.55 (p, *J* = 3.9 Hz), 42.65.
HRMS $C_{10}H_6ClF_6NO$ $[M-H]^-$ calc.: 303.9969 Da; found: 303.9946 Da

***N*-(3,5-bis(trifluoromethyl)phenyl)acrylamide (2c):**

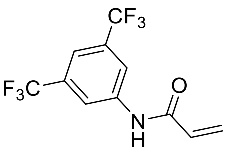

2c was synthesized according to Backus et al.[17]

Starting from 100 mg 3,5-bis(trifluoromethyl)aniline (0.43 mmol, 1 eq.) & 39 µl acryloyl chloride (1.1 eq.); 84 mg, 69% yield.

^{1}H NMR (600 MHz, Chloroform-*d*) δ 8.13 (s, 2H), 7.63 (s, 1H), 6.56 – 6.48 (m, 1H), 6.33 (dd, *J* = 16.9, 10.3 Hz, 1H), 5.92 – 5.87 (m, 1H).
^{13}C NMR (151 MHz, Chloroform-*d*) δ 164.13, 139.14, 132.42 (q, *J* = 33.5 Hz, 2C), 130.15, 129.55, 123.00 (q, *J* = 272.8 Hz, 2C), 119.76 (2C), 118.51 – 117.38 (m).
HRMS for $C_{11}H_7F_6NO$ $[M+H]^+$ calc.: 284.0505 Da; found: 284.0538 Da

ethyl N-(3,5-bis(trifluoromethyl)phenyl)-P-vinylphosphonamidate (1c):

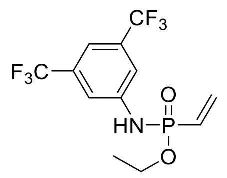

3c was synthesized according to the procedure published by Kasper et al.[16] and was obtained in 68% yield.

^{1}H NMR (600 MHz, Acetonitrile-d_3) δ 7.71 – 7.60 (m, 2H), 7.59 – 7.47 (m, 1H), 7.20 (d, J = 7.3 Hz, 1H), 6.43 – 6.07 (m, 3H), 4.27 – 4.04 (m, 2H), 1.33 (t, J = 7.1 Hz, 3H).
^{31}P NMR (243 MHz, Acetonitrile-d_3) δ 14.81.
^{13}C NMR (151 MHz, Acetonitrile-d_3) δ 143.13, 135.34, 131.86 (q, J = 33.0 Hz), 127.95, 126.83, 123.43 (q, J = 271.9 Hz), 114.20, 61.51 (d, J = 5.9 Hz), 15.52 (d, J = 6.5 Hz).
HRMS for $C_{12}H_{13}F_6NO_2P^+$ $[M+H]^+$ calc.: 348.0583 Da; found: 348.0571 Da

Synthesis of 4-amino-N-(2-(2-((7-chloroheptyl)oxy)ethoxy)ethyl)benzamide (S9)

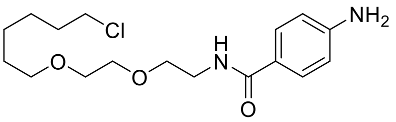

4-boc-amino-benzoic acid (91.2 mg, 384 µmol, 1 equiv.) and HATU (139 mg, 365 µmol, 1.9 equiv) were dissolved in DMF (4 mL) followed by addition of DIPEA (170 µL, 961 µmol, 5 equiv.) After stirring for 5 min 2-(2-((6-chlorohexyl)oxy)ethoxy)ethan-1-amine (50.0 mg, 192 µmol, 1 equiv.) was added. The reaction mixture was stirred over night at RT. The solvent was removed under reduced pressure (10^{-2} mbar, 50 °C) and the residue was picked up again in DCM/TFA for deprotection. After evaporating the solvent under a stream of N_2 the crude mixture was purified using prep-HPLC yielding **S9** (10.4 mg, 12%) as a yellow oil.

^{1}H NMR (600 MHz, Chloroform-d) δ 7.61 (d, J = 8.5 Hz, 2H), 6.65 (d, J = 8.6 Hz, 2H), 3.73 – 3.61 (m, 6H), 3.60 – 3.56 (m, 2H), 3.51 (t, J = 6.7 Hz, 2H), 3.45 (t, J = 6.7 Hz, 2H), 1.81 – 1.69 (m, 2H), 1.64 – 1.54 (m, 2H), 1.47 – 1.38 (m, 2H), 1.37 – 1.31 (m, 2H).
^{13}C NMR (151 MHz, Chloroform-d) δ 167.7, 149.6, 128.9, 124.2, 114.4, 71.4, 70.4, 70.1, 70.1, 45.2, 39.8, 32.6, 29.6, 26.8, 25.5.
HRMS for $C_{17}H_{27}ClN_2O_3$ $[M+H]^+$ calc.: 343.1788 Da; found: 343.1781 Da

Synthesis of 4-acrylamido-N-(2-(2-((7-chloroheptyl)oxy)ethoxy)ethyl)benzamide (S10)

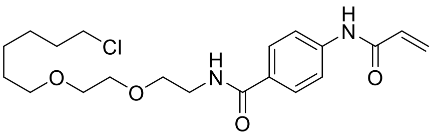

S9 (10 mg, 29.2 µmol, 1 equiv.) was dissolved in 2 mL of DCM and cooled to 0 °C, DIPEA (10.2 µL, 2 equiv) was added followed by acryloyl chloride (2.46 µL, 29.2 µmol, 1 equiv). The reaction mixture was stirred over night at RT. The solvent was then removed under reduced pressure. After purification via prep-HPLC **S10** (8.60 mg, 74% yield) was obtained as a white powder.

^{1}H NMR (600 MHz, Chloroform-d) δ 7.77 (d, J = 8.8 Hz, 2H), 7.67 (d, J = 8.6 Hz, 2H), 6.46 (dd, J = 16.9, 1.1 Hz, 1H), 6.28 (dd, J = 16.8, 10.3 Hz, 1H), 5.80 (dd, J = 10.2, 1.1 Hz, 1H), 3.66 (ddd, J = 9.3, 4.6, 1.9 Hz, 6H), 3.61 – 3.57 (m, 2H), 3.51 (t, J = 6.7 Hz, 2H), 3.46 (t, J = 6.7 Hz, 2H), 1.78 – 1.68 (m, 2H), 1.61 – 1.52 (m, 2H), 1.46 – 1.38 (m, 2H), 1.37 – 1.30 (m, 2H).
^{13}C NMR (151 MHz, Chloroform-d) δ 167.1, 163.8, 140.9, 131.0, 130.3, 128.6, 128.3, 119.5, 71.5, 70.4, 70.2, 69.9, 45.3, 39.9, 32.6, 29.6, 26.8, 25.5.
HRMS for $C_{20}H_{29}ClN_2O_4$ $[M+H]^+$ calc.: 397.1894 Da; found: 397.1910 Da

Synthesis of 4-azido-N-(2-(2-((6-chlorohexyl)oxy)ethoxy)ethyl)benzamide (S11)

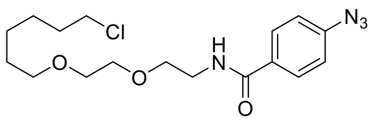

4-Azidobenzoic acid (81.6 mg, 0.50 mmol, 1 equiv.) and HATU (171 mg, 0.45 mmol, 0.9 equiv.) were dissolved in dry DMF (4 mL). DIPEA (0.261 mL, 1.50 mmol, 3 equiv.) was added at RT. After 5 min 2-(2-((6-chlorohexyl)oxy)ethoxy)ethan-1-amine (134 mg, 0.60 mmol, 3 equiv.) was added and the reaction mixture was stirred at RT for 16 h. The reaction mixture was then washed with destilled water (3 x 5 mL) extracted with cold Et_2O (3 x 5 mL) and dried over $MgSO_4$. The crude Product was obtained by removing the solvent under reduced pressure. Purification via Prep-HPLC (5-80% MeCN in H_2O + 0.1% TFA, 60 min) yielded **S11** (112 mg, 61%)

^{1}H NMR (600 MHz, Chloroform-d) δ 7.81 – 7.78 (m, 2H), 7.07 – 7.04 (m, 2H), 3.70 – 3.64 (m, 6H), 3.61 – 3.57 (m, 2H), 3.51 (t, *J* = 6.7 Hz, 2H), 3.45 (t, *J* = 6.7 Hz, 2H), 1.74 (dt, *J* = 14.6, 6.8 Hz, 2H), 1.57 (dt, *J* = 14.8, 6.8 Hz, 2H), 1.49 – 1.38 (m, 2H), 1.38 – 1.31 (m, 2H).
^{13}C NMR (151 MHz, Chloroform-d) δ 166.6, 143.4, 131.2, 129.0, 119.1, 71.4, 70.4, 70.2, 69.9, 45.1, 39.9, 32.6, 29.6, 26.8, 25.5.
HRMS for $C_{17}H_{25}ClN_4O_3$ $[M+H]^+$ calc.: 369.1693 Da; found: 369.1666 Da

Synthesis of ethyl N-(4-((2-(2-((6-chlorohexyl)oxy)ethoxy)ethyl)carbamoyl)phenyl)-P-vinylphosphonamidate (S12)

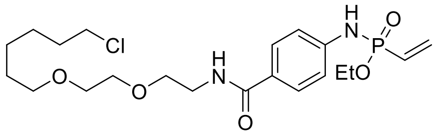

Diethylchlorophosphite (46.0 mg, 279 µmol, 1.0 equiv) was dissolved in 0.25 mL of dry THF under argon atmosphere and cooled to -78 °C. Afterwards, vinylmagnesium bromide (40.3 mg, 0.7 M in THF, 307 µmol, 1.1 equiv.) was added dropwise and the reaction was allowed to warm to room temperature. **S11** (103 mg, 279 µmol, 1.0 equiv) dissolved in DMF (1 mL) was added and stirred over 3 h. 5 mL of water were added and stirred for another 24 h. The solvents were removed under reduced pressure and the crude product was purified by Prep-HPLC (5-80% MeCN in H_2O + 0.1% TFA, 60 min) yielding **S12** (65 mg, 51%) as a white powder.

^{1}H NMR (600 MHz, Chloroform-d) δ 7.68 (d, *J* = 8.5 Hz, 1H), 7.00 (d, *J* = 8.6 Hz, 1H), 6.35 – 6.19 (m, 2H), 6.13 (ddd, *J* = 50.8, 12.2, 2.5 Hz, 1H), 4.22 (dp, *J* = 10.2, 7.2 Hz, 1H), 4.12 – 4.00 (m, 1H), 3.73 – 3.62 (m, 4H), 3.59 – 3.56 (m, 1H), 3.51 (t, *J* = 6.7 Hz, 1H), 3.46 (t, *J* = 6.6 Hz, 1H), 1.74 (dt, *J* = 14.7, 6.8 Hz, 2H), 1.58 (dt, *J* = 14.7, 6.8 Hz, 2H), 1.47 – 1.39 (m, 2H), 1.38 – 1.33 (m, 2H), 1.34 (t, *J* = 7.1 Hz, 3H).
^{31}P NMR (243 MHz, Chloroform-d) δ 15.16.
^{13}C NMR (151 MHz, Chloroform-d) δ 167.1, 143.4, 135.4, 128.7, 127.6, 126.5, 116.9 (d, *J* = 6.6 Hz), 71.4, 70.4, 70.2, 70.0, 61.2 (d, *J* = 6.2 Hz), 45.1, 39.8, 32.6, 29.6, 26.8, 25.5, 16.3 (d, *J* = 6.7 Hz).
HRMS for $C_{21}H_{34}ClN_2O_5P$ $[M+H]^+$ calc.: 461.1972 Da; found: 461.1954 Da

5.3 General procedure for phosphonite synthesis:

Diethyl-vinyl/ethyl-phosphonite:

Diethylchlorophosphite (1 mmol, 163 mg) was dissolved in 1 ml dry THF under argon atmosphere and cooled to -78 °C. Afterwards, 1.1 ml vinyl- or ethyl-magnesium bromide solution (1 M in THF) were added dropwise and the reaction was allowed to warm to room temperature. After quality control via ^{31}P-NMR (vinyl: 128-130 ppm), the crude phosphonite was used in the next step without further purification.

Di(but-3-yn-1-yl)-vinyl-phosphonite:

A flame-dried Schlenk flask was charged with 266 mg bis(diisopropylamino)-chloro phosphine (1.0 mmol, 1.0 eq.) and dissolved in 1 ml of dry THF at -78 °C. Vinyl magnesium bromide (1.0 M in THF, 1.10 mmol, 1.1eq.) was added and the reaction was stirred at room temperature for 30 minutes. Next, a solution of butin-1-ol (2.2 mmol, 2.2 eq.) in 5 ml tetrazole-solution (0.45 M in MeCN, 2.2 mmol, 2.2 eq.) was added dropwise at -78 °C. The resulting suspension was allowed to warm to room temperature and after quality control via ^{31}P-NMR (128-130 ppm), the crude phosphonite was used in the next step without further purification.

5.4 General Procedure for Staudinger-phosphonite reaction:

Aromatic azides were dissolved in dry DMF (0.1 M) and 2 eq. of the crude phosphonite (0.5 M in THF or ~0.125 M in THF/MeCN) were added dropwise. The reaction was stirred at 60°C until UPLC-MS analysis indicated full consumption of the starting material. The reaction mixture was diluted with H_2O and MeCN containing 0.1% TFA and purified via semi-preparative HPLC unless stated differently.

5.5 Synthesis of Phosphonamidate Analogues of Afatinib

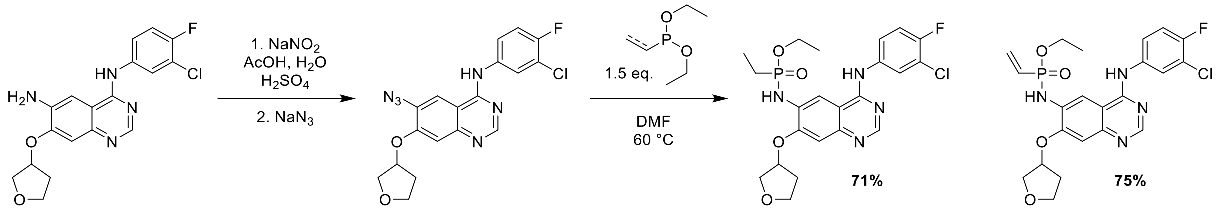

Scheme S1: Synthetic route towards vinyl- & ethyl-phosphonamidate analogues of Afatinib

Afatinib-N_3 (S2):

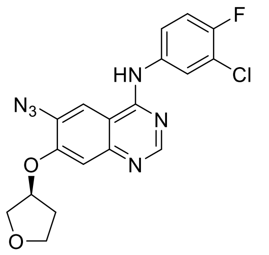

(S)-N4-(3-chloro-4-fluorophenyl)-7-((tetrahydrofuran-3-yl)oxy) quinazoline-4,6-diamine was dissolved in AcOH/MeOH (5:1) and cooled on ice. After 5 minutes, a solution of $NaNO_2$ (1.5 equiv., 2 M in H_2O) was added dropwise at 0 °C and stirred for 20 min followed by the slow addition of a NaN_3-solution (2.0 equiv., 2.5 M in H_2O) at 0 °C. The reaction mixture was then warmed to room temperature, basified with sat $NaHCO_3$ and immediately extracted with EtOAC and used in the next step without further purification.

^{1}H NMR (600 MHz, DMSO-*d*$_6$) δ 8.72 (s, 1H), 8.22 (s, 1H), 8.07 (dd, *J* = 6.8, 2.6 Hz, 1H), 7.73 (ddd, *J* = 8.9, 4.3, 2.6 Hz, 1H), 7.49 (t, *J* = 9.1 Hz, 1H), 7.30 (s, 1H), 5.34 (dt, *J* = 6.0, 2.9 Hz, 1H), 4.03 – 3.87 (m, 3H), 3.83 (td, *J* = 8.4, 4.5 Hz, 1H), 2.36 (dtd, *J* = 14.2, 8.2, 6.1 Hz, 1H), 2.16 – 2.05 (m, 1H). **^{13}C NMR** (151 MHz, DMSO-*d*$_6$) δ 157.76, 155.54, 153.91, 152.80, 144.15, 135.61, 130.14, 125.37, 124.13 (d, *J* = 7.2 Hz), 119.53 (d, *J* = 18.6 Hz), 117.26 (d, *J* = 21.9 Hz), 114.87, 108.79, 106.20, 80.22, 72.29, 66.97, 32.63.
HRMS for $C_{18}H_{15}ClFN_6O_2^+$ $[M+H]^+$ calc.: 401.0924 Da; found: 401.0920 Da

Afatinib-vinyl-phosphonamidate (4a):

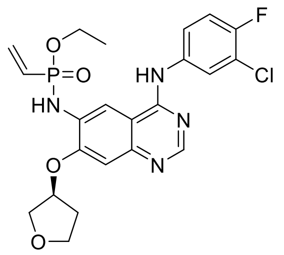

4a was synthesized according to general procedures 5.3 & 5.4 from 40 mg **S2** (0.1 mmol) and was obtained as a yellowish powder (37 mg, 75% yield).

^{1}H NMR (600 MHz, DMSO-*d*$_6$) δ 8.80 (s, 1H), 8.22 (d, *J* = 2.9 Hz, 1H), 7.91 (dd, *J* = 6.8, 2.6 Hz, 1H), 7.62 (ddd, *J* = 8.9, 4.3, 2.6 Hz, 1H), 7.54 (t, *J* = 9.0 Hz, 1H), 7.35 (d, *J* = 6.0 Hz, 2H), 6.48 (dddd, *J* = 23.6, 18.7, 12.6, 1.5 Hz, 1H), 6.34 – 6.08 (m, 2H), 5.35 – 5.25 (m, 1H), 4.18 – 4.05 (m, 3H), 4.00 (dd, *J* = 10.4, 4.7 Hz, 1H), 3.94 (q, *J* = 7.8 Hz, 1H), 3.83 (td, *J* = 8.3, 4.7 Hz, 1H), 2.41 – 2.29 (m, 1H), 2.28 – 2.15 (m, 1H), 1.30 (t, *J* = 7.0 Hz, 3H).
^{31}P NMR (243 MHz, DMSO-*d*$_6$) δ 16.23 (s, 1P), 16.17 (s, 1P).
^{13}C NMR (151 MHz, DMSO-*d*$_6$) δ 158.85, 155.58 (d, *J* = 246.1 Hz), 154.98 (d, *J* = 7.1 Hz), 149.94, 137.88, 134.91, 134.77, 133.24, 129.12 (d, *J* = 167.5 Hz), 127.46, 126.15 (d, *J* = 7.3 Hz), 119.69 (d, *J* = 18.5 Hz), 117.34 (d, *J* = 21.7 Hz), 111.71, 107.96, 102.03, 80.27, 72.22, 67.05, 61.11 (d, *J* = 5.8 Hz), 32.60, 16.65 (d, *J* = 6.4 Hz).
HRMS for $C_{22}H_{24}ClFN_4O_4P^+$ $[M+H]^+$ calc.: 493.1202 Da; found: 493.1233 Da

Afatinib-ethyl-phosphonamidate (4c):

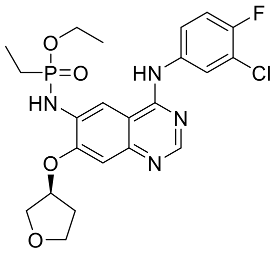

4c was synthesized according to general procedures 5.3 & 5.4 from 40 mg **S2** (0.1 mmol) and was obtained as a green-yellowish powder (35 mg, 71% yield)

^{1}H NMR (600 MHz, DMSO-d_6) δ 8.78 (s, 1H), 8.21 (d, *J* = 5.1 Hz, 1H), 7.91 (dd, *J* = 6.7, 2.5 Hz, 1H), 7.70 – 7.62 (m, 1H), 7.54 (t, *J* = 9.0 Hz, 1H), 7.34 (s, 1H), 7.04 (dd, *J* = 7.6, 1.9 Hz, 1H), 5.30 (dt, *J* = 6.3, 3.0 Hz, 1H), 4.16 – 3.99 (m, 4H), 3.94 (qd, *J* = 8.2, 2.3 Hz, 1H), 3.84 (td, *J* = 8.2, 4.6 Hz, 1H), 2.35 (dtdd, *J* = 14.3, 8.1, 6.2, 1.7 Hz, 1H), 2.20 (dq, *J* = 11.8, 5.4 Hz, 1H), 2.15 – 1.89 (m, 2H), 1.28 (t, *J* = 7.0 Hz, 3H), 1.09 (dtd, *J* = 20.2, 7.6, 2.8 Hz, 3H).
^{31}P NMR (243 MHz, DMSO-d_6) δ 32.55 (s, 1P), 32.46 (s, 1P).
^{13}C NMR (151 MHz, DMSO-d_6) δ 158.74, 156.32, 154.74 (d, *J* = 15.3 Hz), 150.02, 138.19, 135.02, 133.74, 127.35, 126.05 (d, *J* = 7.3 Hz), 119.68 (d, *J* = 18.8 Hz), 117.34 (d, *J* = 21.8 Hz), 111.34, 108.11, 102.29, 80.16, 72.27, 67.04, 60.55 (d, *J* = 6.6 Hz), 32.64, 20.62 (d, *J* = 130.0 Hz), 16.68 (d, *J* = 6.1 Hz), 6.70 (d, *J* = 5.7 Hz).
HRMS for $C_{22}H_{25}ClFN_4O_4P$ $[M+H]^+$ calc.: 495.1359 Da; found: 495.1355 Da

but-3-yn-1-yl N-(4-((3-chloro-4-fluorophenyl)amino)-7-(((S)-tetrahydrofuran-3-yl)oxy)quinazolin-6-yl)-P-vinylphosphonamidate (4b):

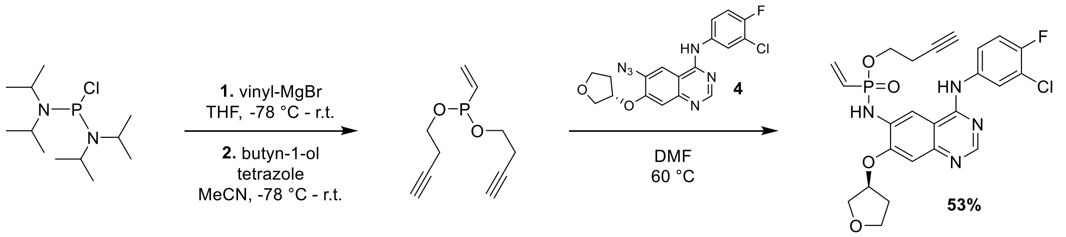

Scheme S4: Synthetic route towards a vinyl-phosphonamidate functionalized EGFR-probe based on the Afatinib drug-scaffold.

4b was synthesized according to general procedures 5.3 & 5.4 from 20 mg **S2** (0.05 mmol) and was obtained as a pale-yellow powder (13.7 mg, 53% yield)

^{1}H NMR (600 MHz, DMSO-d_6) δ 8.81 (s, 1H), 8.24 (d, *J* = 4.7 Hz, 1H), 7.92 (dd, *J* = 6.8, 2.6 Hz, 1H), 7.63 (ddd, *J* = 8.9, 4.3, 2.6 Hz, 1H), 7.55 (t, *J* = 9.0 Hz, 1H), 7.38 (dd, *J* = 7.7, 3.0 Hz, 1H), 7.33 (s, 1H), 6.50 (ddd, *J* = 23.8, 18.7, 12.6 Hz, 1H), 6.33 – 6.09 (m, 2H), 5.29 (dd, *J* = 6.3, 4.4 Hz, 1H), 4.20 – 4.05 (m, 3H), 4.00 (dd, *J* = 10.4, 4.7 Hz, 1H), 3.98 – 3.93 (m, 1H), 3.83 (td, *J* = 8.2, 4.6 Hz, 1H), 2.88 (td, *J* = 2.6, 1.0 Hz, 1H), 2.61 (td, *J* = 6.6, 2.7 Hz, 2H), 2.42 – 2.28 (m, 1H), 2.28 – 2.14 (m, 1H).
^{31}P NMR (243 MHz, DMSO-d_6) δ 16.66 (s, 1P), 16.59 (s, 1P).
^{13}C NMR (151 MHz, DMSO-d_6) δ 158.87, 156.42, 155.11 (d, *J* = 6.8 Hz), 154.79, 150.08, 135.12, 134.91, 132.94, 128.85 (d, *J* = 167.5 Hz), 127.55, 126.25, 119.68 (d, *J* = 18.6 Hz), 117.37 (d, *J* = 21.8 Hz), 112.36, 107.94, 102.09, 81.14, 80.27, 73.32, 72.23, 67.06, 62.88 (d, *J* = 5.6 Hz), 32.61, 20.64 (d, *J* = 6.8 Hz).
HRMS for $C_{24}H_{23}ClFN_4O_4P$ $[M+H]^+$ calc.: 517.1202 Da; found: 517.1195 Da

5.6 Synthesis of vinyl-phosphonamidate based EGFR-probes

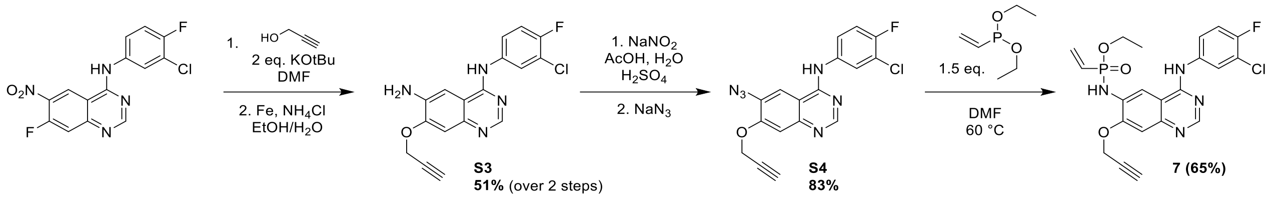

Scheme S5: Synthetic route towards the phosphonamidate based EGFR-Probe **7**

S3 was synthesized according to a published procedure.[18]

6-azido-N-(3-chloro-4-fluorophenyl)-7-(prop-2-yn-1-yloxy)quinazolin-4-amine (**S4**):

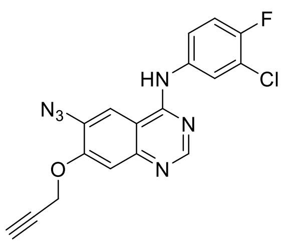

S4 was synthesized analogously to compound **S2** from **S3** and used in the next step without further purification.

^{1}H NMR (600 MHz, DMSO-*d*$_6$) δ 8.77 (s, 1H), 8.32 (s, 1H), 8.00 (d, *J* = 5.6 Hz, 1H), 7.69 (dt, *J* = 8.0, 3.4 Hz, 1H), 7.50 (t, *J* = 9.2 Hz, 1H), 7.43 (s, 1H), 5.11 (s, 2H), 2.51 (s, 1H).
^{13}C NMR (151 MHz, DMSO-*d*$_6$) δ 158.12, 155.30, 154.35, 152.13, 141.74, 134.96 (d, *J* = 3.3 Hz), 130.86, 126.08, 124.77 (d, *J* = 7.3 Hz), 119.68 (d, *J* = 18.7 Hz), 117.33 (d, *J* = 22.0 Hz), 114.86, 104.93, 80.41, 77.78, 57.73.
HRMS for $C_{17}H_{11}ClF_2N_6O^+$ $[M+H]^+$ calc.: 369.0661 Da, found: 369.0667 Da

ethyl N-(4-((3-chloro-4-fluorophenyl)amino)-7-(prop-2-yn-1-yloxy)quinazolin-6-yl)-P-vinyl-phosphonamidate (**7**):

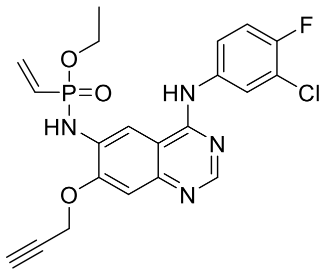

VPA **5** was synthesized according to general procedures 5.3 & 5.4 from 8 mg **S4** and was obtained as a pale-yellow powder (4.3 mg, 43% yield)

^{1}H NMR (600 MHz, DMSO-*d*$_6$) δ 9.73 (s, 1H), 8.46 (s, 1H), 8.03 (s, 1H), 8.02 (dd, *J* = 6.9, 2.6 Hz, 1H), 7.71 (ddd, *J* = 9.0, 4.3, 2.6 Hz, 1H), 7.42 (t, *J* = 9.1 Hz, 1H), 7.33 (d, *J* = 1.1 Hz, 1H), 6.48 – 6.37 (m, 1H), 6.30 – 6.01 (m, 2H), 5.07 (d, *J* = 2.4 Hz, 2H), 4.28 – 3.93 (m, 2H), 3.69 (t, *J* = 2.4 Hz, 1H), 1.25 (t, *J* = 7.0 Hz, 3H).
^{31}P NMR (243 MHz, DMSO-*d*$_6$) δ 16.09.
^{13}C NMR (151 MHz, Chloroform-*d*) δ 158.39, 157.15, 155.49, 152.98, 137.07, 136.38, 133.03, 132.61, 126.39, 124.08 (d, *J* = 7.0 Hz), 121.29 (d, *J* = 18.5 Hz), 116.74 (d, *J* = 22.2 Hz), 108.14, 107.25, 102.18, 78.10, 75.64, 62.75, 58.11, 16.37.
HRMS for $C_{21}H_{20}ClF_2N_4O_3P^+$ $[M+H]^+$ calc.: 461.0940 Da, found: 461.0892 Da

5.7 Synthesis of azide precursors for Ibrutinib analogues:

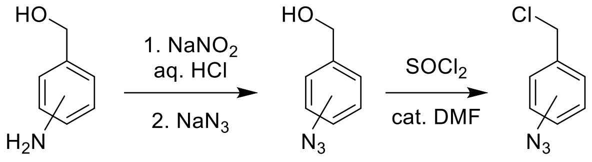

Scheme S6: Synthetic route towards benzyl chloride azides

Benzyl chloride azides were prepared according to a combination of procedures published by Muttach et al.[19] and Hong et al.[20]

5.8 General Procedure for aryl-azide synthesis[19]:

The aromatic amine (1.0 equiv.) was dissolved in a solution of H_2O and concentrated H_2SO_4 (6:1, 0.7 M) and cooled on ice. After 5 minutes, a solution of $NaNO_2$ (1.5 equiv., 2 M in H_2O) was added dropwise at 0 °C and stirred for 20 min followed by the slow addition of a NaN_3-solution (2.0 equiv., 2.5 M in H_2O) at 0 °C. The reaction mixture was then warmed to room temperature and stirred overnight. Products were obtained by extraction with EtOAC or Et_2O and used in the next step without further purification.

5.9 General Procedure for the synthesis of benzyl chlorides[20]:

To a 1 M solution of the corresponding benzyl-azide in DCM, $SOCl_2$ (2 eq.) was added dropwise at 0 °C. The reaction stirred at room temperature until full conversion was observed by TLC. In case the conversion was incomplete overnight, the reaction was heated to 50 °C. The reaction mixture was quenched with water and extracted with ether or EtOAc (3x). Combined organic fractions were washed subsequently with a saturated Na_2CO_3 solution and brine and dried over $MgSO_4$. After filtration and evaporation of the solvent, the obtained compounds were directly used in the next step without further purification.

1-azido-3,5-bis(trifluoromethyl)benzene:

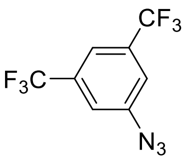

The title compound was synthesized according to the general procedure for aryl-azide synthesis from 100 mg 3,5-bis(trifluoromethyl)aniline (quant. yield).

^{1}H NMR (600 MHz, Chloroform-*d*) δ 7.67 (s, 1H), 7.46 (d, *J* = 1.8 Hz, 2H).
^{13}C NMR (151 MHz, Chloroform-*d*) δ 142.43, 133.41 (q, *J* = 33.9 Hz), 122.73 (q, *J* = 272.9 Hz), 119.12 (d, *J* = 3.9 Hz), 118.42 (p, *J* = 3.8 Hz).

N_3-1, (4-azidophenyl)methanol:

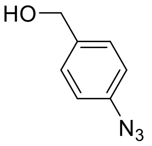

The title compound was synthesized according to the general procedure for aryl-azide synthesis from 1.15 g *p*-amino benzyl alcohol (9.34 mmol) and obtained in 91% yield (1.26 g).

^{1}H NMR (300 MHz, Chloroform-*d*) δ 7.44 – 7.27 (m, 2H), 7.11 – 6.92 (m, 2H), 4.65 (s, 2H).
^{13}C NMR (75 MHz, Chloroform-*d*) δ 139.33, 137.55, 128.54 (2C), 119.10 (2C), 64.61.

N_3-1a, 1-azido-4-(chloromethyl)benzene:

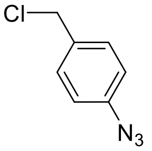

The title compound was synthesized according to the general procedure for benzyl-chloride synthesis from 1 g **N_3-1** (6.7 mmol) and obtained in 83% yield (930 mg).

^{1}H NMR (300 MHz, Chloroform-*d*) δ 7.50 – 7.32 (m, 2H), 7.16 – 6.93 (m, 2H), 4.59 (s, 2H).
^{13}C NMR (75 MHz, Chloroform-*d*) δ 140.22, 134.18, 130.19 (2C), 119.33 (2C), 45.71.

N_3-2, (3-azidophenyl)methanol:

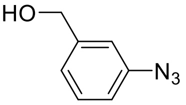

The title compound was synthesized according to the general procedure for aryl-azide synthesis from 1.15 g *m*-amino benzyl alcohol (9.34 mmol) and obtained in 85% yield (1.18 g).

^{1}H NMR (600 MHz, Chloroform-*d*) δ 7.36 (t, *J* = 7.8 Hz, 1H), 7.15 (d, *J* = 7.6 Hz, 1H), 7.08 (t, *J* = 1.9 Hz, 1H), 6.98 (dd, *J* = 7.9, 2.3 Hz, 1H), 4.72 (s, 2H).
^{13}C NMR (151 MHz, Chloroform-*d*) δ 142.88, 140.36, 129.92, 123.23, 118.21, 117.30, 64.74.

N_3-2a, 1-azido-3-(chloromethyl)benzene:

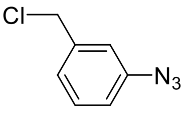

The title compound was synthesized according to the general procedure for benzyl-chloride synthesis from 1 g **N_3-2** (6.7 mmol) and obtained in ~50% conversion.

^{1}H NMR (600 MHz, Chloroform-*d*) δ 7.36 (td, *J* = 7.8, 4.4 Hz, 1H), 7.20 – 7.14 (m, 1H), 7.08 (d, *J* = 2.0 Hz, 1H), 7.03 – 6.96 (m, 1H), 4.458 (s, 1H).
^{13}C NMR (151 MHz, Chloroform-*d*) δ 142.87, 139.37, 129.92, 124.99, 118.99, 118.20, 45.49.

N_3-3, (2-azidophenyl)methanol:

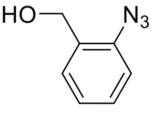

The title compound was synthesized according to the general procedure for aryl-azide synthesis from 1.15 g *o*-amino benzyl alcohol (9.34 mmol) and obtained in 89% yield (1.238 g).

^{1}H NMR (600 MHz, Chloroform-*d*) δ 7.52 – 7.32 (m, 2H), 7.25 – 7.11 (m, 2H), 4.66 (s, 2H).
^{13}C NMR (151 MHz, Chloroform-*d*) δ 137.85, 131.89, 129.24, 129.07, 124.95, 118.03, 61.61.

N_3-3a, 1-azido-2-(chloromethyl)benzene:

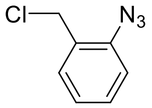

The title compound was synthesized according to the general procedure for benzyl-chloride synthesis from 1 g **N_3-3** (6.7 mmol) and obtained in 93% yield (1040 mg).

^{1}H NMR (600 MHz, Chloroform-*d*) δ 7.45 – 7.38 (m, 2H), 7.18 (ddd, *J* = 8.7, 7.4, 1.4 Hz, 2H), 4.61 (d, *J* = 1.0 Hz, 2H).
^{13}C NMR (151 MHz, Chloroform-*d*) δ 138.54, 131.03, 130.09, 128.62, 125.05, 118.42, 41.49.

N_3-5, 2-(2-azidophenyl)ethan-1-ol:

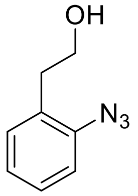

The title compound was synthesized according to the general procedure for aryl-azide synthesis from 1 g 2-(2-aminophenyl)ethan-1-ol and obtained in 99% yield (1.17 g).

^{1}H NMR (600 MHz, Chloroform-*d*) δ 7.31 (td, *J* = 7.7, 1.6 Hz, 1H), 7.24 (dd, *J* = 7.6, 1.5 Hz, 1H), 7.18 (dd, *J* = 8.0, 1.1 Hz, 1H), 7.12 (td, *J* = 7.4, 1.2 Hz, 1H), 3.85 (t, *J* = 6.6 Hz, 2H), 2.88 (t, *J* = 6.6 Hz, 2H).
^{13}C NMR (151 MHz, Chloroform-*d*) δ 138.39, 131.33, 129.96, 128.00, 124.81, 118.18, 62.62, 34.71.

N_3-5a, 2-azidophenethyl methanesulfonate:

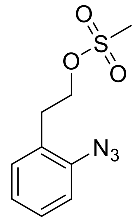

N_3-5 (500 mg) was dissolved in 10 ml DCM, 1.5 eq. TEA (0.64 ml, 4.6 mmol) were added and the mixture was cooled on ice. 0.26 ml methansulfonyl chloride (3.37 mmol, 1.1 eq.) were added dropwise. After complete addition, the mixture was allowed to warm to room temperature and stirred for 2 h. The reaction was quenched by the addition of 20 ml H_2O, followed by the addition of 20 ml 2 M HCl. The biphasic mixture was separated and the aqueous phase extracted 3x with DCM. Combined organic layers were dried over MgSO4, filtered and evaporated. The obtained compound was used without further purification. (672 mg, 91% yield)

^{1}H NMR (600 MHz, Chloroform-*d*) δ 7.27 (td, *J* = 7.7, 1.6 Hz, 1H), 7.19 (dd, *J* = 7.6, 1.6 Hz, 1H), 7.10 (dd, *J* = 8.0, 1.2 Hz, 1H), 7.06 (td, *J* = 7.4, 1.2 Hz, 1H), 4.33 (t, *J* = 7.0 Hz, 2H), 2.97 (t, *J* = 7.0 Hz, 2H), 2.83 (d, *J* = 1.2 Hz, 3H).
^{13}C NMR (151 MHz, Chloroform-*d*) δ 138.37, 131.48, 128.69, 127.53, 124.93, 118.24, 69.16, 37.05, 31.19.

5.10 Synthesis of phosphonamidate-analogues of Ibrutinib

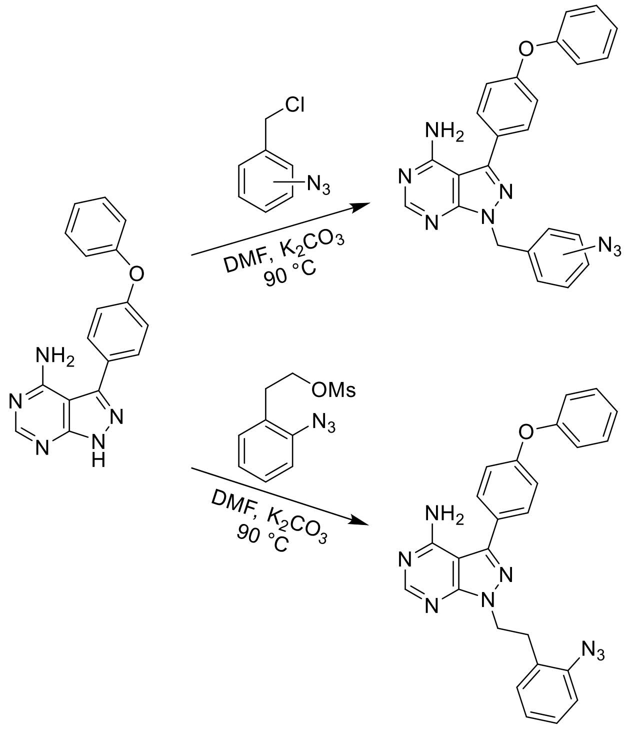

Figure S17: *Schematic representation of the synthetic route towards the azide precursors for phosphonamidate based BTK inhibitors/probes.*

Compounds **S5-S8** were synthesized according to the following procedure:

100 mg of Ibrutinib deacryloylpiperidine (0.3 mmol; BLDpharm, Reinbek Germany) was dissolved in 1 ml DMF and 2 eq. K_2CO_3 were added. The mixture was heated to 90 °C followed by the addition of 1.2 eq. of the corresponding benzylchloride or mesylate. After completion of the reaction (usually 2-4 h), the mixture was cooled to room temperature and extracted from 50 ml 1 M aq. HCl and ethyl acetate. Combined organic extracts were dried over $MgSO_4$, filtered and evaporated to dryness. The crude products were purified by chromatography over silica using a mixture of hexanes and ethyl acetate as eluent.

1-(2-azidobenzyl)-3-(4-phenoxyphenyl)-1H-pyrazolo[3,4-d]pyrimidin-4-amine:

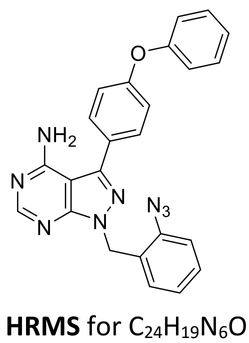

Eluent: Hex/EtOAc 3:1 → 1:3
83% yield

^{1}H NMR (600 MHz, Chloroform-*d*) δ 11.53 (s, 1H), 8.34 (s, 1H), 7.67 – 7.54 (m, 2H), 7.42 (dtd, *J* = 15.5, 7.6, 1.8 Hz, 3H), 7.26 – 7.08 (m, 8H), 6.35 (s, 1H), 5.65 (s, 2H).
^{13}C NMR (151 MHz, Chloroform-*d*) δ 159.79, 155.72, 153.47, 152.01, 147.22, 146.01, 138.26, 130.11 (2C), 129.95, 129.77 (2C), 125.94, 125.05, 124.54, 119.89 (2C), 119.20 (2C), 118.44, 96.90, 46.85.
HRMS for $C_{24}H_{19}N_6O$ $[M+H]^+$ calc.: 435.1676 Da; found: 435.1663 Da

1-(3-azidobenzyl)-3-(4-phenoxyphenyl)-1H-pyrazolo[3,4-d]pyrimidin-4-amine (S6):

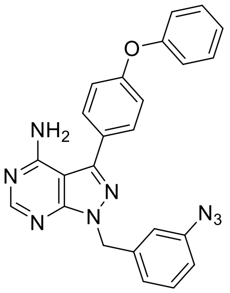

Eluent: Hex/EtOAc 3:1 → 1:3
70% yield

^{1}H NMR (600 MHz, Chloroform-*d*) δ 8.33 (s, 1H), 7.64 – 7.58 (m, 2H), 7.45 – 7.40 (m, 2H), 7.36 (t, *J* = 7.9 Hz, 1H), 7.22 (ddd, *J* = 7.5, 4.3, 2.9 Hz, 2H), 7.20 – 7.16 (m, 2H), 7.13 – 7.09 (m, 3H), 7.04 – 6.99 (m, 1H), 5.63 (s, 2H).
^{13}C NMR (151 MHz, Chloroform-*d*) δ 159.79, 155.70, 153.44, 151.78, 147.31, 146.20, 140.76, 136.85, 130.40, 130.11 (2C), 129.76 (2C), 124.98, 124.82, 124.55, 119.91 (2C), 119.19 (2C), 119.09, 119.00, 97.06, 51.23.
HRMS for $C_{24}H_{19}N_6O$ $[M+H]^+$ calc.: 435.1676 Da; found: 435.1663 Da

1-(4-azidobenzyl)-3-(4-phenoxyphenyl)-1H-pyrazolo[3,4-d]pyrimidin-4-amine (S7):

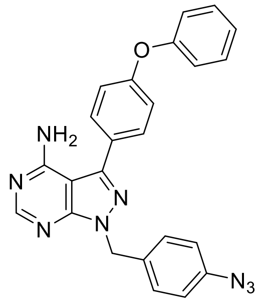

Eluent: Hex/EtOAc 3:1 → 1:3
87% yield

^{1}H NMR (300 MHz, Chloroform-*d*) δ 8.41 (s, 1H), 7.71 – 7.59 (m, 2H), 7.49 – 7.34 (m, 4H), 7.26 – 7.10 (m, 3H), 7.15 – 7.04 (m, 2H), 7.05 – 6.95 (m, 2H), 5.61 (s, 2H).
^{13}C NMR (75 MHz, Chloroform-*d*) δ 158.78, 156.97, 156.20, 154.16, 153.95, 144.76, 139.82, 132.93, 130.01 (2C), 129.92 (2C), 129.77 (2C), 127.17, 124.16, 119.61 (2C), 119.29 (2C), 119.13 (2C), 98.25, 50.38.
HRMS for $C_{24}H_{19}N_6O$ $[M+H]^+$ calc.: 435.1676 Da; found: 435.1663 Da

1-(2-azidophenethyl)-3-(4-phenoxyphenyl)-1H-pyrazolo[3,4-d]pyrimidin-4-amine (S8):

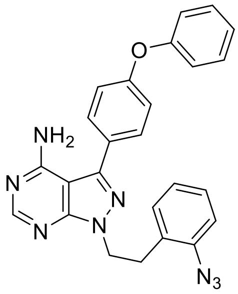

Eluent: Hex/EtOAc 3:1 → 1:2
80% yield

^{1}H NMR (600 MHz, Chloroform-*d*) δ 8.32 (s, 1H), 7.65 – 7.62 (m, 2H), 7.43 – 7.39 (m, 2H), 7.26 (td, *J* = 7.7, 1.6 Hz, 1H), 7.21 – 7.15 (m, 3H), 7.13 (dd, *J* = 8.0, 1.1 Hz, 1H), 7.12 – 7.09 (m, 2H), 7.07 (dd, *J* = 7.6, 1.5 Hz, 1H), 6.99 (td, *J* = 7.5, 1.1 Hz, 1H), 4.68 (dd, *J* = 8.0, 6.7 Hz, 2H), 3.25 (t, *J* = 7.3 Hz, 2H).
^{13}C NMR (151 MHz, Chloroform-*d*) δ 158.51, 157.61, 156.40, 155.05, 154.31, 143.88, 138.54, 131.01, 129.96 (2C), 129.93 (2C), 129.41, 128.22, 127.77, 124.68, 124.01, 119.51 (2C), 119.16 (2C), 118.10, 98.23, 47.01, 31.64.
HRMS for $C_{25}H_{21}N_6O$ $[M+H]^+$ calc.: 449.1833 Da; found: 449.1815 Da

Compounds **8-11** were synthesized according to the general procedure for Staudinger phosphonite reaction starting from the corresponding azide precursor and purified via semi-preparative HPLC.

but-3-yn-1-yl N-(2-(2-(4-amino-3-(4-phenoxyphenyl)-1H-pyrazolo[3,4-d]pyrimidin-1-yl)ethyl) phenyl)-P-vinylphosphonamidate (8):

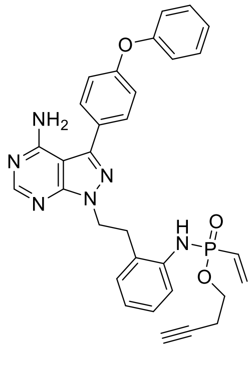

^{1}H NMR (600 MHz, DMSO-d_6) δ 8.40 (s, 1H), 7.70 – 7.61 (m, 2H), 7.52 – 7.42 (m, 2H), 7.25 (d, *J* = 8.0 Hz, 1H), 7.24 – 7.18 (m, 1H), 7.20 – 7.15 (m, 2H), 7.17 – 7.11 (m, 3H), 7.09 (td, *J* = 7.7, 1.7 Hz, 1H), 7.00 (dt, *J* = 7.6, 1.3 Hz, 1H), 6.85 (td, *J* = 7.4, 1.2 Hz, 1H), 6.33 (ddd, *J* = 23.4, 18.7, 12.5 Hz, 1H), 6.23 – 6.05 (m, 2H), 4.68 – 4.53 (m, 2H), 4.16 – 3.95 (m, 2H), 3.29 (t, *J* = 7.8 Hz, 2H), 2.87 (t, *J* = 2.6 Hz, 1H), 2.54 (td, *J* = 6.5, 2.7 Hz, 2H).
^{31}P NMR (243 MHz, DMSO-d_6) δ 16.72.
^{13}C NMR (151 MHz, DMSO-d_6) δ 158.02, 156.61, 155.29, 153.16, 151.32, 145.26, 138.67, 134.66, 130.62, 130.53, 129.94, 129.56 (d, *J* = 8.4 Hz), 128.84, 127.70, 127.26, 124.39, 123.06, 121.99, 119.56, 119.46, 97.25, 81.27, 73.13, 62.60 (d, *J* = 5.6 Hz), 47.03, 31.04, 20.67 (d, *J* = 7.0 Hz).
HRMS for $C_{31}H_{29}N_6O_3P$ $[M+H]^+$ calc.: 565.2112 Da; found: 565.2120 Da

but-3-yn-1-yl N-(2-((4-amino-3-(4-phenoxyphenyl)-1H-pyrazolo[3,4-d]pyrimidin-1-yl)methyl) phenyl)-P-vinylphosphonamidate (9):

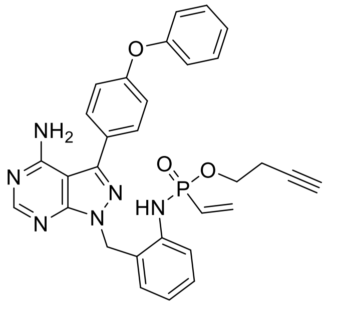

^{1}H NMR (600 MHz, DMSO-d_6) δ 8.42 (d, *J* = 2.9 Hz, 1H), 8.02 – 7.95 (m, 1H), 7.73 – 7.67 (m, 2H), 7.44 (dt, *J* = 7.8, 4.0 Hz, 2H), 7.17 (dddd, *J* = 29.8, 17.8, 10.0, 7.1 Hz, 6H), 6.98 (d, *J* = 8.4 Hz, 1H), 6.95 (s, 1H), 6.84 (d, *J* = 7.8 Hz, 1H), 6.32 – 5.98 (m, 3H), 5.51 (d, *J* = 2.9 Hz, 2H), 3.92 (ddd, *J* = 55.2, 12.8, 5.6 Hz, 2H), 2.89 – 2.86 (m, 1H), 2.48 (t, *J* = 7.4 Hz, 2H).
^{31}P NMR (243 MHz, DMSO-d_6) δ 15.12.
^{13}C NMR (151 MHz, DMSO-d_6) δ 157.94, 156.63 (2C), 153.69, 152.98, 145.24, 141.81, 137.96, 134.90, 130.62 (2C), 130.59 (2C), 129.71, 128.83 (d, *J* = 165.3 Hz), 127.48, 124.37, 120.41, 119.54 (2C), 119.45 (2C), 97.46, 81.09, 73.22, 62.45 (d, *J* = 5.6 Hz), 50.62, 20.54 (d, *J* = 7.1 Hz).
HRMS for $C_{30}H_{27}N_6O_3P$ $[M+H]^+$ calc.: 551.1955 Da; found: 551.1949 Da

but-3-yn-1-yl N-(3-((4-amino-3-(4-phenoxyphenyl)-1H-pyrazolo[3,4-d]pyrimidin-1-yl)methyl) phenyl)-P-vinylphosphonamidate (10):

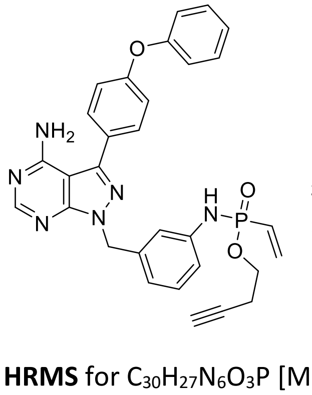

^{1}H NMR (600 MHz, DMSO-d_6) δ 8.41 (s, 1H), 7.79 – 7.65 (m, 3H), 7.54 – 7.41 (m, 2H), 7.33 (d, *J* = 8.0 Hz, 1H), 7.24 – 7.12 (m, 7H), 7.10 (d, *J* = 7.6 Hz, 1H), 6.95 (td, *J* = 7.5, 1.1 Hz, 1H), 6.31 (ddd, *J* = 23.7, 18.7, 12.3 Hz, 1H), 6.23 – 6.07 (m, 2H), 5.63 (s, 2H), 4.13 – 3.96 (m, 2H), 2.89 (t, *J* = 2.6 Hz, 1H), 2.55 (td, *J* = 6.6, 2.7 Hz, 2H).
^{31}P NMR (243 MHz, DMSO-d_6) δ 16.21.
^{13}C NMR (151 MHz, DMSO-d_6) δ 157.99, 156.61, 153.48 (d, *J* = 19.1 Hz), 153.40, 145.21, 138.75, 135.21, 130.62, 130.57, 130.32, 129.45 (d, *J* = 60.3 Hz), 128.56, 127.32, 124.38, 122.95, 121.41, 119.55, 119.45, 97.48, 81.21, 73.19, 62.84 (d, *J* = 5.3 Hz), 47.60, 20.67 (d, *J* = 6.9 Hz).
HRMS for $C_{30}H_{27}N_6O_3P$ $[M+H]^+$ calc.: 551.1955 Da; found: 551.1949 Da

but-3-yn-1-yl N-(4-((4-amino-3-(4-phenoxyphenyl)-1H-pyrazolo[3,4-d]pyrimidin-1-yl)methyl) phenyl)-P-vinylphosphonamidate (11):

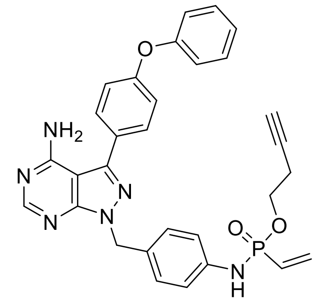

^{1}H NMR (600 MHz, DMSO-d_6) δ 8.45 (s, 1H), 7.98 (d, *J* = 8.1 Hz, 1H), 7.73 – 7.61 (m, 2H), 7.45 (t, *J* = 7.5 Hz, 2H), 7.21 (d, *J* = 6.5 Hz, 2H), 7.21 – 7.12 (m, 4H), 7.08 – 6.99 (m, 2H), 6.37 – 6.02 (m, 3H), 4.06 – 3.87 (m, 2H), 2.88 (q, *J* = 2.6, 2.1 Hz, 1H), 2.53 (td, *J* = 5.6, 3.0 Hz, 2H).
^{31}P NMR (243 MHz, DMSO-d_6) δ 15.38.
^{13}C NMR (151 MHz, DMSO-d_6) δ 157.97, 156.61 (2C), 153.23, 152.18 (d, *J* = 7.7 Hz), 145.37, 141.25, 134.96, 130.61 (4C), 129.30 (d, *J* = 59.8 Hz, 2C)), 129.29 (2C), 128.41, 127.33, 124.37, 119.54 (2C), 119.45 (2C), 117.96 (d, *J* = 6.8 Hz), 97.44, 81.18, 73.20, 62.49 (d, *J* = 5.3 Hz), 50.31, 20.60 (d, *J* = 7.0 Hz).
HRMS for $C_{30}H_{27}N_6O_3P$ $[M+H]^+$ calc.: 551.1955 Da; found: 551.1949 Da

ethyl N-(2-((4-amino-3-(4-phenoxyphenyl)-1H-pyrazolo[3,4-d]pyrimidin-1-yl)methyl)phenyl)-P-vinylphosphonamidate (9a):

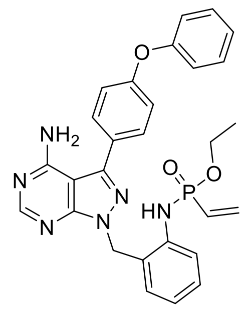

^{1}H NMR (600 MHz, Acetonitrile-d_3) δ 8.44 – 8.32 (m, 1H), 7.72 – 7.63 (m, 2H), 7.46 (dddd, *J* = 9.4, 7.3, 3.5, 1.9 Hz, 3H), 7.39 (dd, *J* = 8.1, 3.0 Hz, 1H), 7.32 – 7.17 (m, 4H), 7.17 – 7.11 (m, 2H), 7.09 – 7.00 (m, 2H), 6.36 – 6.01 (m, 3H), 5.70 – 5.60 (m, 2H), 4.21 – 3.98 (m, 2H), 1.28 (td, *J* = 7.1, 3.3 Hz, 3H).
^{31}P NMR (243 MHz, Acetonitrile-d_3) δ 15.03.
^{13}C NMR (151 MHz, Acetonitrile-d_3) δ 158.92, 156.34, 154.08, 151.70, 147.57, 146.77, 139.40, 134.61, 131.62, 130.13 (2C), 130.08 (2C), 129.81, 128.49 (d, *J* = 167.0 Hz), 125.77, 125.05 (d, *J* = 8.7 Hz), 124.16, 122.43, 120.13, 119.42 (2C), 119.33 (2C), 97.38, 61.04 (d, *J* = 5.9 Hz), 48.41, 15.72 (d, *J* = 6.4 Hz).
HRMS for $C_{28}H_{27}N_6O_3P$ $[M+H]^+$ calc.: 527.1955 Da; found: 527.1979 Da

ethyl N-(2-((4-amino-3-(4-phenoxyphenyl)-1H-pyrazolo[3,4-d]pyrimidin-1-yl)methyl)phenyl)-P-ethylphosphonamidate (9b):

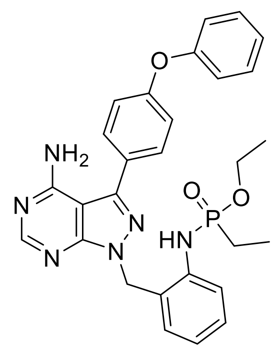

^{1}H NMR (600 MHz, DMSO-d_6) δ 8.39 (s, 1H), 7.67 (d, *J* = 8.4 Hz, 2H), 7.44 (t, *J* = 7.7 Hz, 2H), 7.37 – 7.32 (m, 2H), 7.25 – 7.18 (m, 2H), 7.16 (d, *J* = 8.4 Hz, 2H), 7.12 (d, *J* = 8.0 Hz, 3H), 6.93 (t, *J* = 7.5 Hz, 1H), 5.61 (s, 2H), 4.13 – 3.92 (m, 2H), 1.80 (dq, *J* = 15.6, 7.7 Hz, 2H), 1.22 (t, *J* = 7.1 Hz, 3H), 0.98 (dt, *J* = 19.8, 7.6 Hz, 3H).
^{31}P NMR (243 MHz, DMSO-d_6) δ 31.67.
^{13}C NMR (151 MHz, DMSO-d_6) δ 157.96, 156.62, 153.81, 153.63, 145.08, 139.57, 130.62, 130.55, 129.36, 127.39, 126.75 (d, *J* = 8.2 Hz), 124.38, 122.42, 120.65, 119.52, 119.45, 117.32, 97.48, 60.38 (d, *J* = 6.5 Hz), 47.68, 20.22 (d, *J* = 128.0 Hz), 16.70 (d, *J* = 6.2 Hz), 6.75 (d, *J* = 6.0 Hz).
HRMS for $C_{28}H_{29}N_6O_3P$ $[M+H]^+$ calc.: 529.2119 Da; found: 529.3519 Da

Ibrutinib-oVPA-Fluoresceine (13):

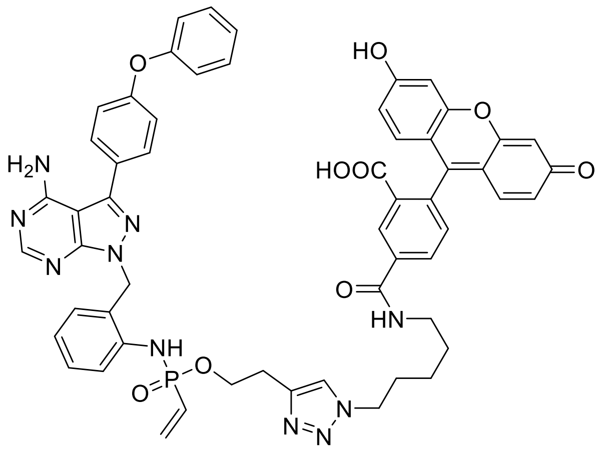

13 was synthesized from 2 mg **9** and Fluorescein-N_3. Starting materials were dissolved in 300 µl of a DMSO/PBS mixture (pH 7.4) and the reaction was initiated by the addition of 50 µl CuBr saturated in MeCN. Upon completion (as monitored by UPLC-MS) the compound was purified via semi-preparative HPLC and obtained as a yellow powder (3.1 mg, 82%)

HRMS for $C_{56}H_{49}N_{10}O_9P$ $[M+H]^+$ calc.: 1037.3494 Da; found: 1037.3469 Da
$[M+2H]^{2+}$ calc.: 519.1784 Da; found: 519.1777 Da

Ibrutinib-oVPA-Pomalidomide (14):

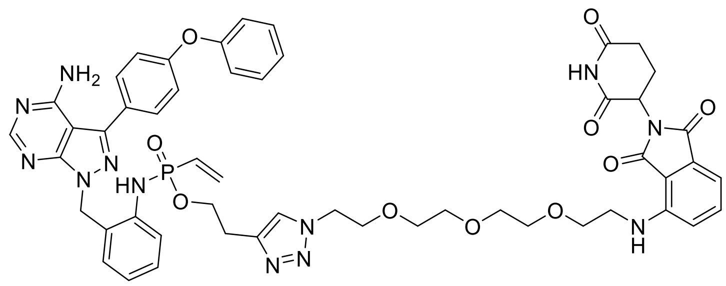

14 was synthesized from 2 mg **9** and Pomalidomide-PEG$_4$-N_3. Starting materials were dissolved in 300 µl of a DMSO/PBS mixture (pH 7.4) and the reaction was initiated by the addition of 50 µl CuBr saturated in MeCN. Upon completion (as monitored by UPLC-MS) the compound was purified via semi-preparative HPLC and obtained as a yellow powder (5.4 mg, 91%)

HRMS for $C_{51}H_{53}N_{12}O_{10}P$ $[M+H]^+$ calc.: 1025.3818 Da; found: 1025.3917 Da
$[M+2H]^{2+}$ calc.: 512.1945 Da; found: 512.1930 Da

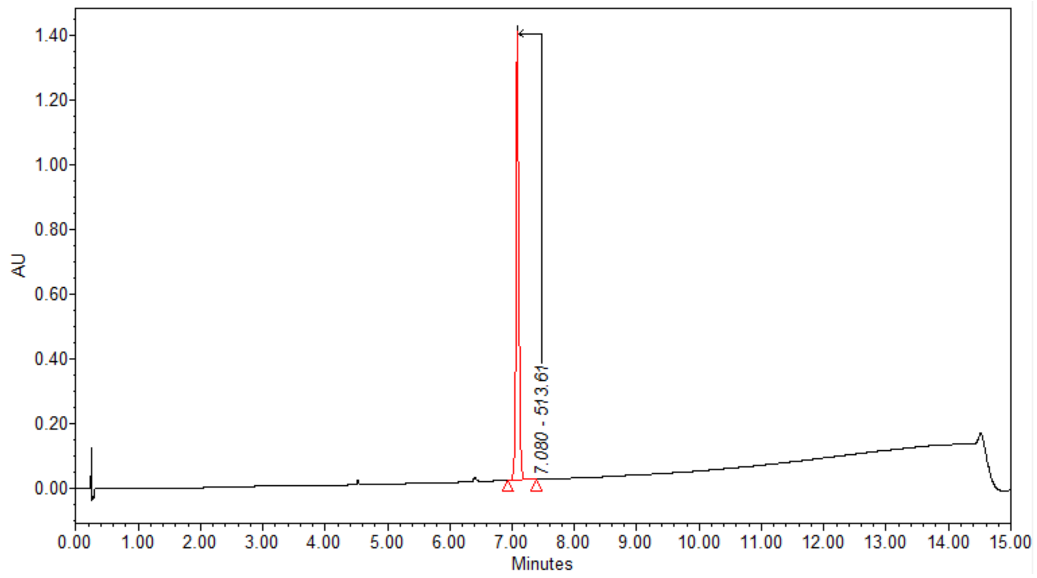

6. Supporting References

1. Shults, M. D., Janes, K. A., Lauffenburger, D. A. & Imperiali, B. A multiplexed homogeneous fluorescence-based assay for protein kinase activity in cell lysates. *Nature Methods 2005 2:4* **2**, 277–284 (2005).
2. Jones, G., Willett, P., Glen, R. C., Leach, A. R. & Taylor, R. Development and validation of a genetic algorithm for flexible docking. *J Mol Biol* **267**, 727–748 (1997).
3. Korb, O., Stützle, T. & Exner, T. E. Empirical scoring functions for advanced Protein-Ligand docking with PLANTS. *J Chem Inf Model* **49**, 84–96 (2009).
4. Solca, F. *et al.* Target binding properties and cellular activity of afatinib (BIBW 2992), an irreversible ErbB family blocker. *Journal of Pharmacology and Experimental Therapeutics* **343**, 342–350 (2012).
5. Bender, A. T. *et al.* Ability of Bruton's Tyrosine Kinase Inhibitors to Sequester Y551 and Prevent Phosphorylation Determines Potency for Inhibition of Fc Receptor but not B-Cell Receptor Signaling. *Mol Pharmacol* **91**, 208–219 (2017).
6. Peraro, L. *et al.* Cell Penetration Profiling Using the Chloroalkane Penetration Assay. *J Am Chem Soc* **140**, 11360–11369 (2018).
7. Crocker, L. B. *et al.* Energy-transfer photoproximity labelling in live cells using an organic cofactor. *Nature Chemistry 2025 17:12* **17**, 1928–1940 (2025).
8. Zanon, P. R. A., Lewald, L. & Hacker, S. M. Isotopically Labeled Desthiobiotin Azide (isoDTB) Tags Enable Global Profiling of the Bacterial Cysteinome. *Angewandte Chemie International Edition* **59**, 2829–2836 (2020).
9. Yan, T. *et al.* SP3-FAIMS Chemoproteomics for High-Coverage Profiling of the Human Cysteinome**. *ChemBioChem* **22**, 1841–1851 (2021).
10. Becker, T. *et al.* Transforming Chemical Proteomics Enrichment into a High-Throughput Method Using an SP2E Workflow. *JACS Au* **2**, 1712–1723 (2022).
11. Kong, A. T., Leprevost, F. V., Avtonomov, D. M., Mellacheruvu, D. & Nesvizhskii, A. I. MSFragger: Ultrafast and comprehensive peptide identification in mass spectrometry-based proteomics. *Nat Methods* **14**, 513–520 (2017).
12. Yu, F., Haynes, S. E. & Nesvizhskii, A. I. IonQuant enables accurate and sensitive label-free quantification with FDR-controlled match-between-runs. *Molecular and Cellular Proteomics* **20**, 100077 (2021).
13. Hsiao, Y. *et al.* Analysis and visualization of quantitative proteomics data using FragPipe-Analyst. *bioRxiv* 2024.03.05.583643 (2024) doi:10.1101/2024.03.05.583643.
14. Negmeldin, A. T., Knoff, J. R. & Pflum, M. K. H. The structural requirements of histone deacetylase inhibitors: C4-modified SAHA analogs display dual HDAC6/HDAC8 selectivity. *Eur J Med Chem* **143**, 1790–1806 (2018).
15. Oku, N., Murakami, M. & Miura, T. Photoassisted Cross-Coupling Reaction of α-Chlorocarbonyl Compounds with Arylboronic Acids. *Org Lett* **24**, 1616–1619 (2022).

16. Kasper, M. A. *et al.* Vinylphosphonites for Staudinger-induced chemoselective peptide cyclization and functionalization. *Chem Sci* **10**, 6322–6329 (2019).

17. Backus, K. M. *et al.* Proteome-wide covalent ligand discovery in native biological systems. *Nature* **534**, 570–574 (2016).

18. Shindo, N. *et al.* Selective and reversible modification of kinase cysteines with chlorofluoroacetamides. *Nat Chem Biol* **15**, 250–258 (2019).

19. Muttach, F., Mäsing, F., Studer, A. & Rentmeister, A. New AdoMet Analogues as Tools for Enzymatic Transfer of Photo-Cross-Linkers and Capturing RNA–Protein Interactions. *Chemistry – A European Journal* **23**, 5988–5993 (2017).

20. Hong, L., Lin, W., Zhang, F., Liu, R. & Zhou, X. Ln[N(SiMe3)2]3-catalyzed cycloaddition of terminal alkynes to azides leading to 1,5-disubstituted 1,2,3-triazoles: New mechanistic features. *Chemical Communications* **49**, 5589–5591 (2013).
