# Supplementary material for: Modular Vinyl Phosphonamidates for Cysteine-Directed Protein Targeting
Source: J Am Chem Soc. 2026 Feb 10;148(7):7772–81. doi: 10.1021/jacs.5c22349 (PMC12951430; doi:10.1021/jacs.5c22349)

NMR Spectra:

2-chloro-*N*-(4-ethynylphenyl)acetamide (3b):

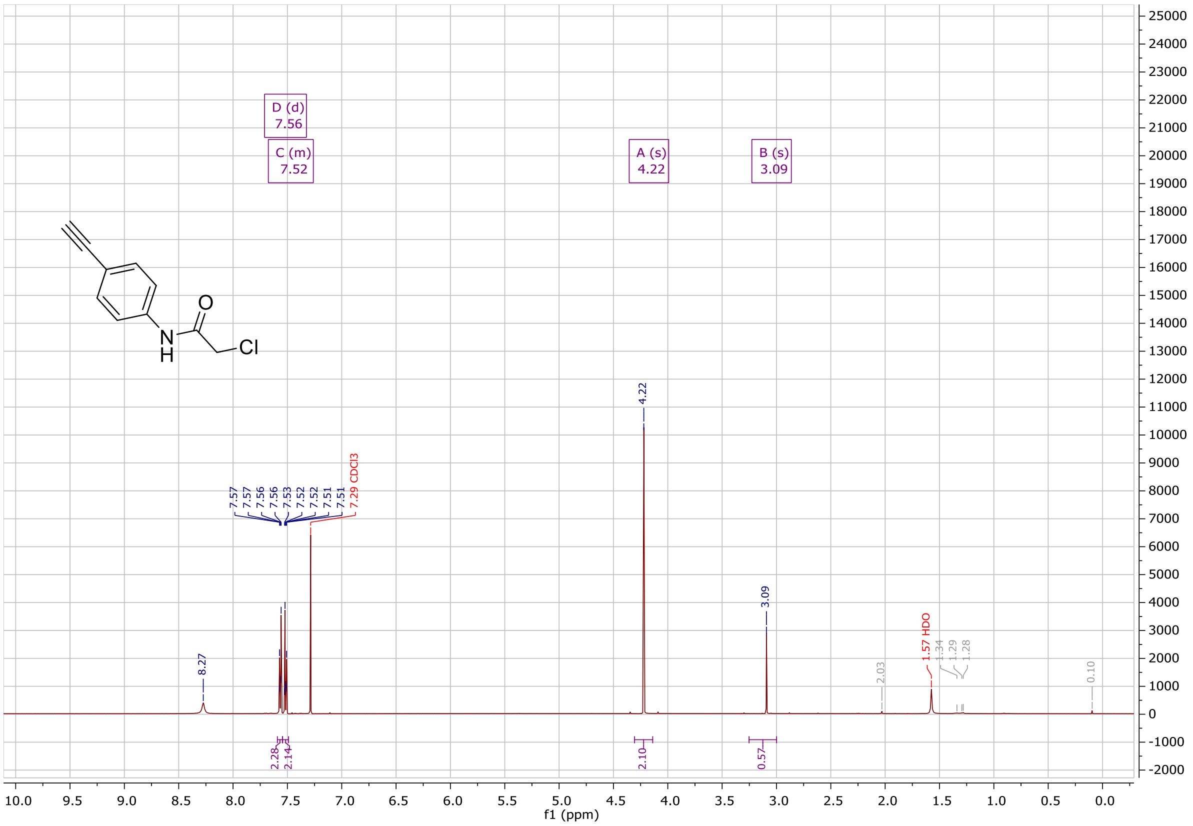

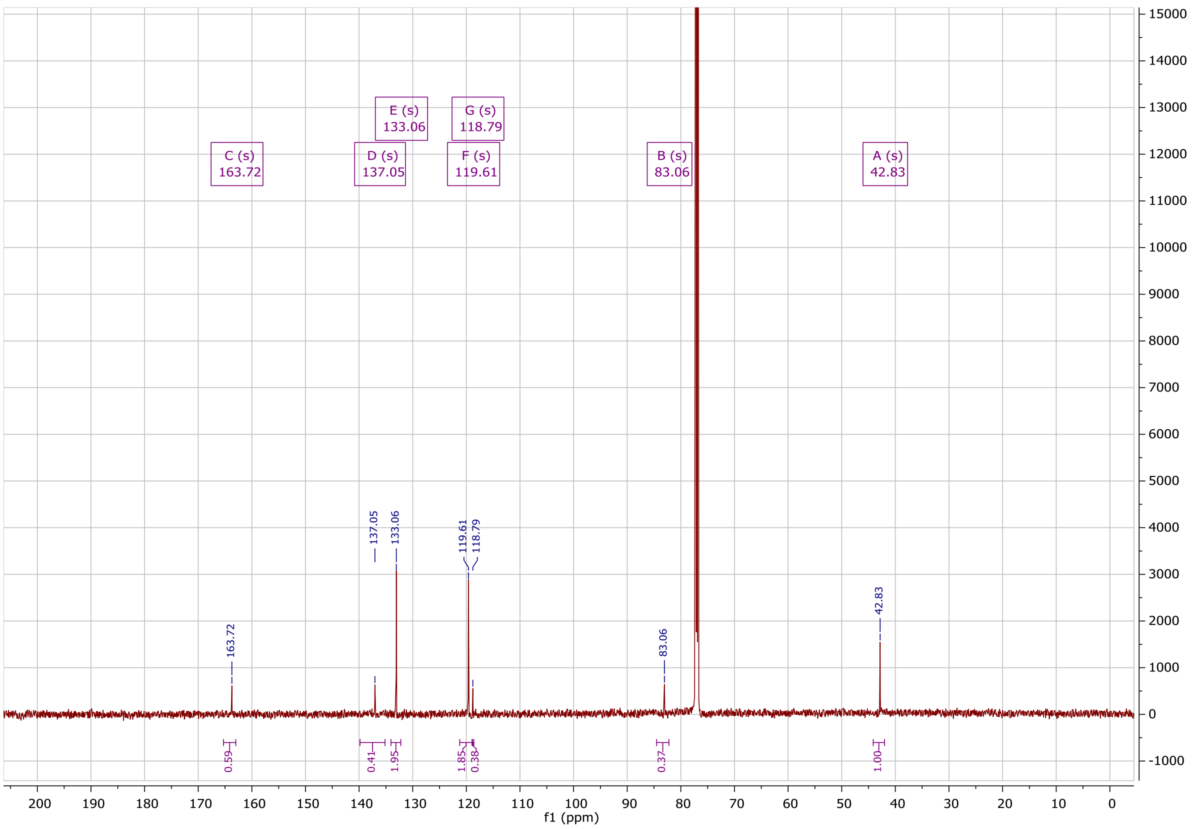

N-(4-ethynylphenyl)acrylamide (2b):

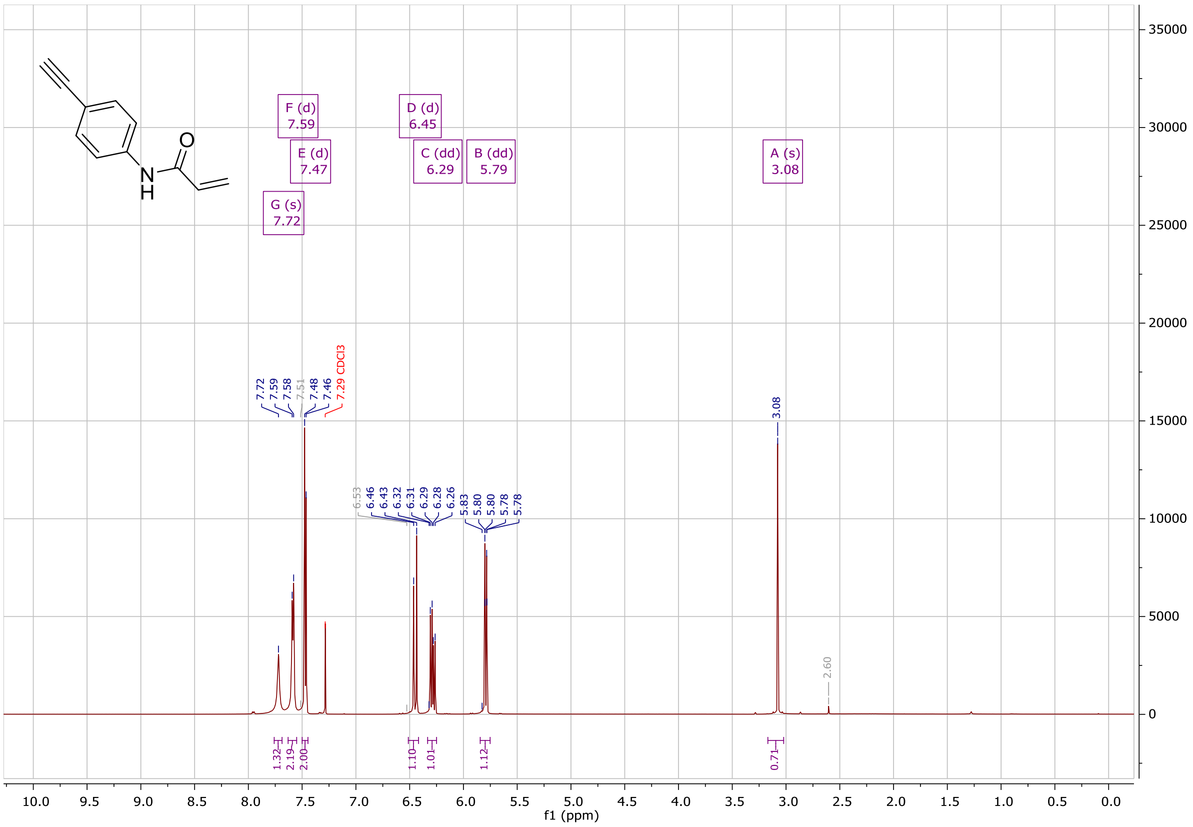

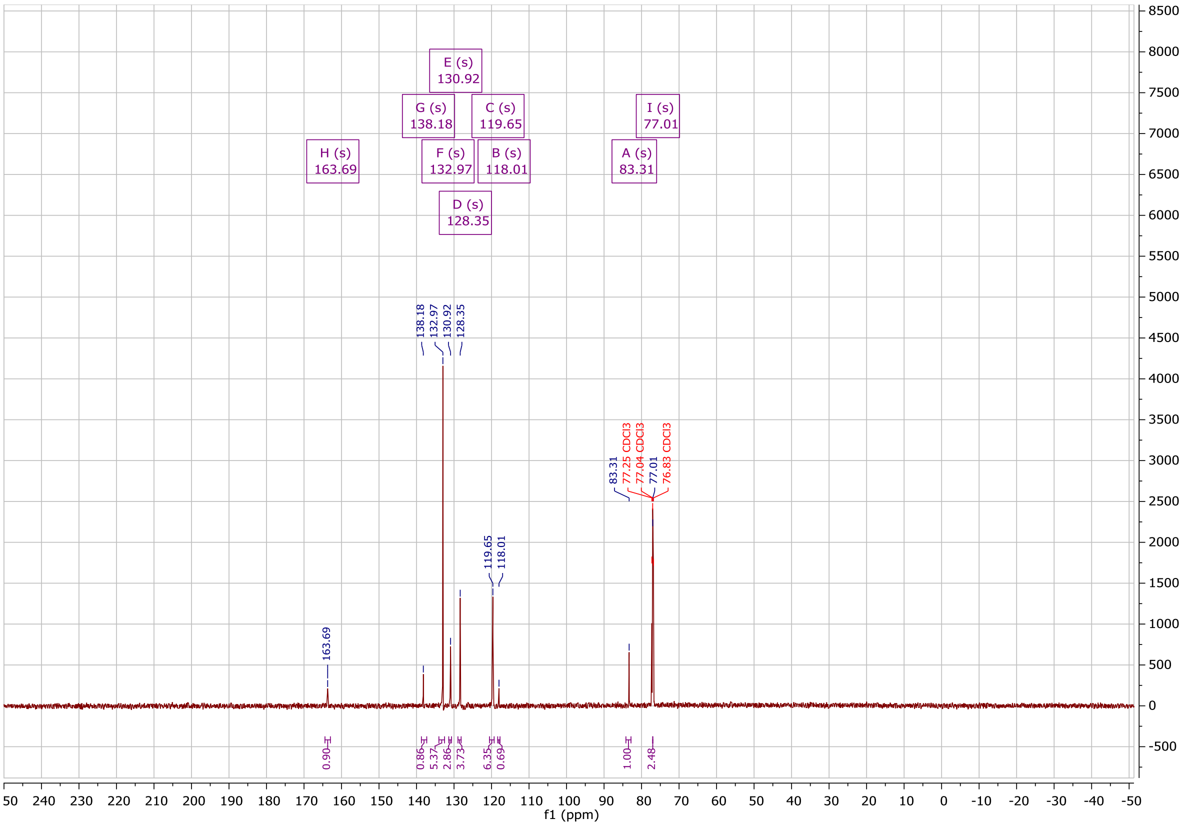

ethyl N-(4-ethynylphenyl)-P-vinylphosphonamidate:

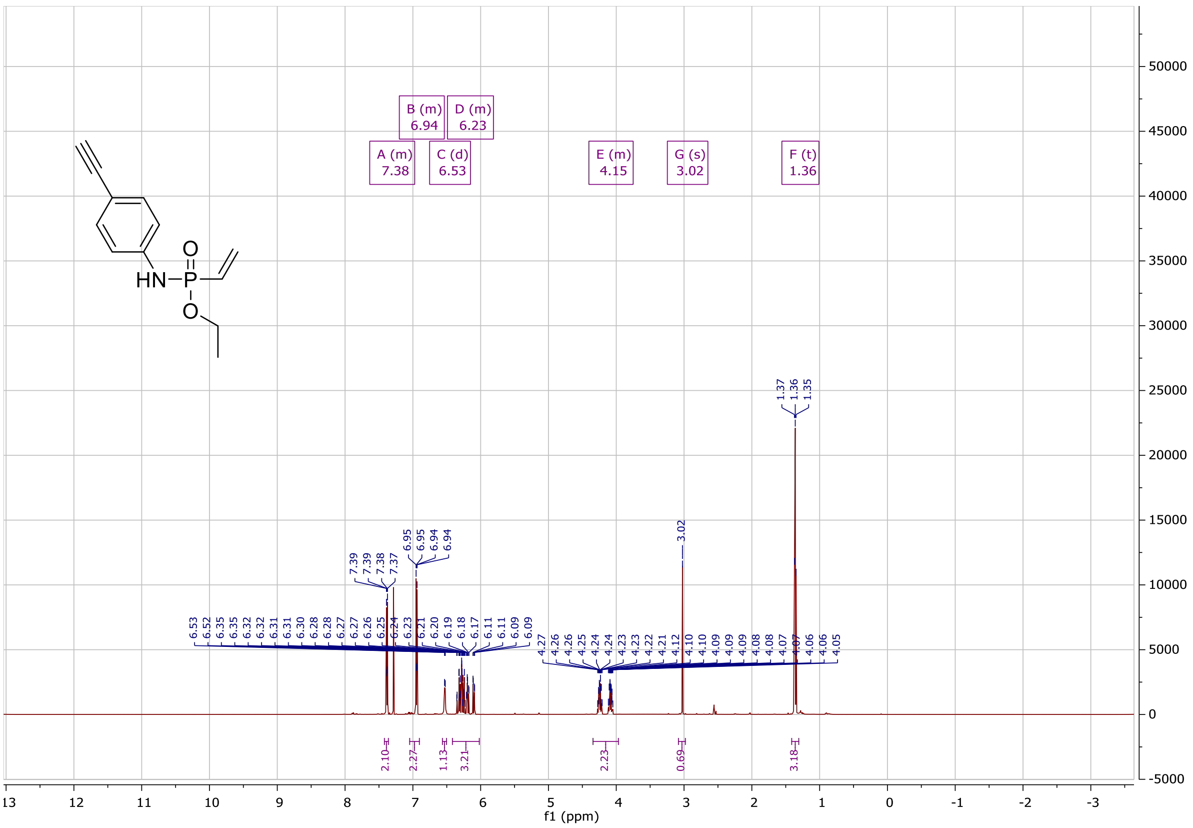

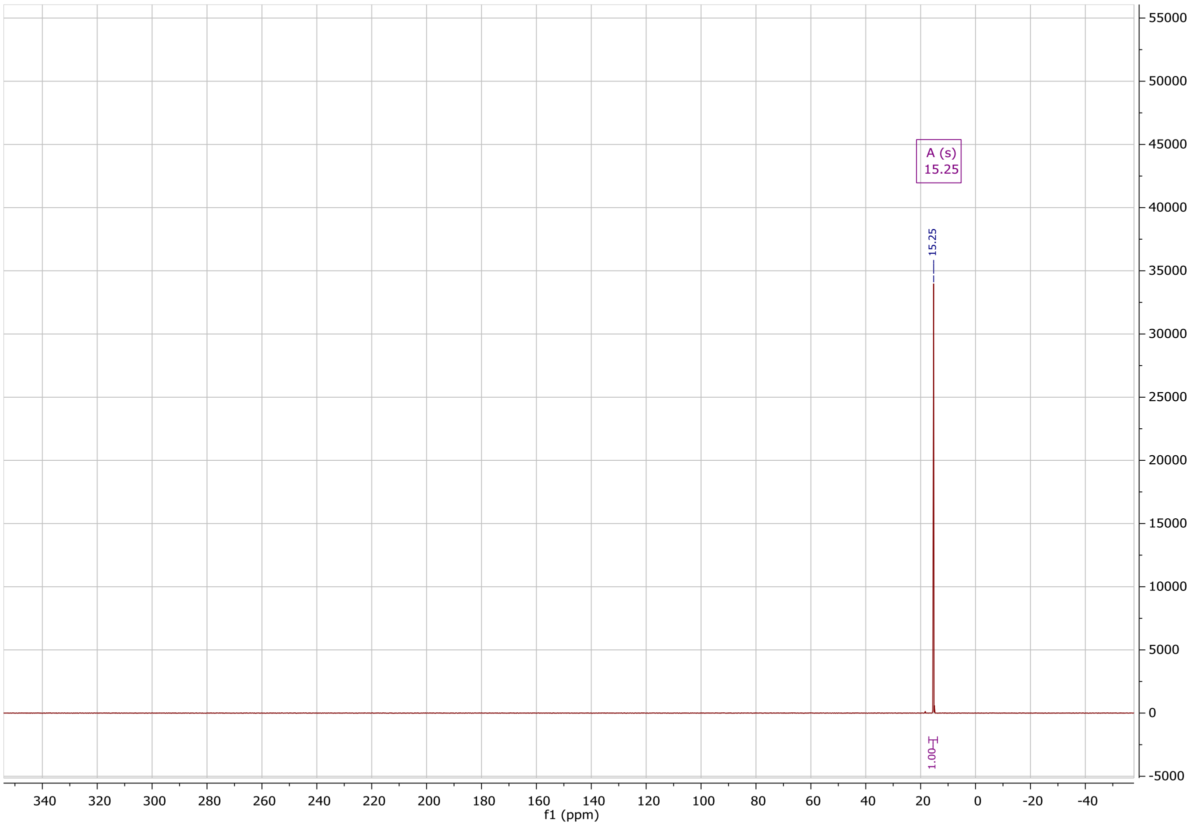

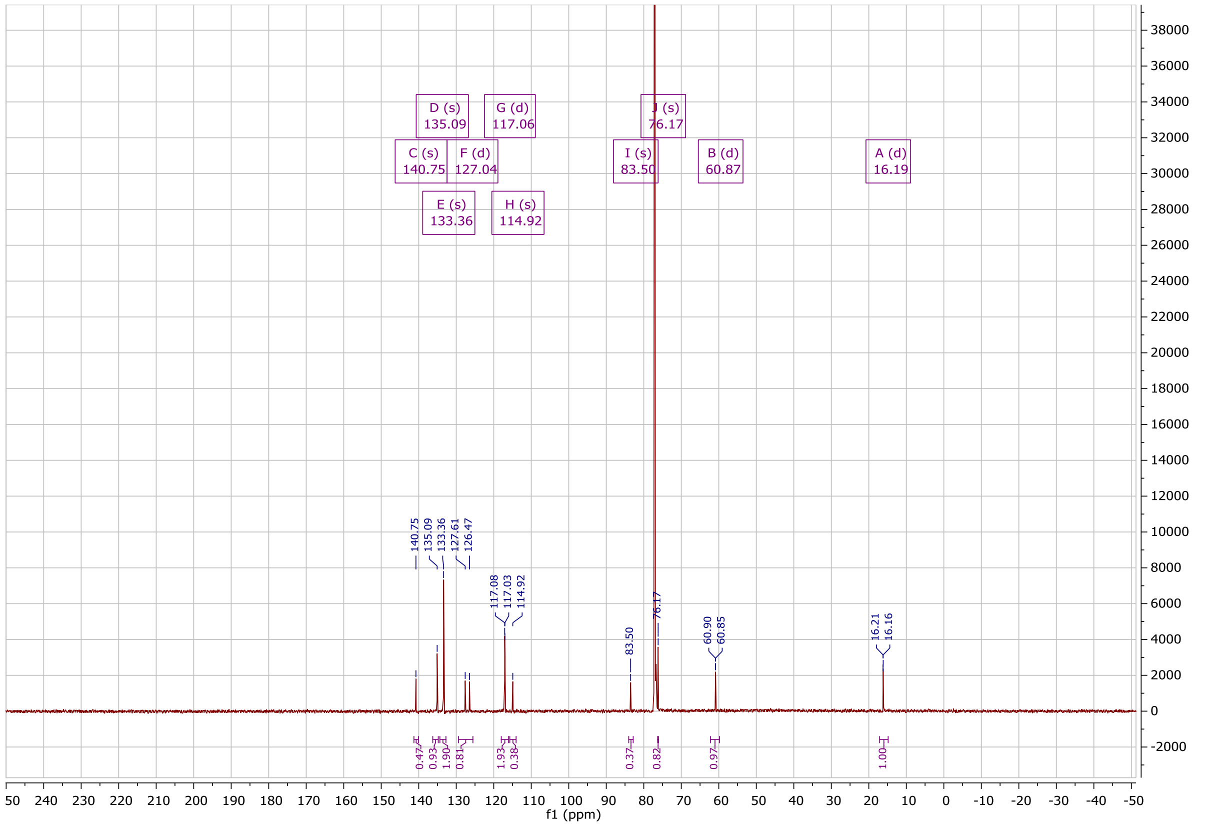

D (s) 135.09
G (d) 117.06
J (s) 76.17
C (s) 140.75
F (d) 127.04
I (s) 83.50
B (d) 60.87
A (d) 16.19
E (s) 133.36
H (s) 114.92
f1 (ppm)

N-(3,5-bis(trifluoromethyl)phenyl)-2-chloroacetamide (3c):

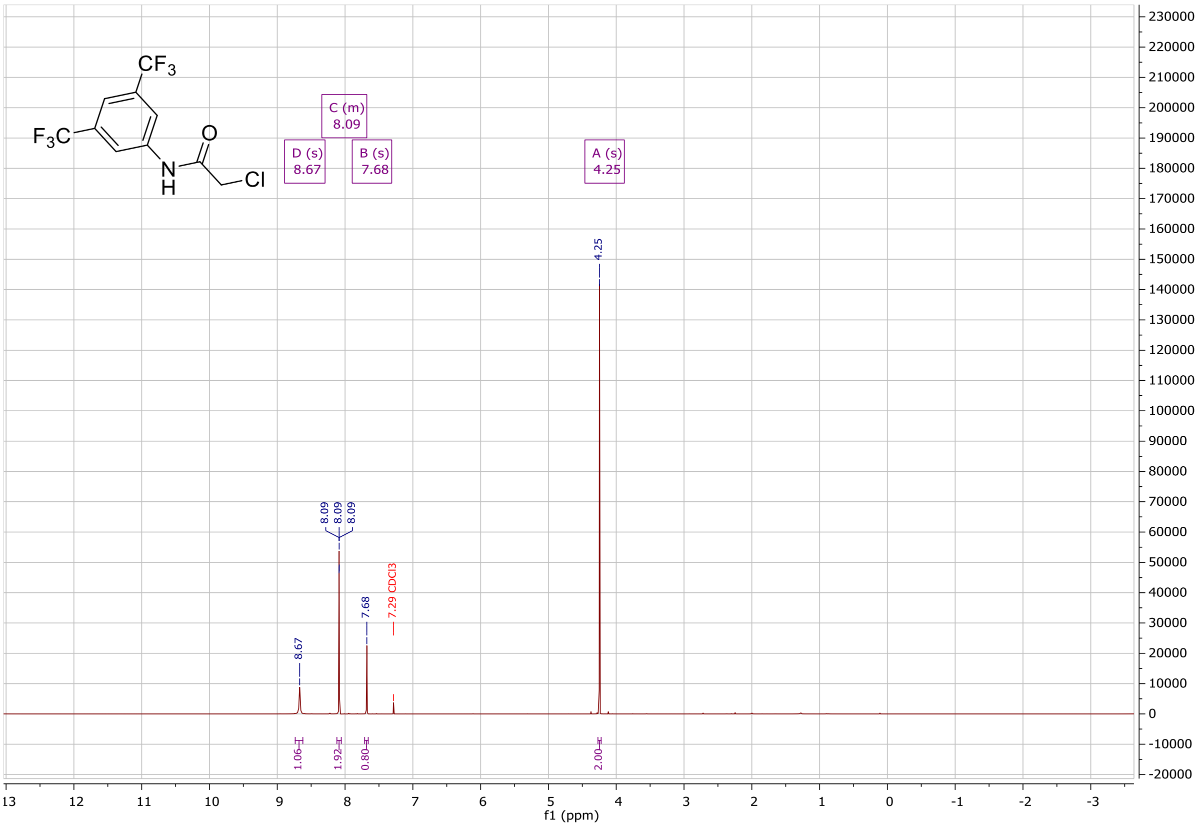

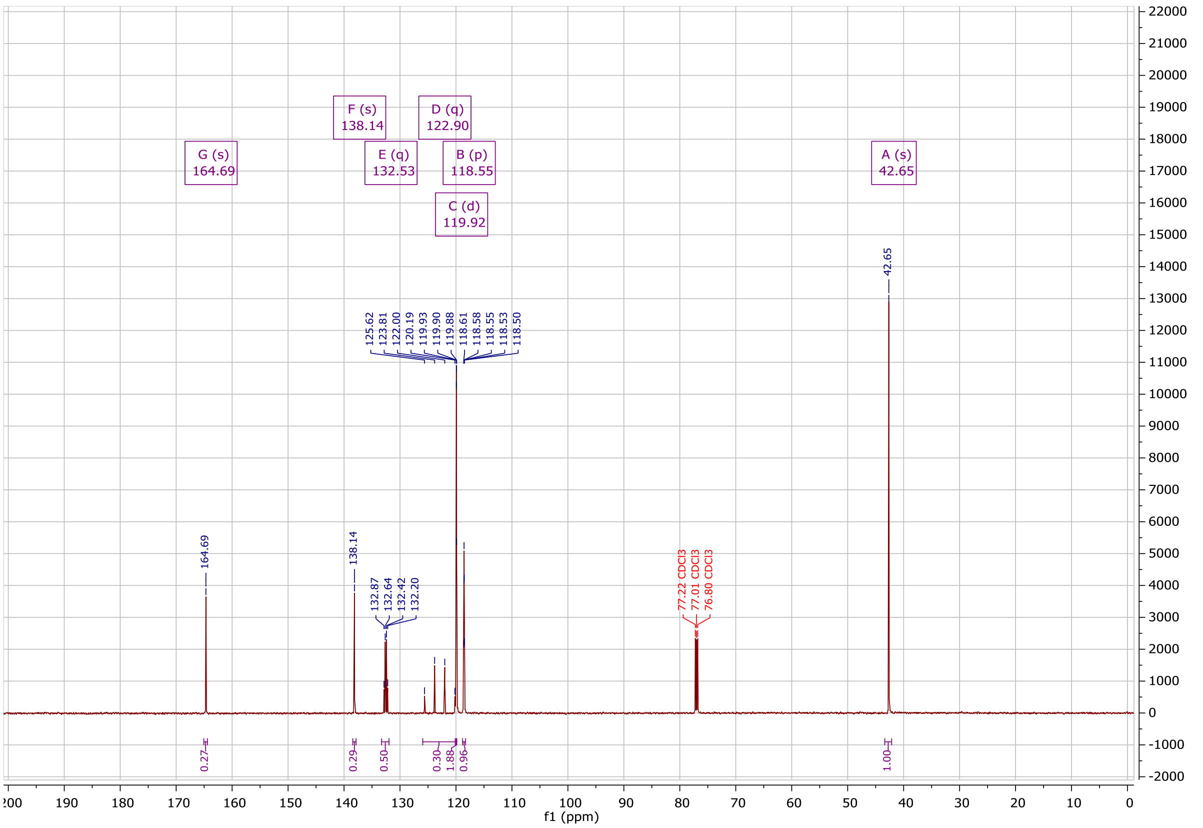

***N*-(3,5-bis(trifluoromethyl)phenyl)acrylamide (2c):**

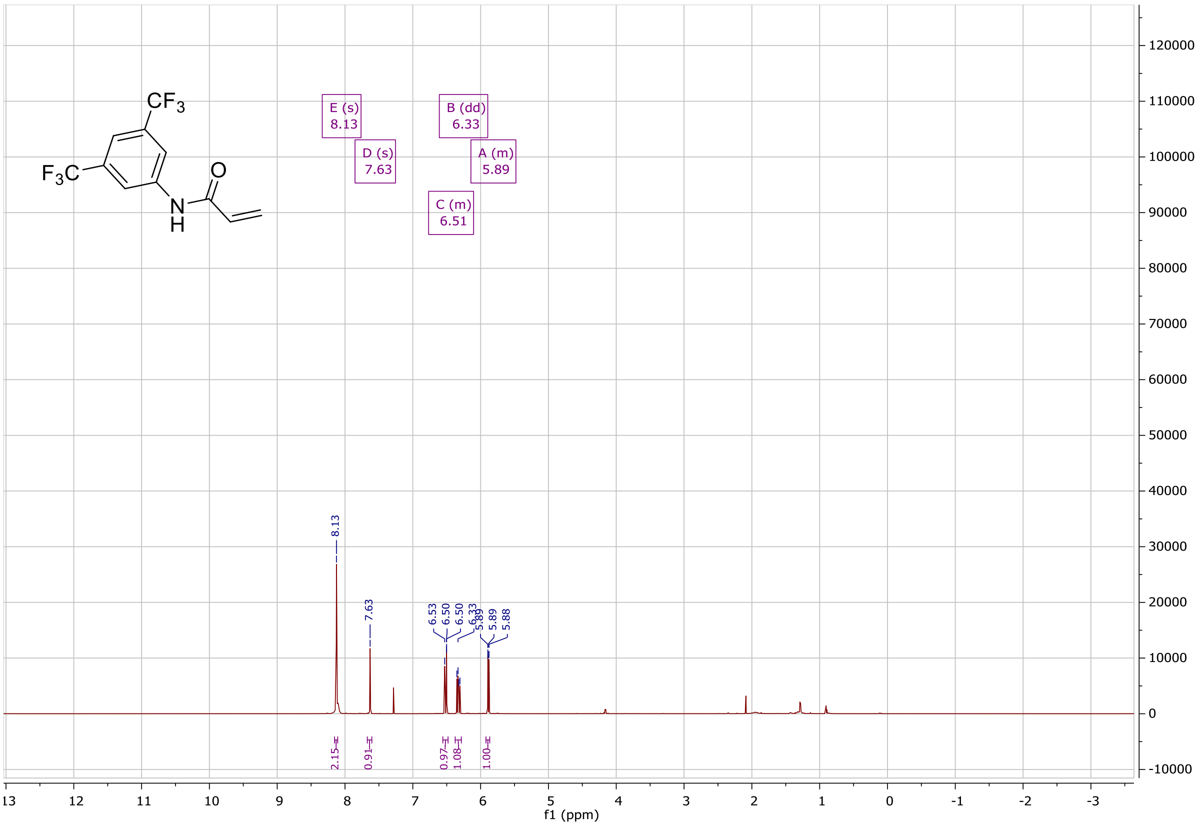

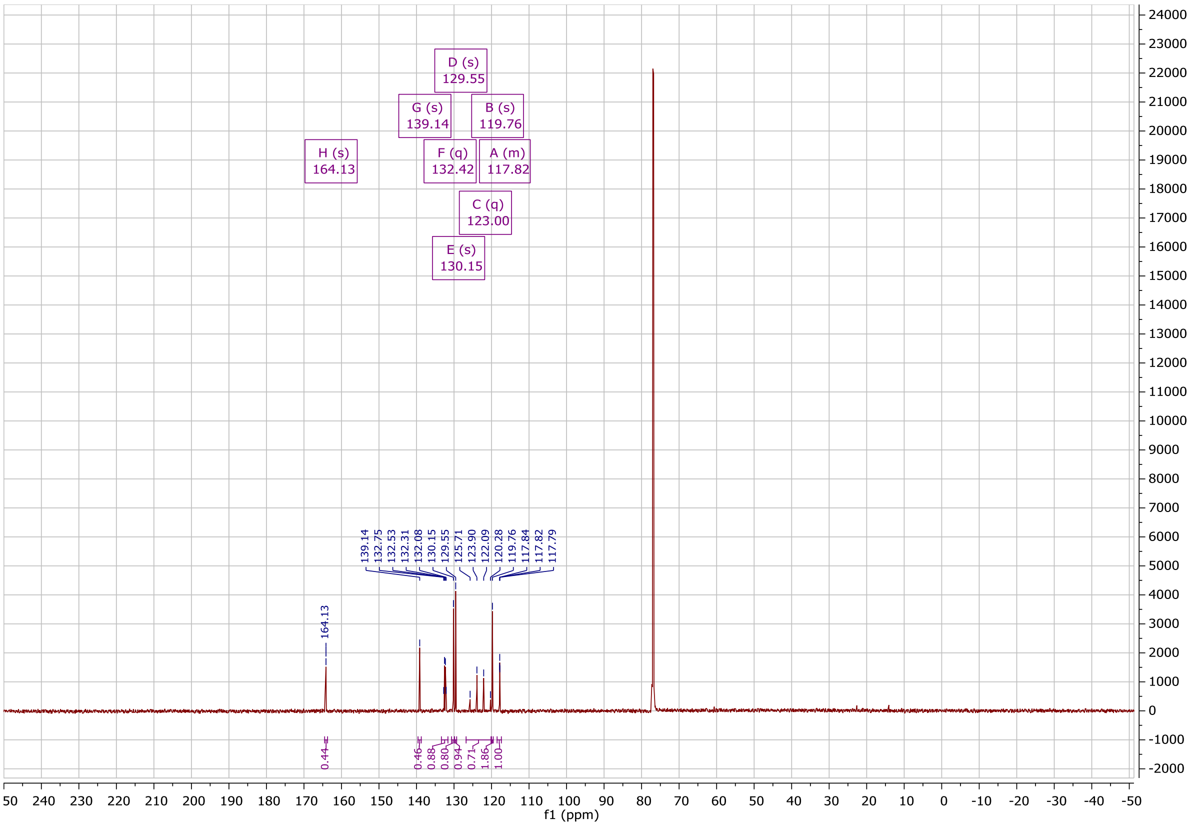

1-azido-3,5-bis(trifluoromethyl)benzene:

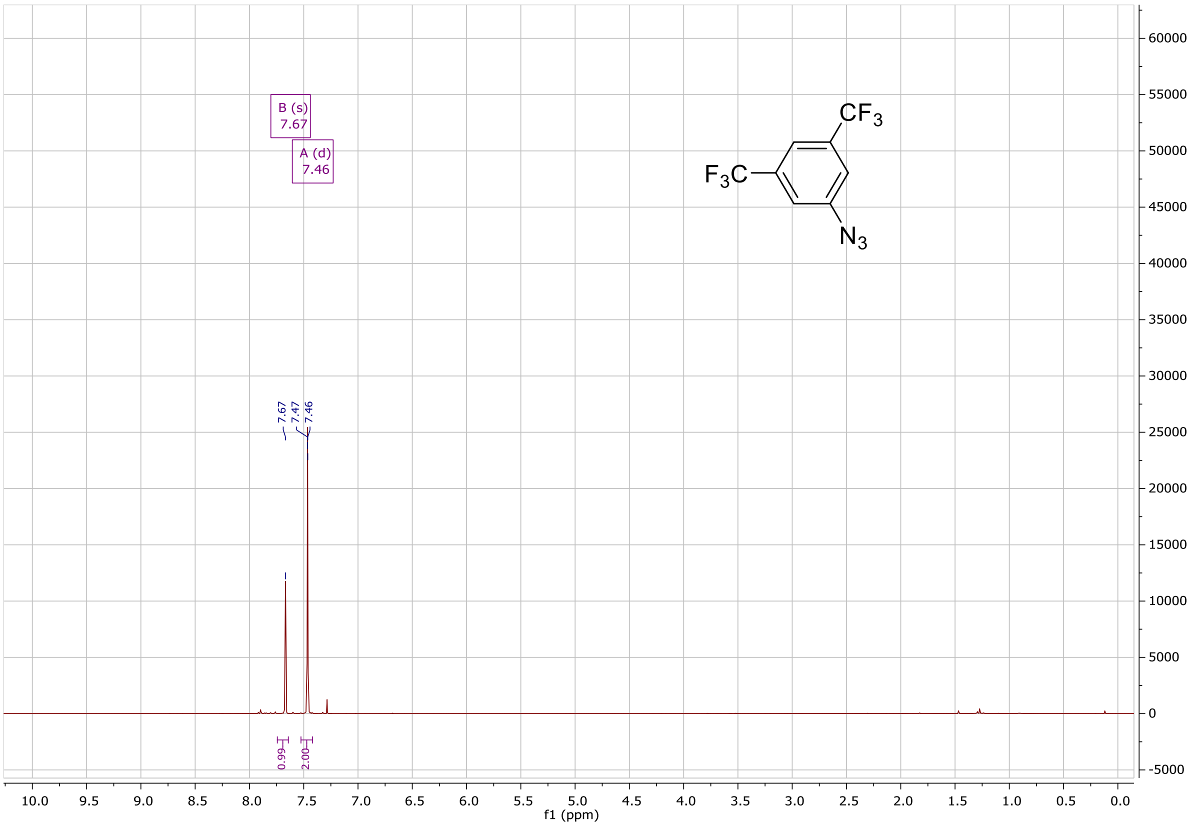

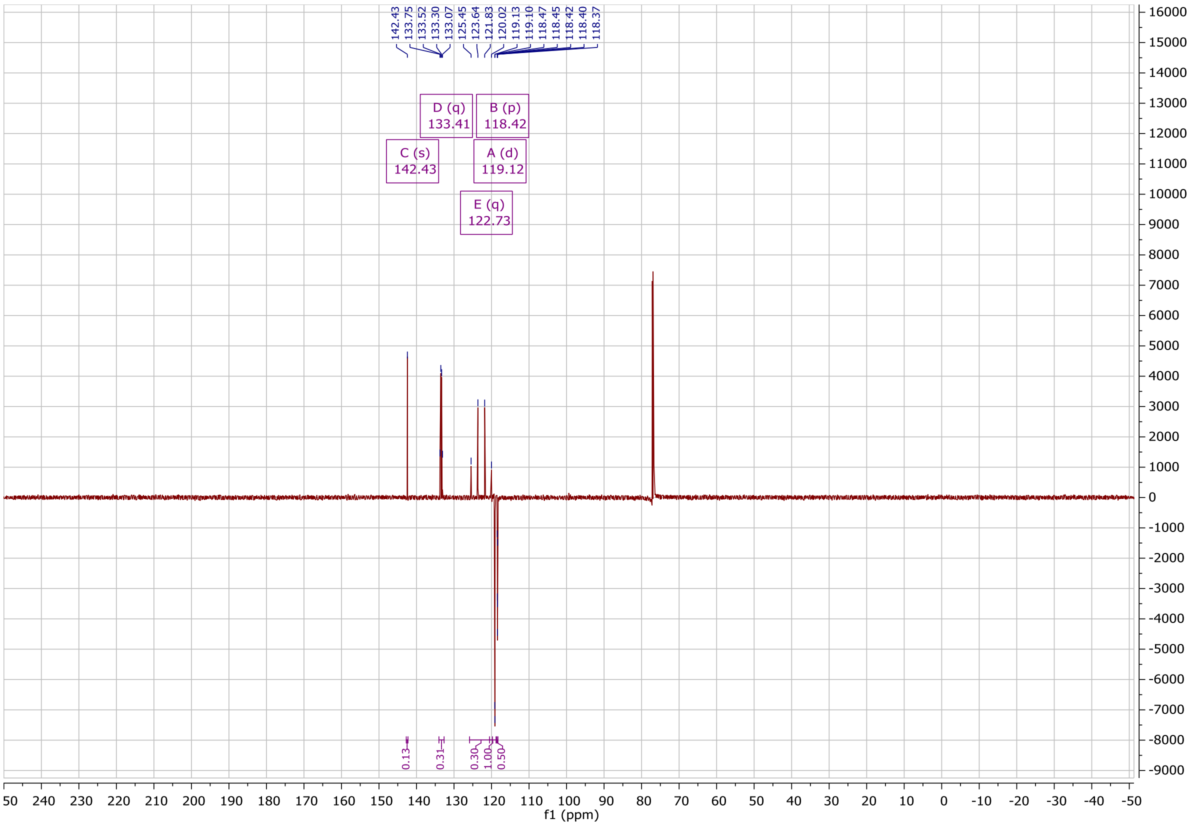

ethyl N-(3,5-bis(trifluoromethyl)phenyl)-P-vinylphosphonamidate (1c):

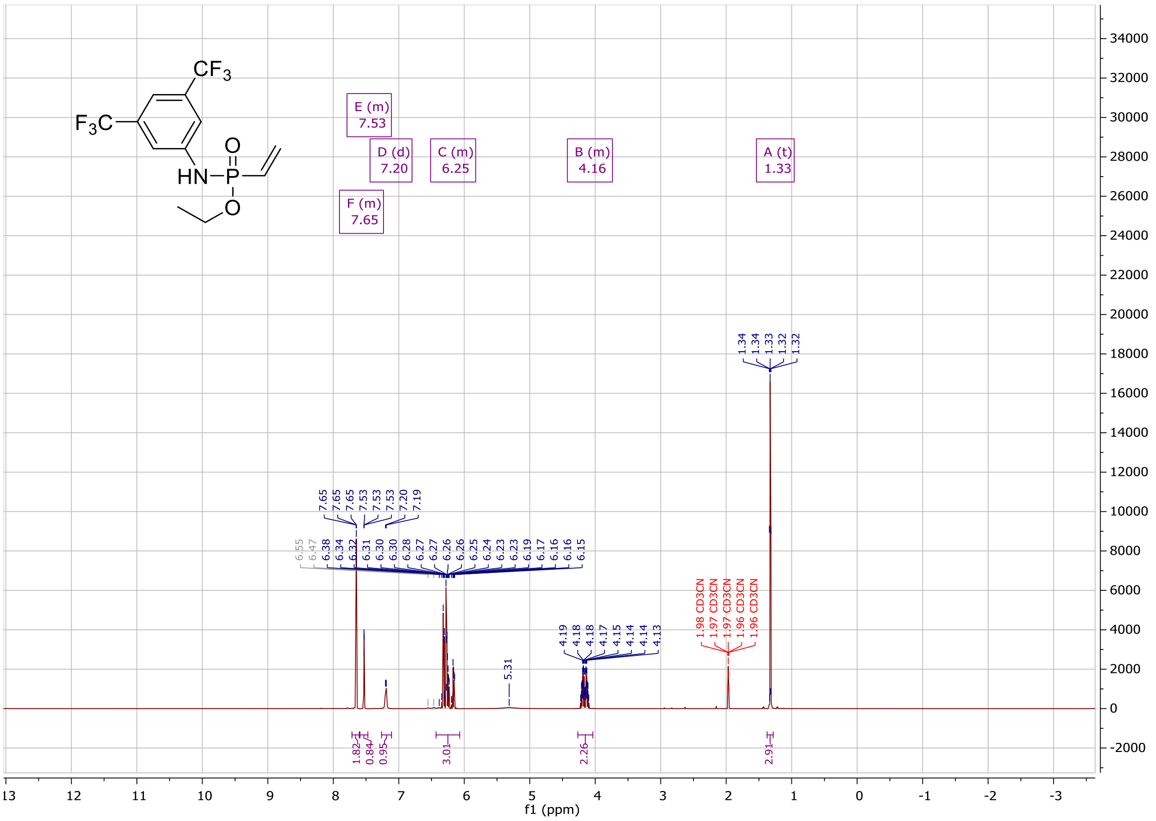

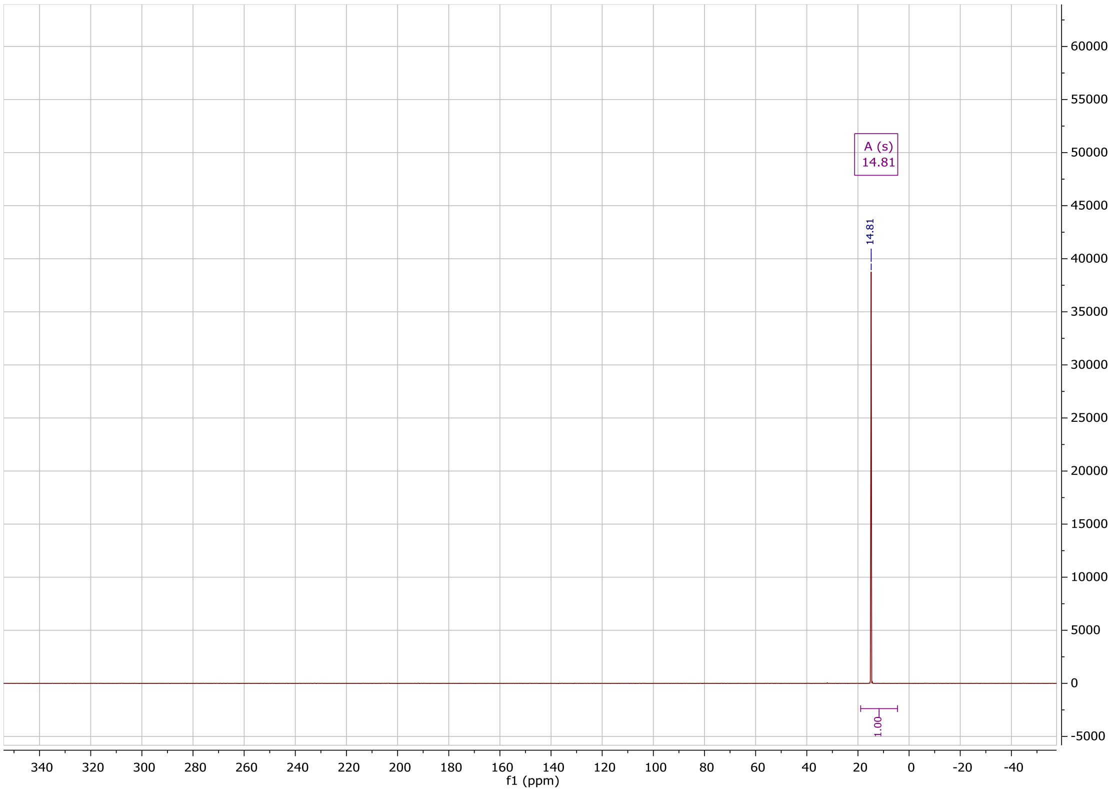

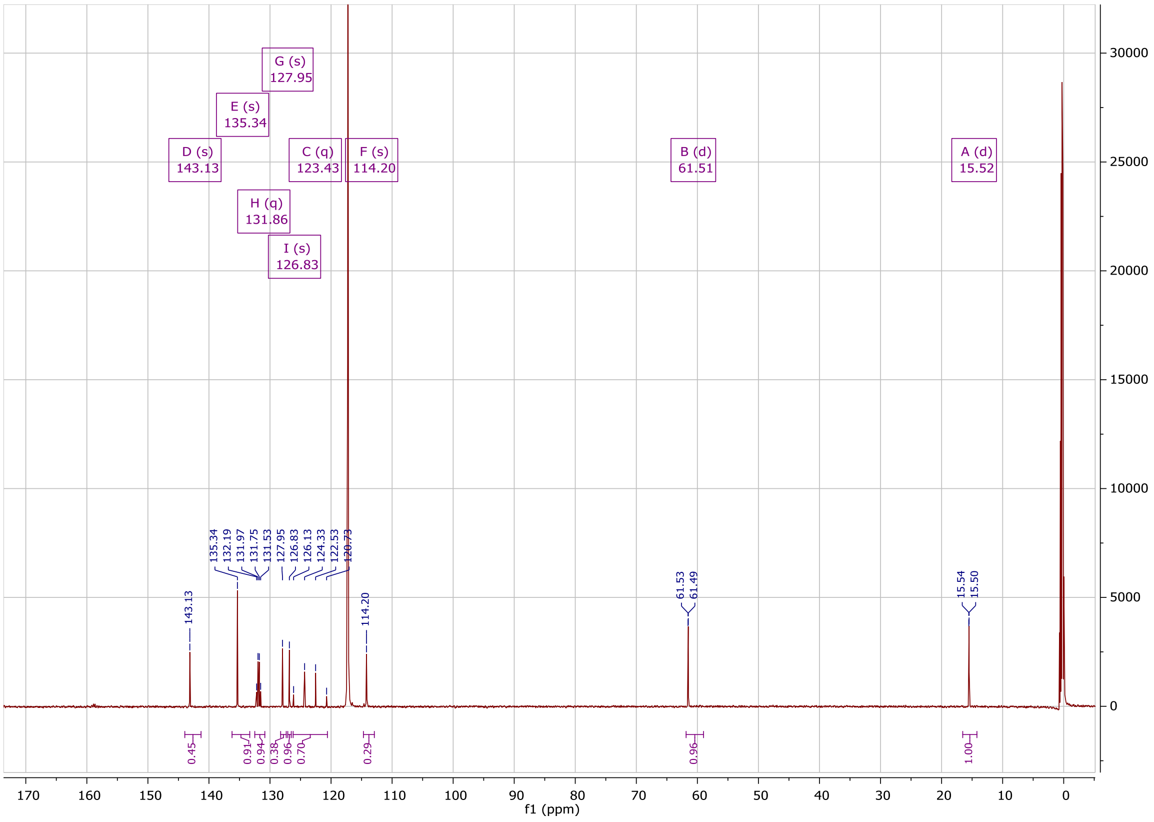

G (s) 127.95
E (s) 135.34
D (s) 143.13
C (q) 123.43
F (s) 114.20
B (d) 61.51
A (d) 15.52
H (q) 131.86
I (s) 126.83
143.13
135.34
132.19
131.97
131.75
131.53
127.95
126.83
126.13
124.33
122.53
120.73
114.20
61.53
61.49
15.54
15.50
0.45
0.91
0.94
0.38
0.96
0.70
0.29
0.96
1.00
f1 (ppm)

Afatinib-N$_3$ (S2):

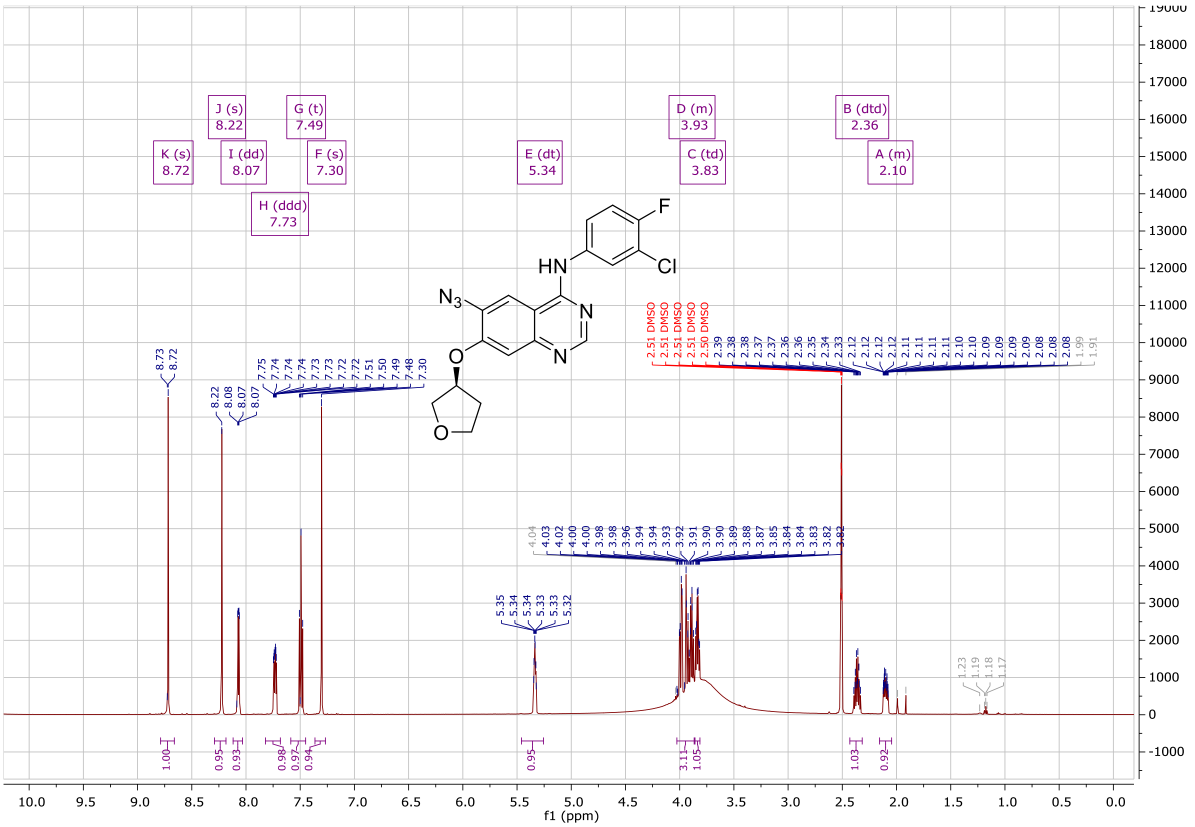

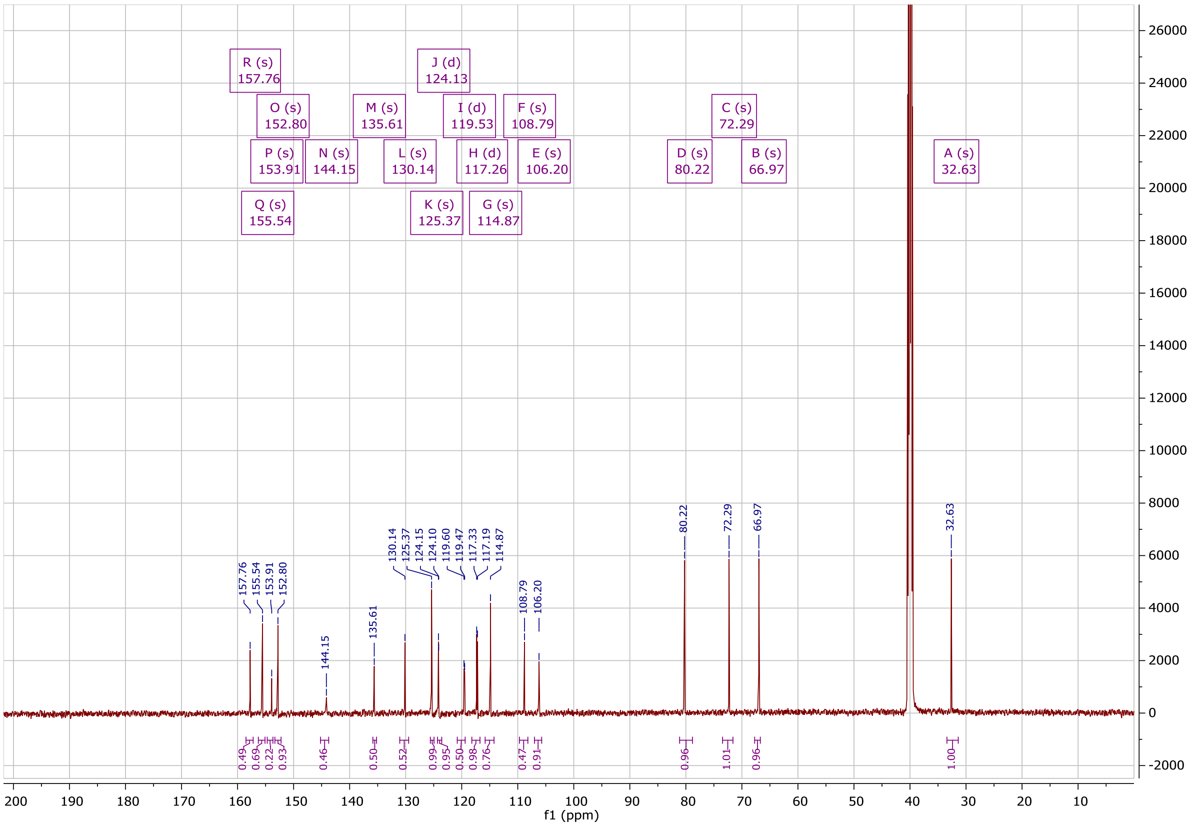

Afatinib-vinyl-phosphonamidate (4a):

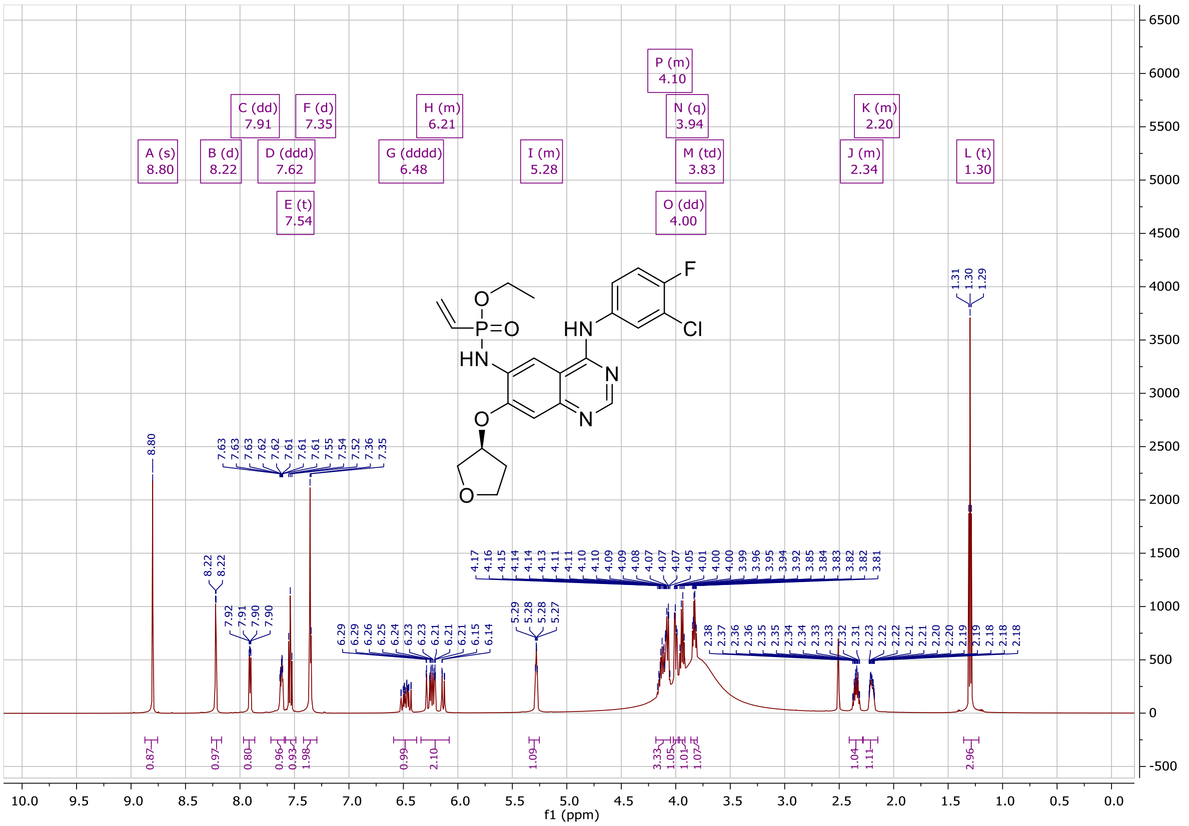

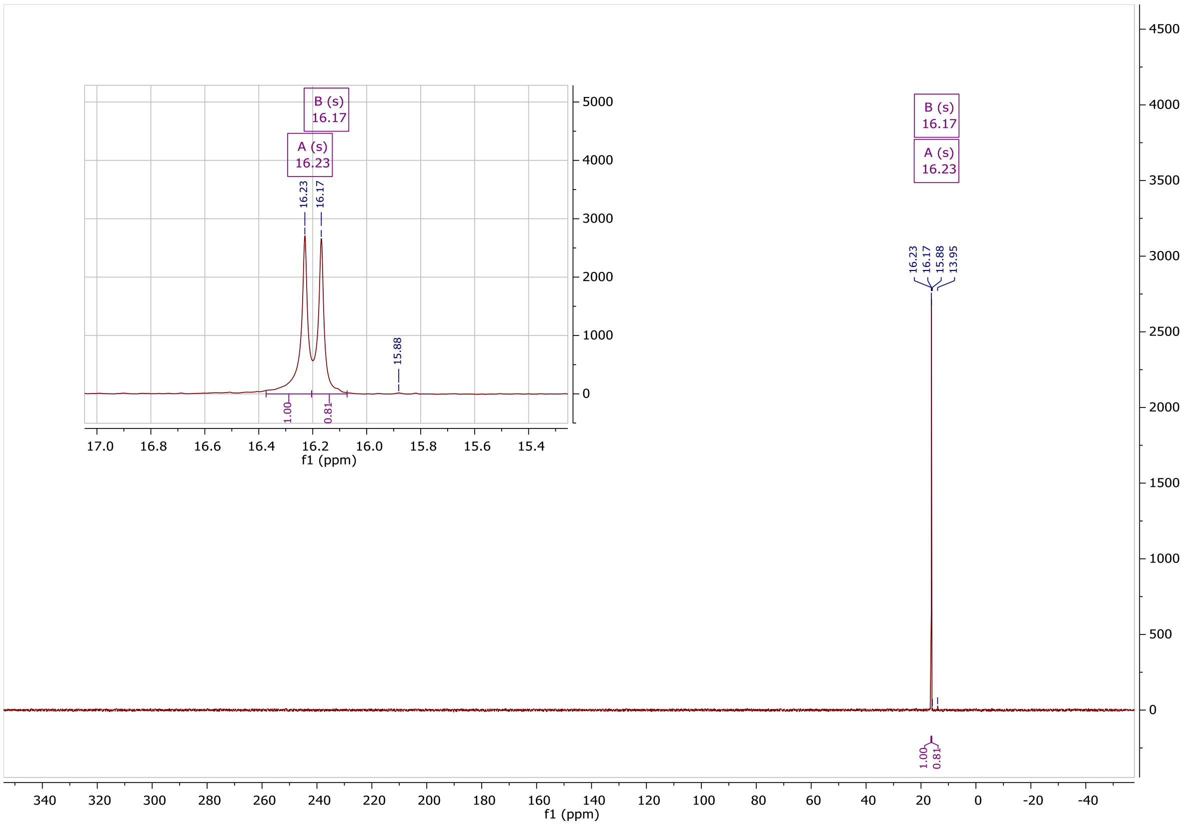

Afatinib-ethyl-phosphonamidate (4c):

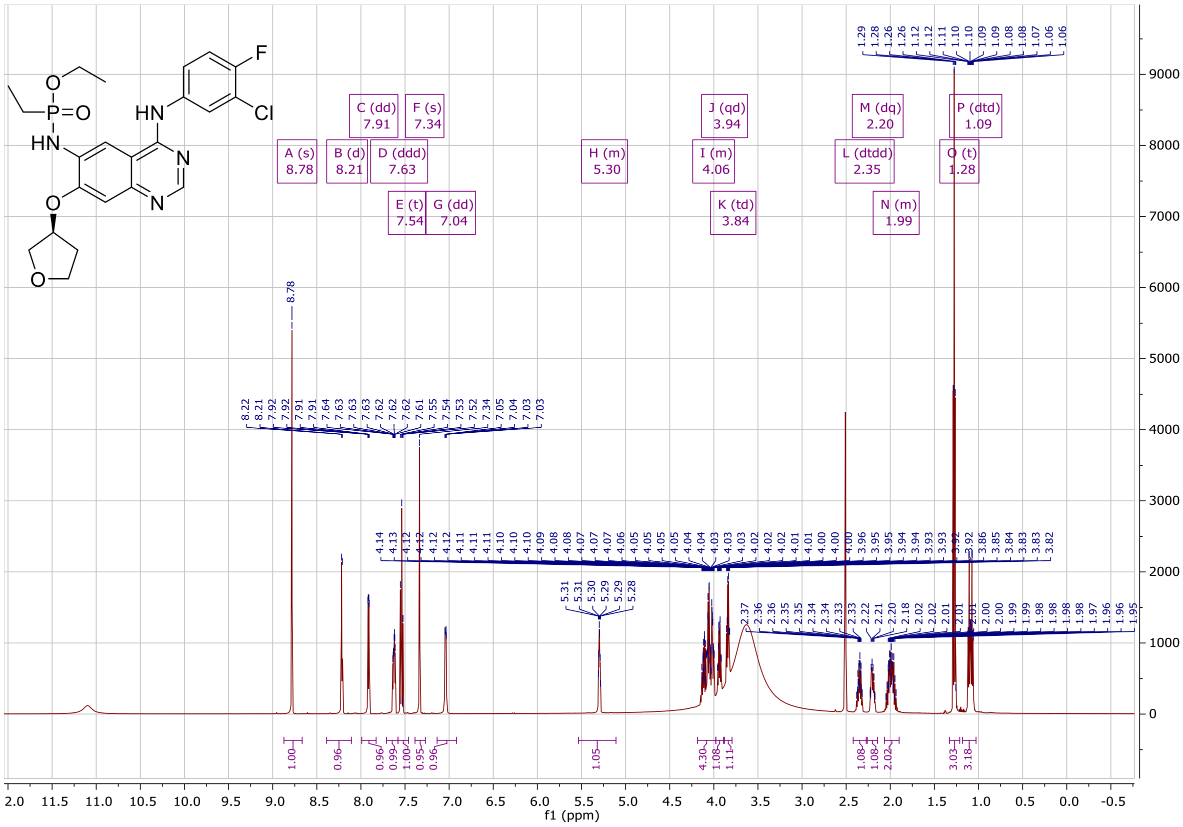

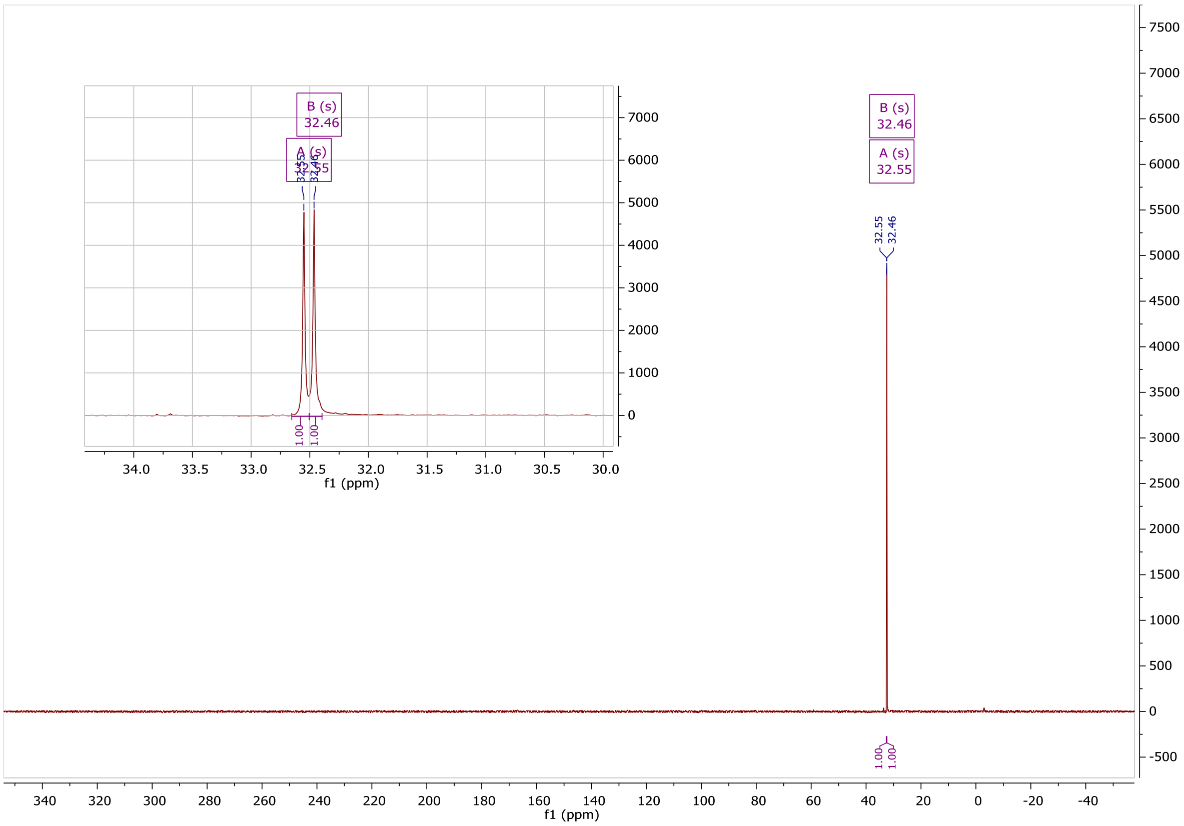

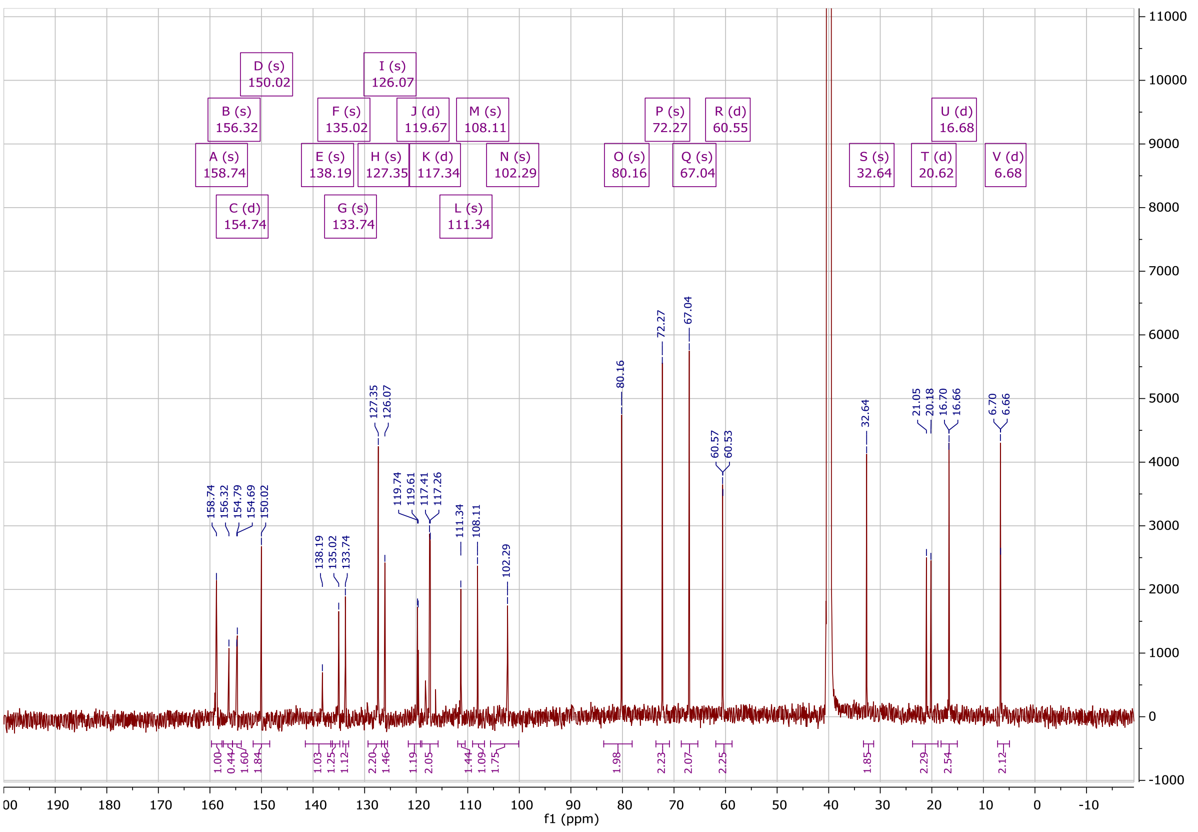

but-3-yn-1-yl N-(4-((3-chloro-4-fluorophenyl)amino)-7-(((S)-tetrahydrofuran-3-yl)oxy)quinazolin-6-yl)-P-vinylphosphonamidate (4b):

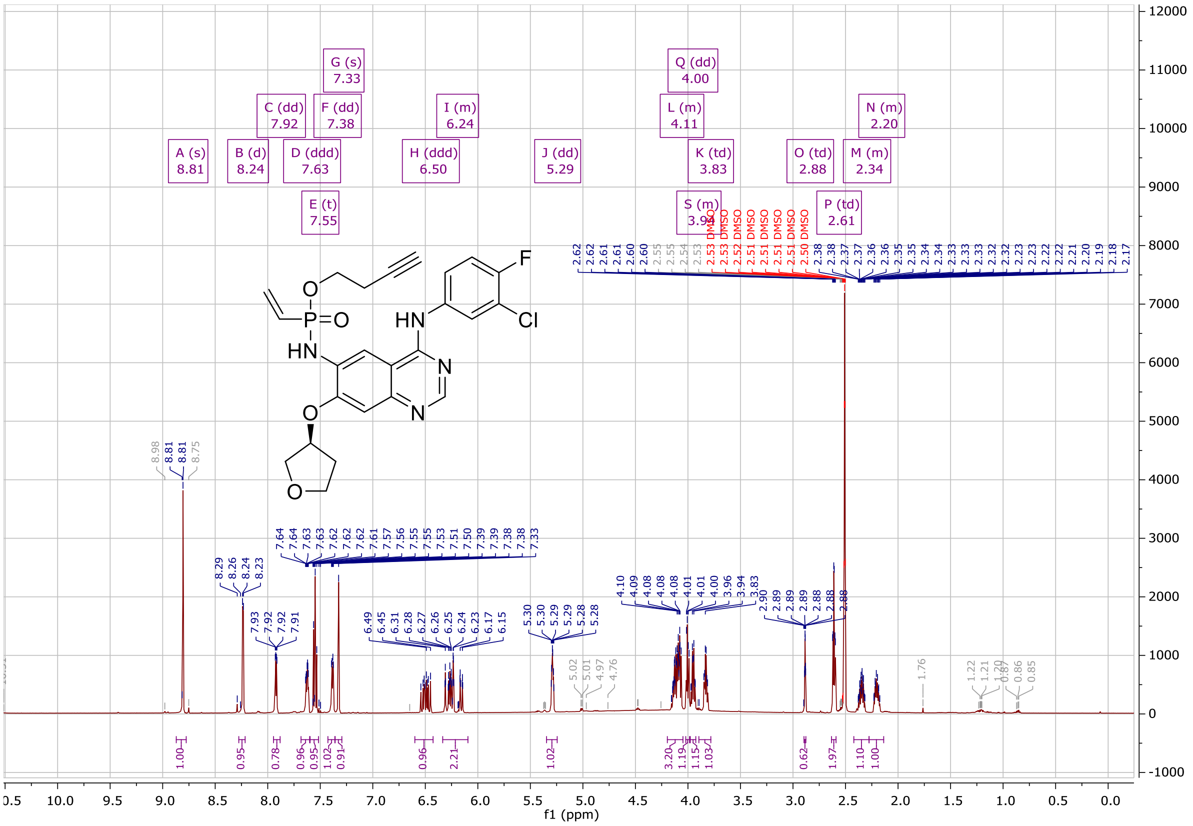

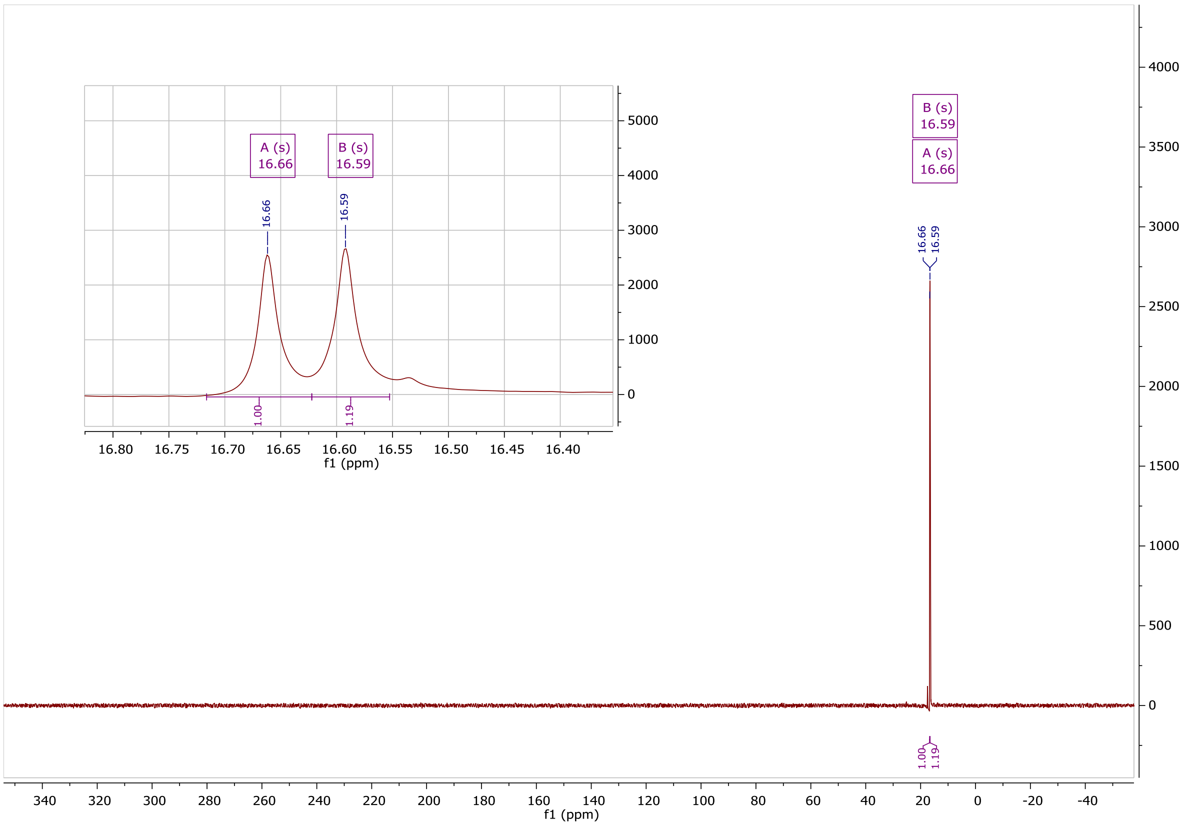

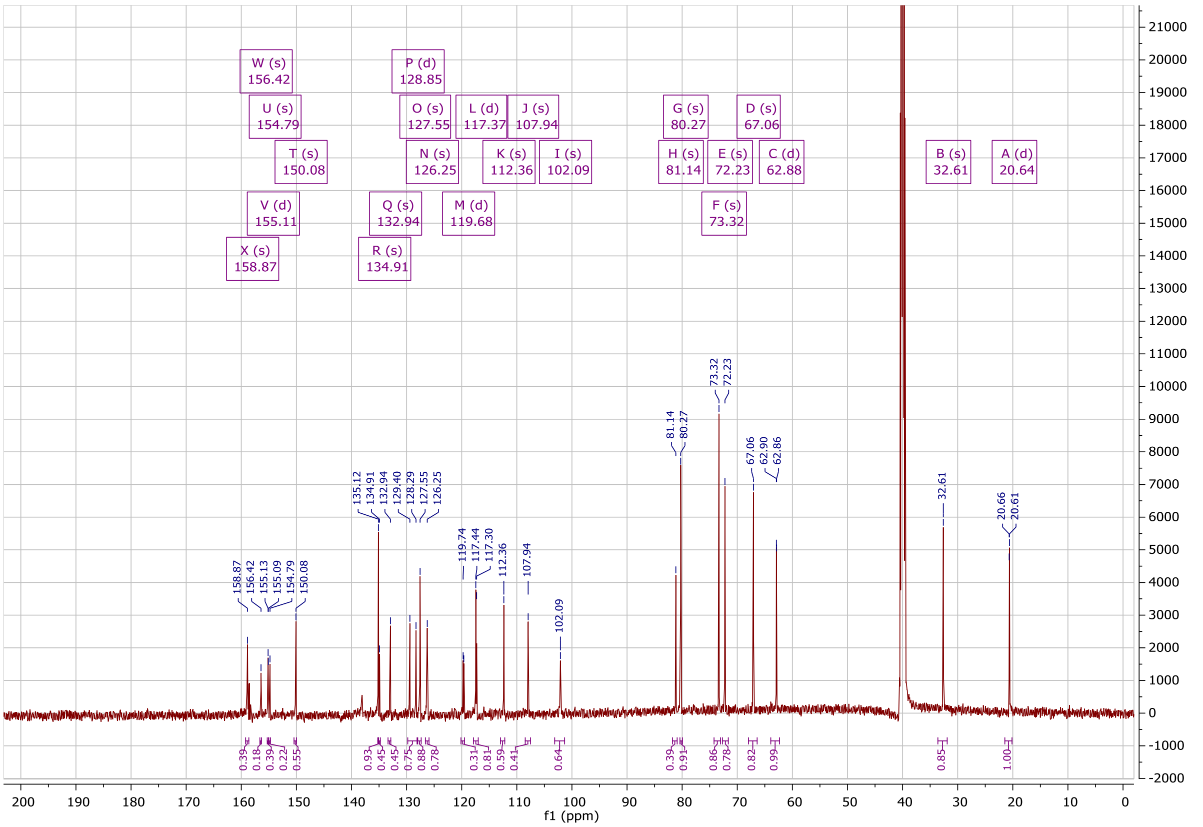

W (s) 156.42
P (d) 128.85
U (s) 154.79
O (s) 127.55
L (d) 117.37
J (s) 107.94
G (s) 80.27
D (s) 67.06
T (s) 150.08
N (s) 126.25
K (s) 112.36
I (s) 102.09
H (s) 81.14
E (s) 72.23
C (d) 62.88
B (s) 32.61
A (d) 20.64
V (d) 155.11
Q (s) 132.94
M (d) 119.68
F (s) 73.32
X (s) 158.87
R (s) 134.91
f1 (ppm)

6-azido-N-(3-chloro-4-fluorophenyl)-7-(prop-2-yn-1-yloxy)quinazolin-4-amine (S4):

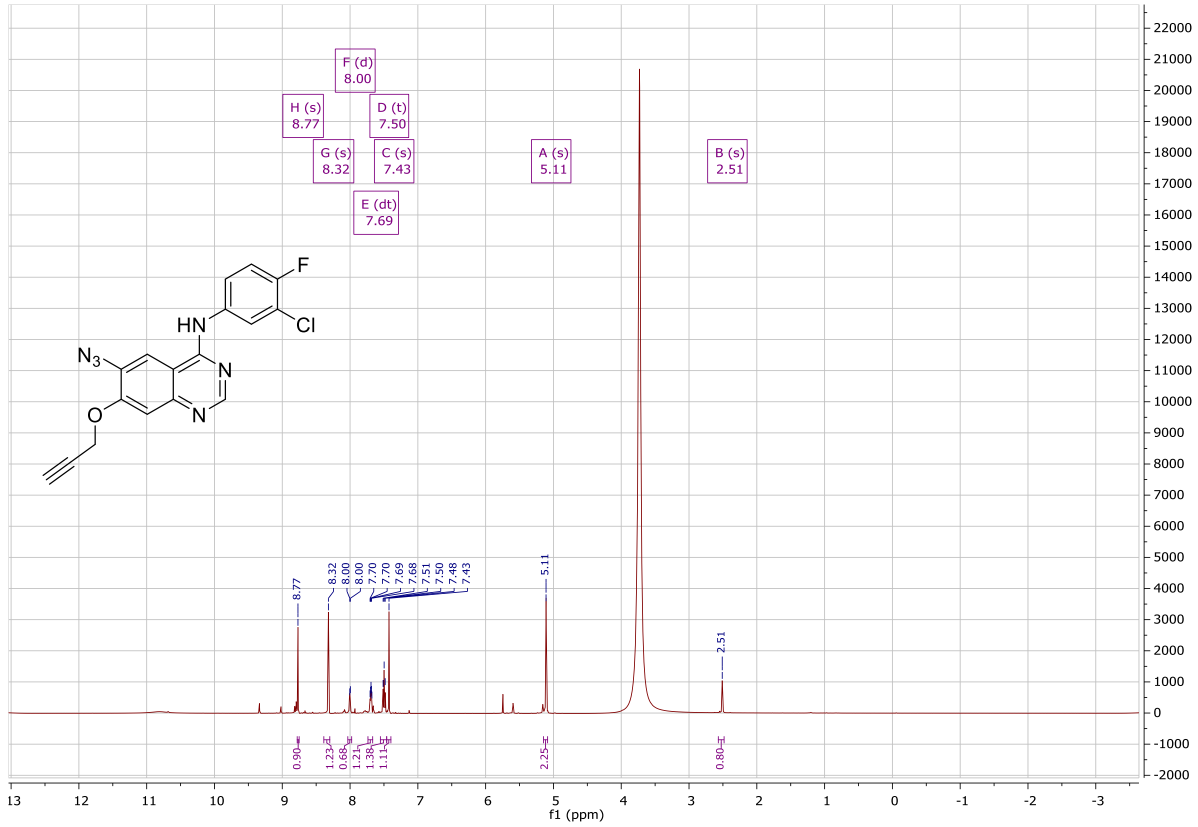

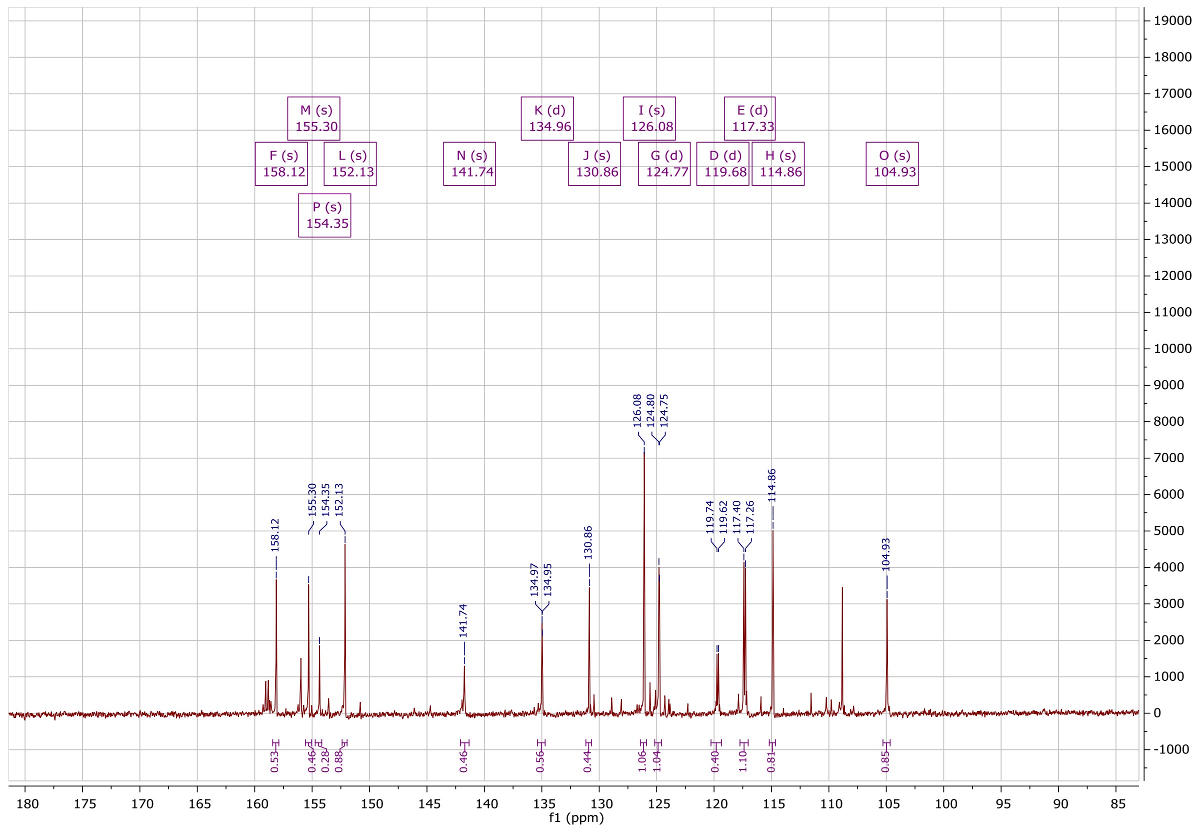

ethyl N-(4-((3-chloro-4-fluorophenyl)amino)-7-(prop-2-yn-1-yloxy)quinazolin-6-yl)-P-vinyl-phosphonamidate (**5**):

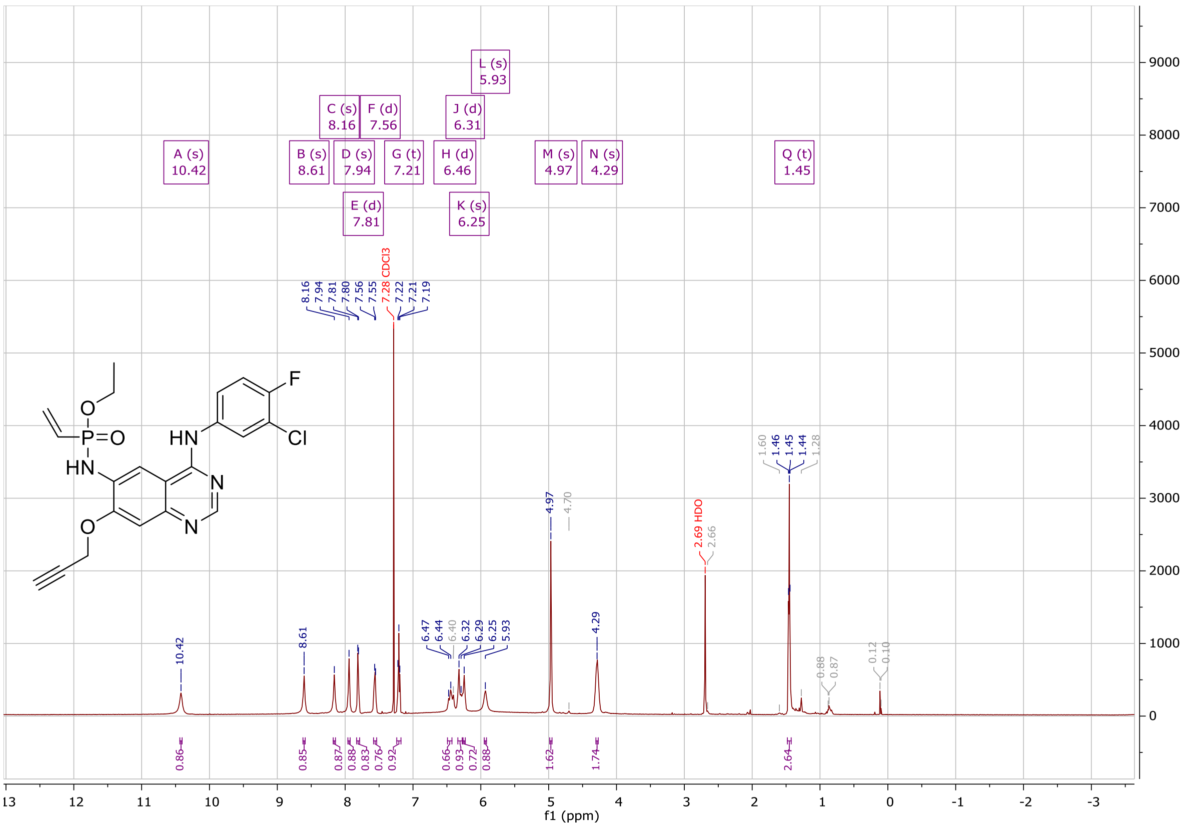

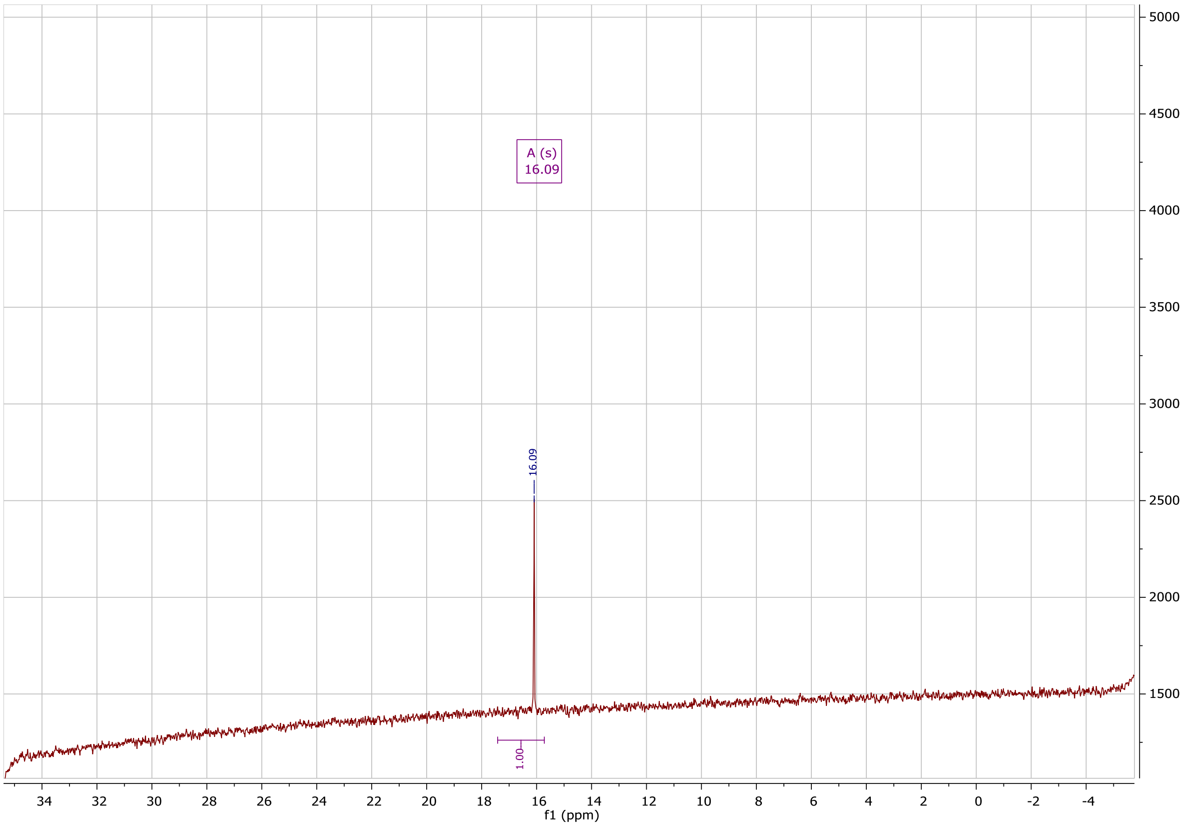

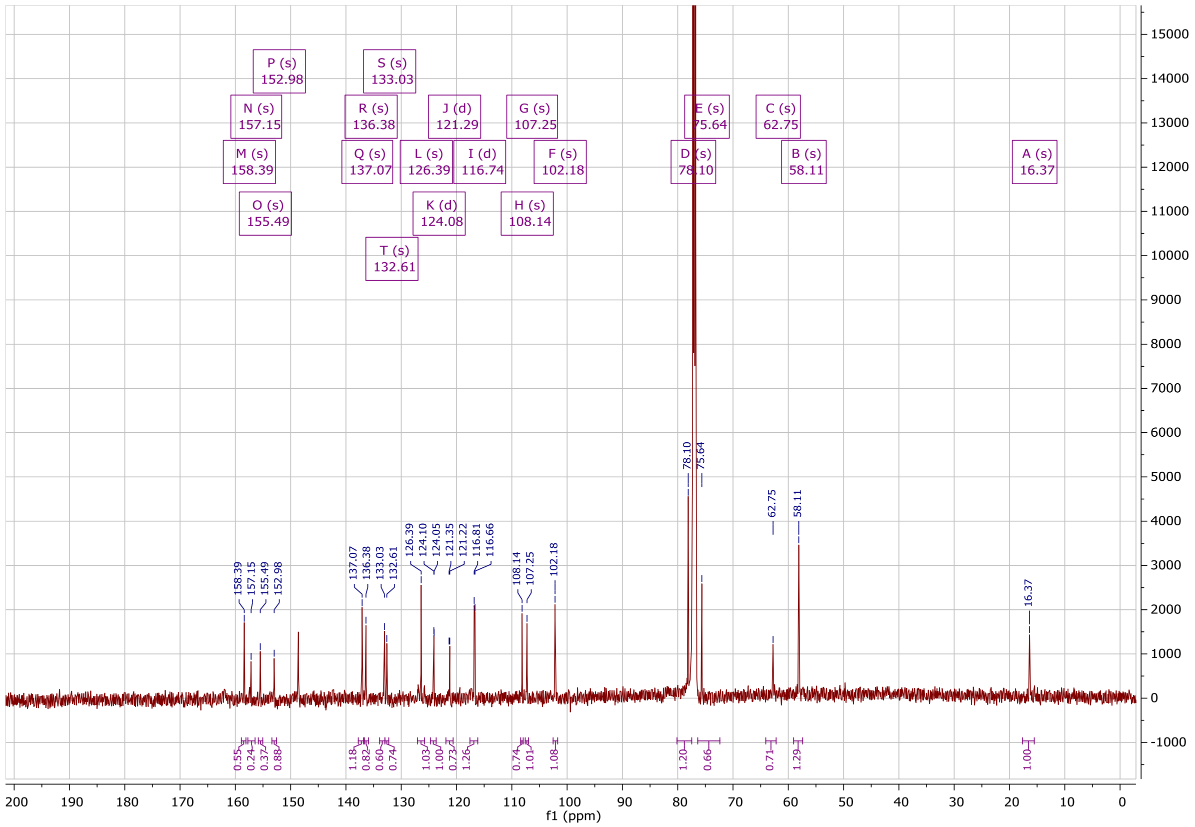

P (s) 152.98
S (s) 133.03
N (s) 157.15
R (s) 136.38
J (d) 121.29
G (s) 107.25
E (s) 75.64
C (s) 62.75
M (s) 158.39
Q (s) 137.07
L (s) 126.39
I (d) 116.74
F (s) 102.18
D (s) 78.10
B (s) 58.11
A (s) 16.37
O (s) 155.49
K (d) 124.08
H (s) 108.14
T (s) 132.61
f1 (ppm)

1-(2-azidobenzyl)-3-(4-phenoxyphenyl)-1H-pyrazolo[3,4-d]pyrimidin-4-amine:

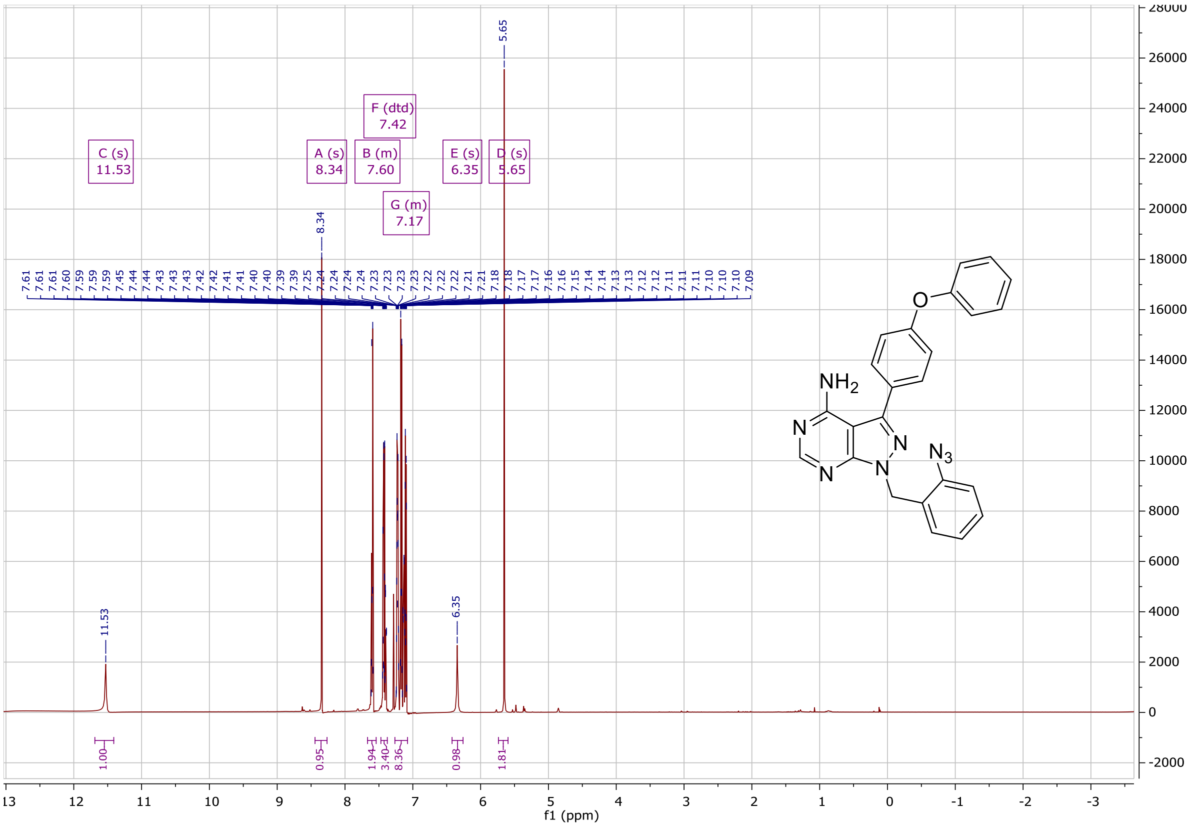

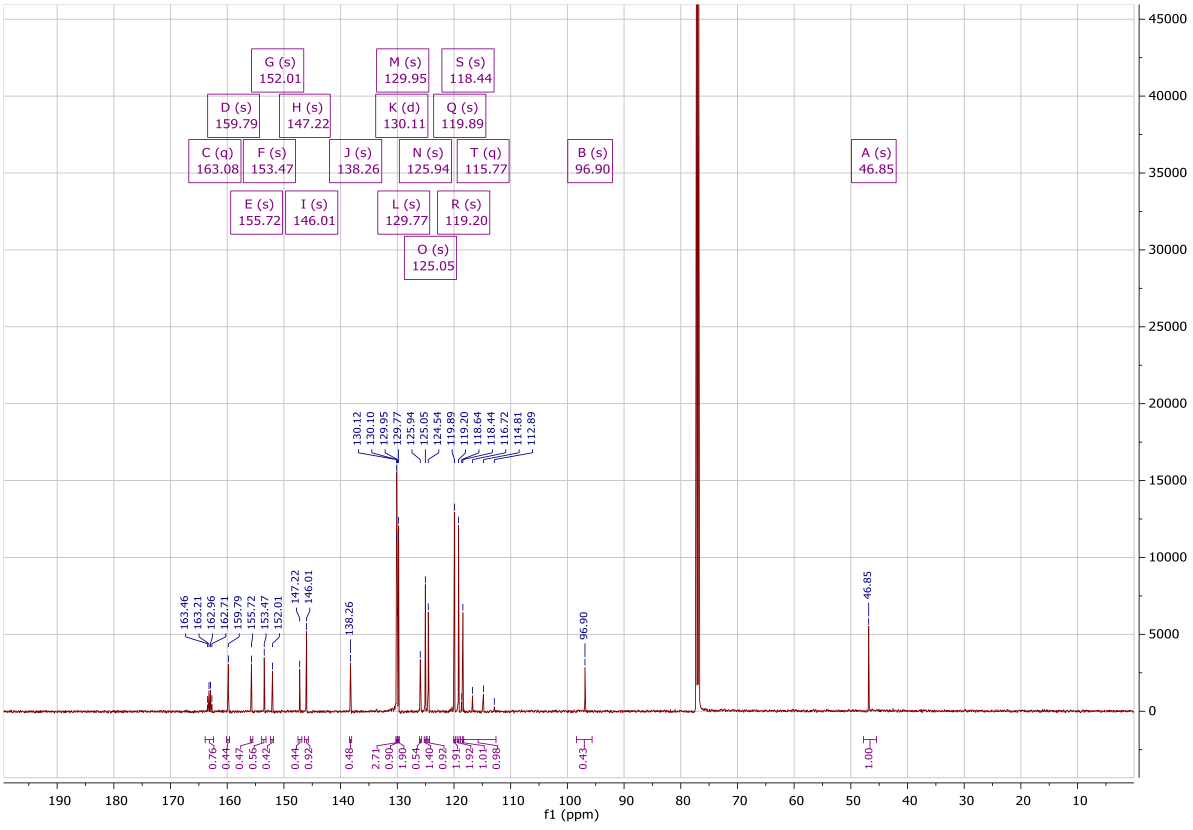

1-(3-azidobenzyl)-3-(4-phenoxyphenyl)-1H-pyrazolo[3,4-d]pyrimidin-4-amine:

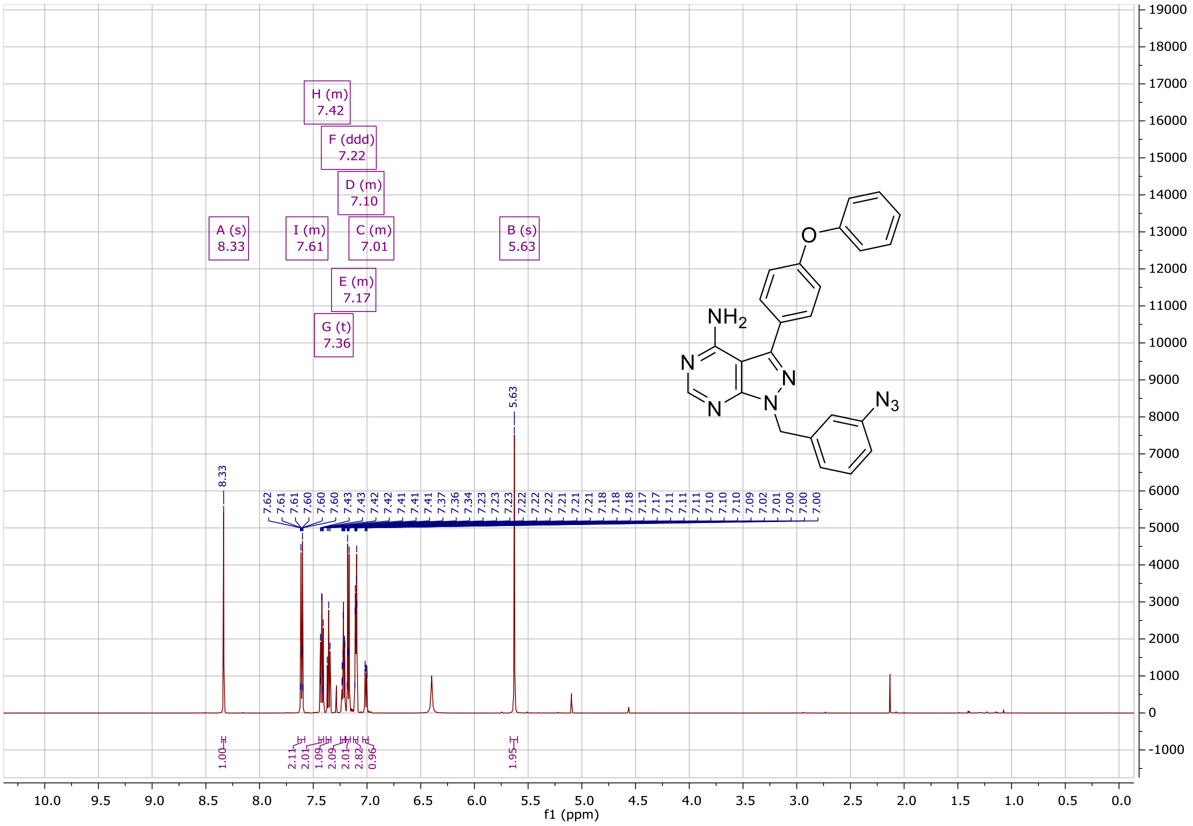

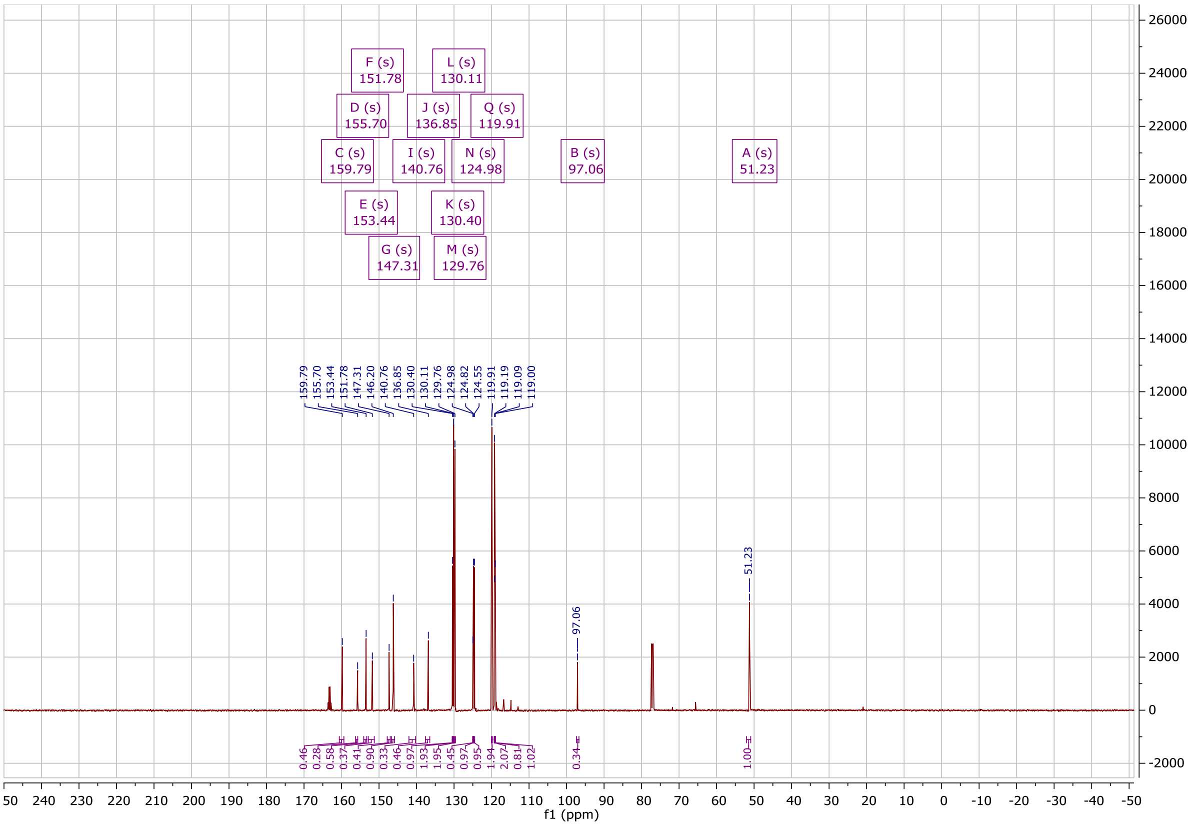

1-(4-azidobenzyl)-3-(4-phenoxyphenyl)-1H-pyrazolo[3,4-d]pyrimidin-4-amine:

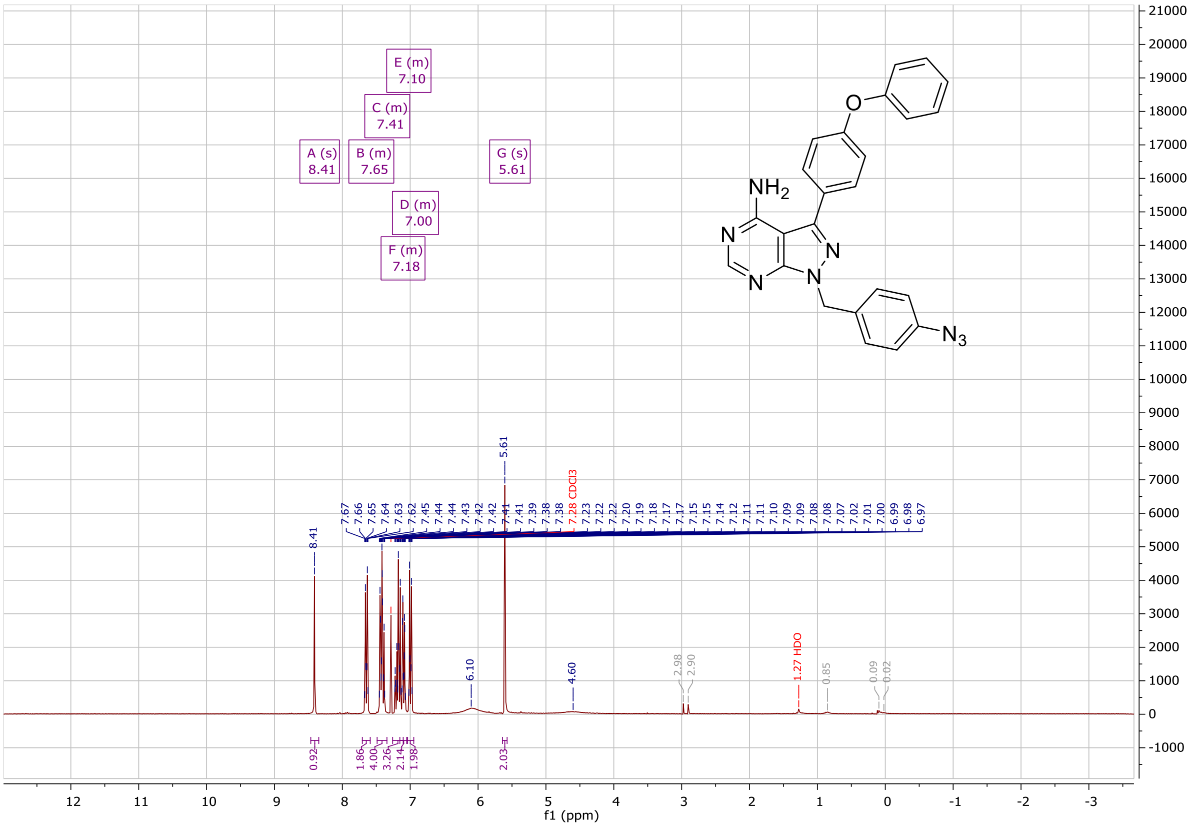

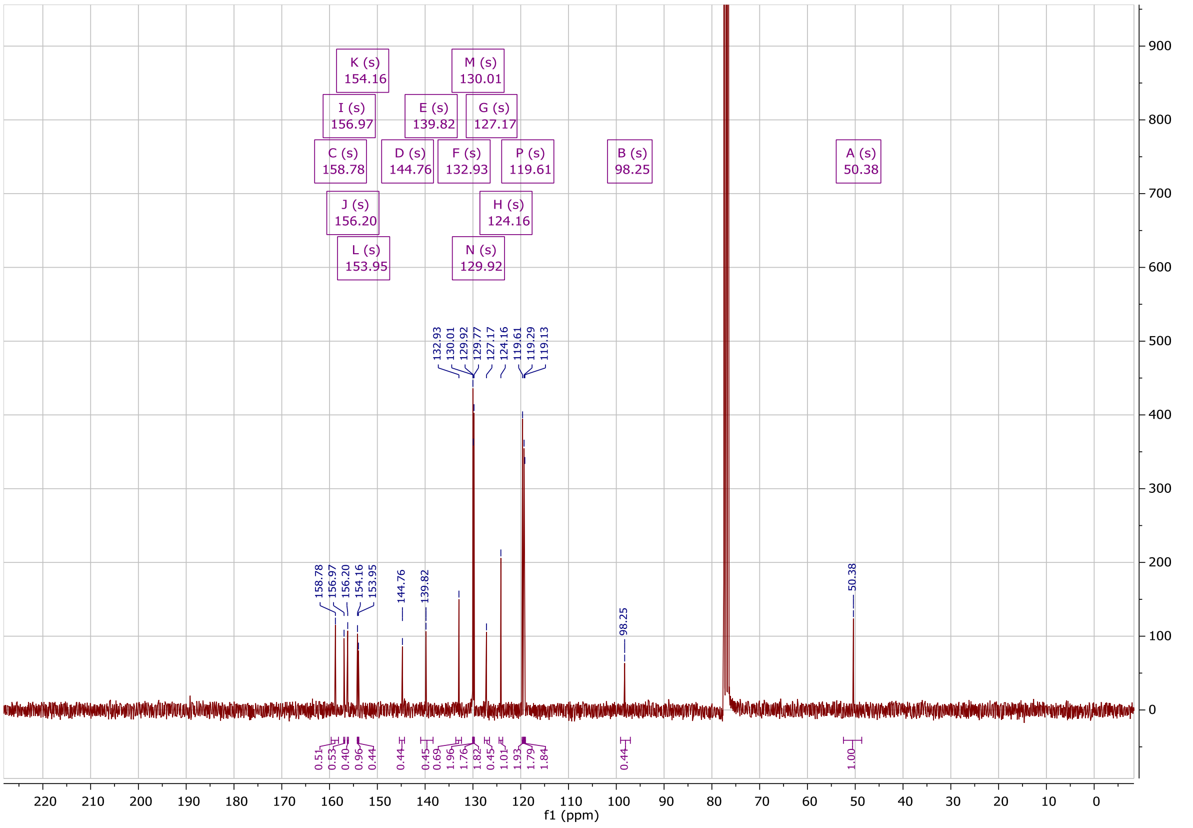

1-(2-azidophenethyl)-3-(4-phenoxyphenyl)-1H-pyrazolo[3,4-d]pyrimidin-4-amine:

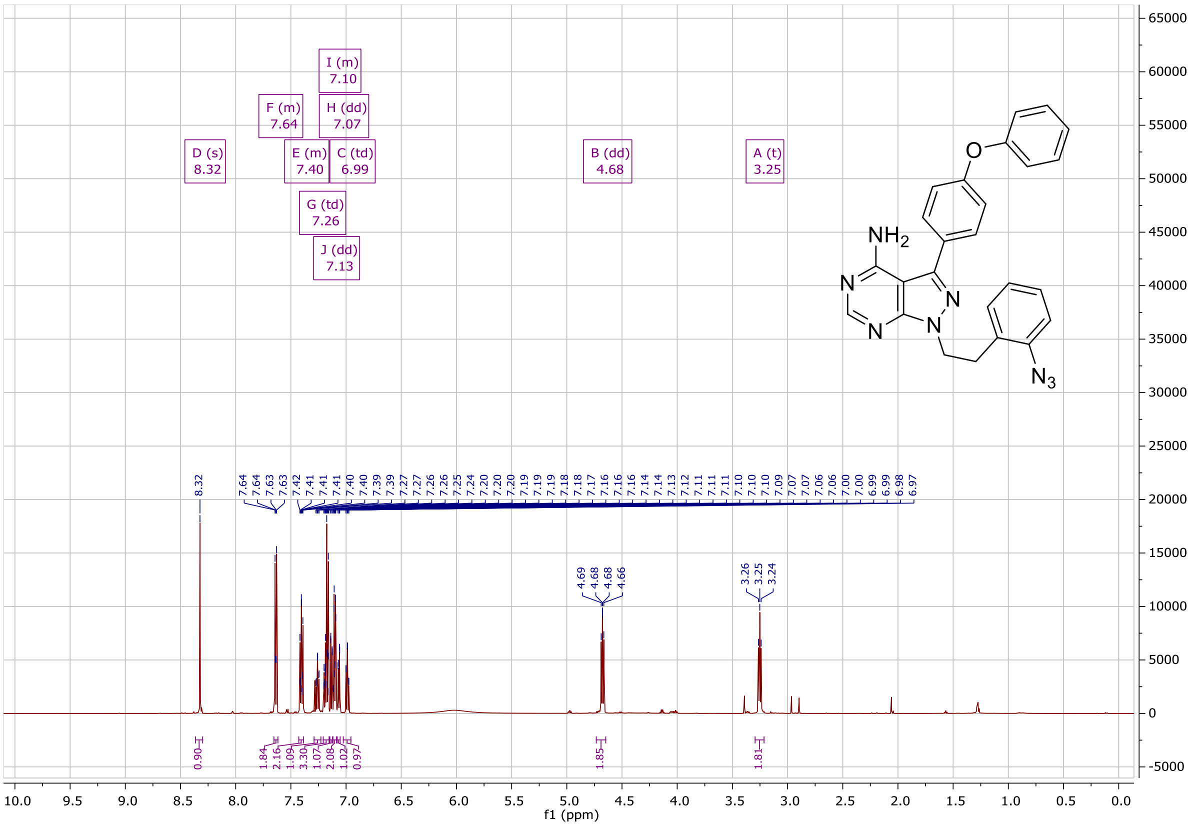

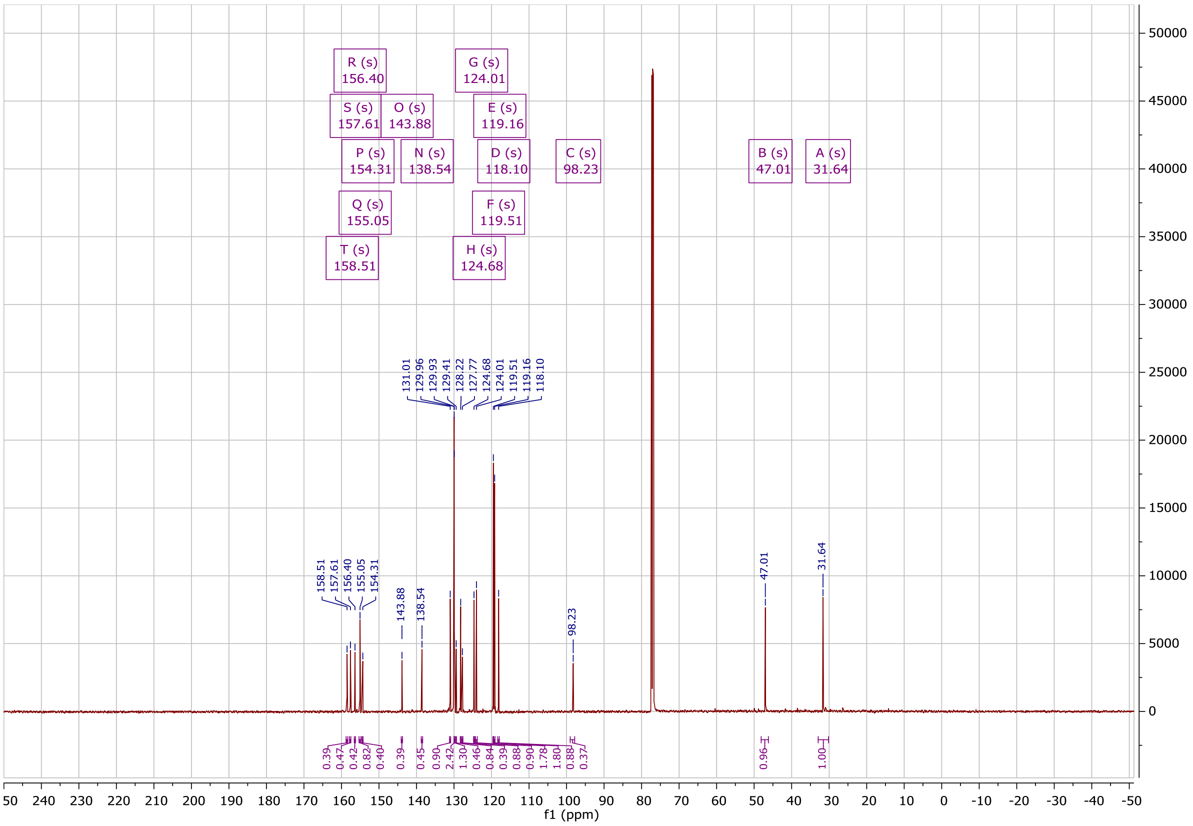

but-3-yn-1-yl N-(2-(2-(4-amino-3-(4-phenoxyphenyl)-1H-pyrazolo[3,4-d]pyrimidin-1-yl)ethyl) phenyl)-P-vinylphosphonamidate (8):

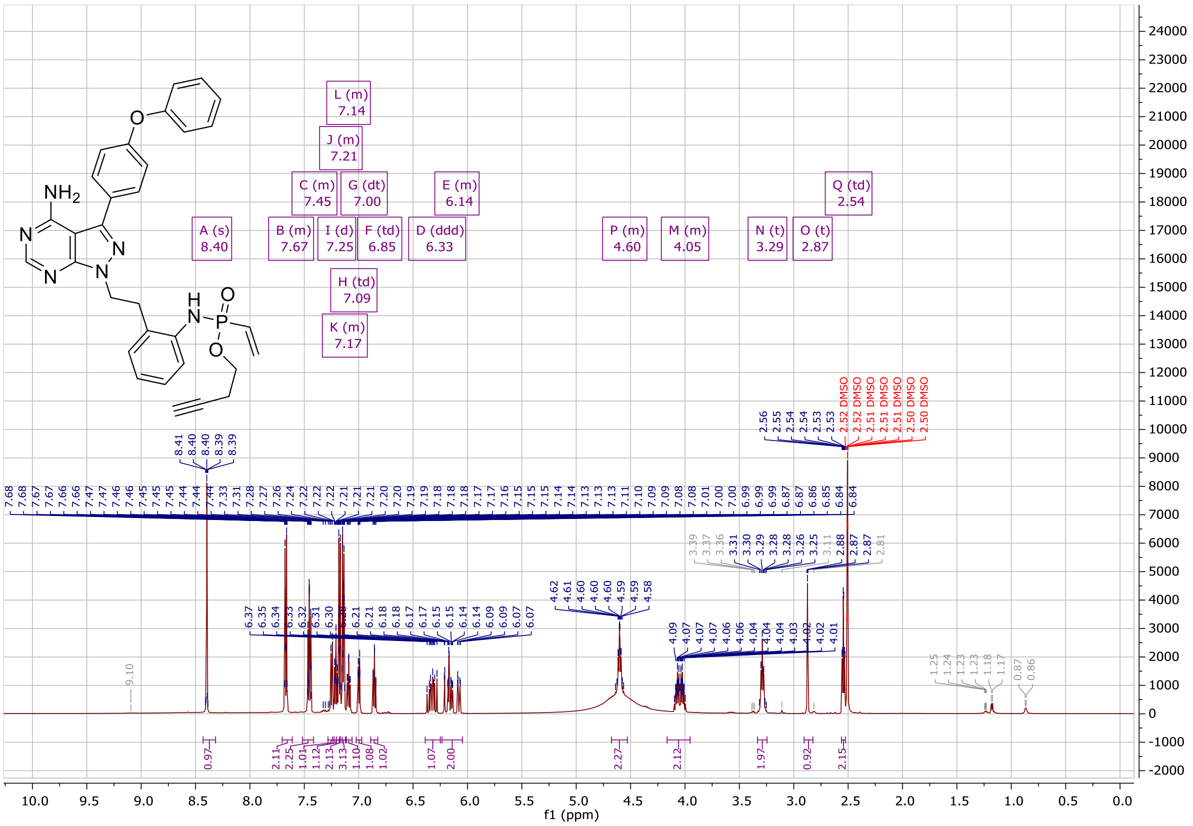

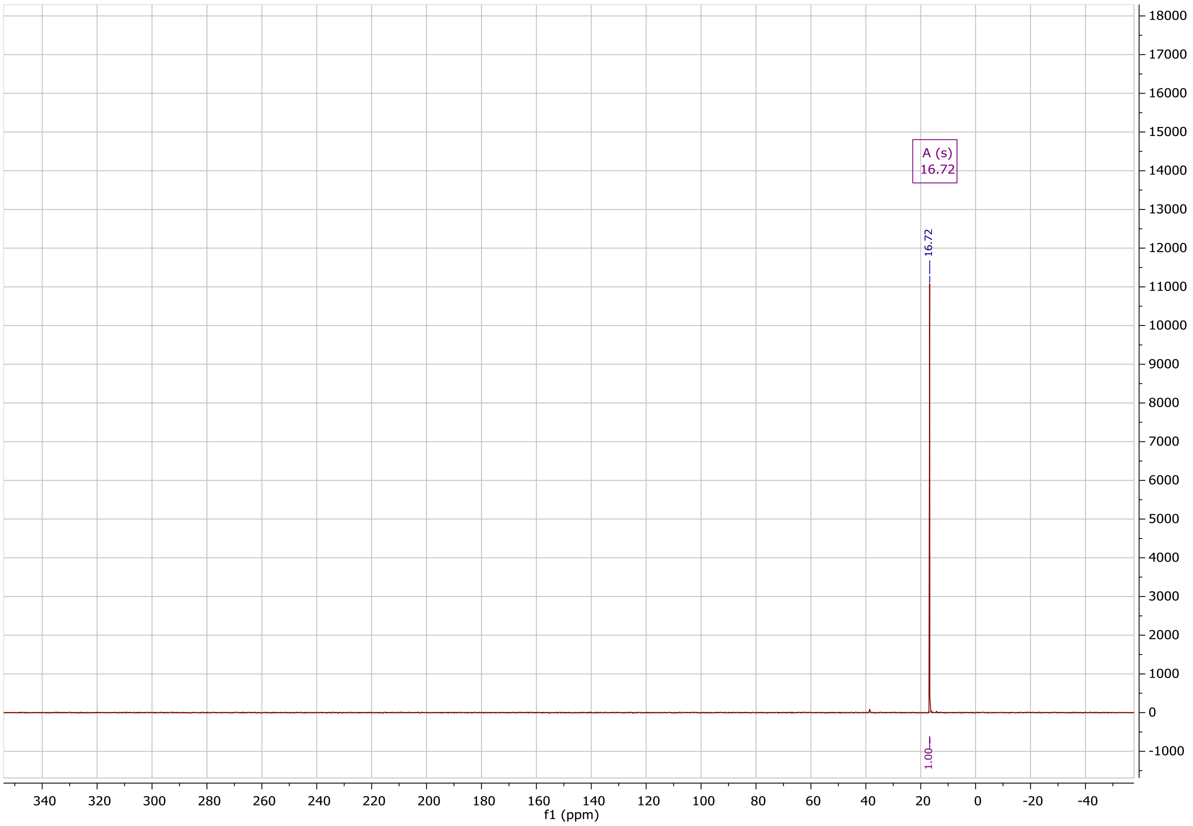

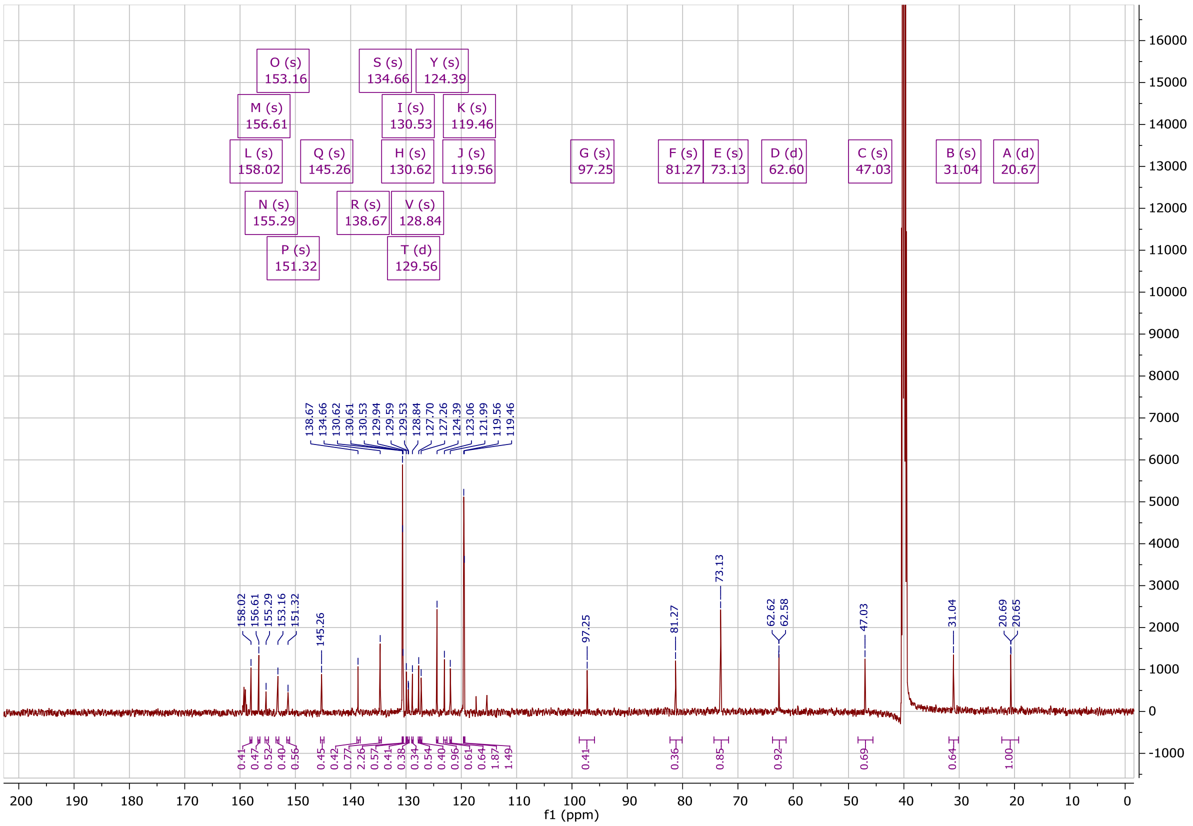

O (s) 153.16
S (s) 134.66
Y (s) 124.39
M (s) 156.61
I (s) 130.53
K (s) 119.46
L (s) 158.02
Q (s) 145.26
H (s) 130.62
J (s) 119.56
G (s) 97.25
F (s) 81.27
E (s) 73.13
D (d) 62.60
C (s) 47.03
B (s) 31.04
A (d) 20.67
N (s) 155.29
R (s) 138.67
V (s) 128.84
P (s) 151.32
T (d) 129.56
f1 (ppm)

but-3-yn-1-yl N-(2-((4-amino-3-(4-phenoxyphenyl)-1H-pyrazolo[3,4-d]pyrimidin-1-yl)methyl) phenyl)-P-vinylphosphonamidate (9):

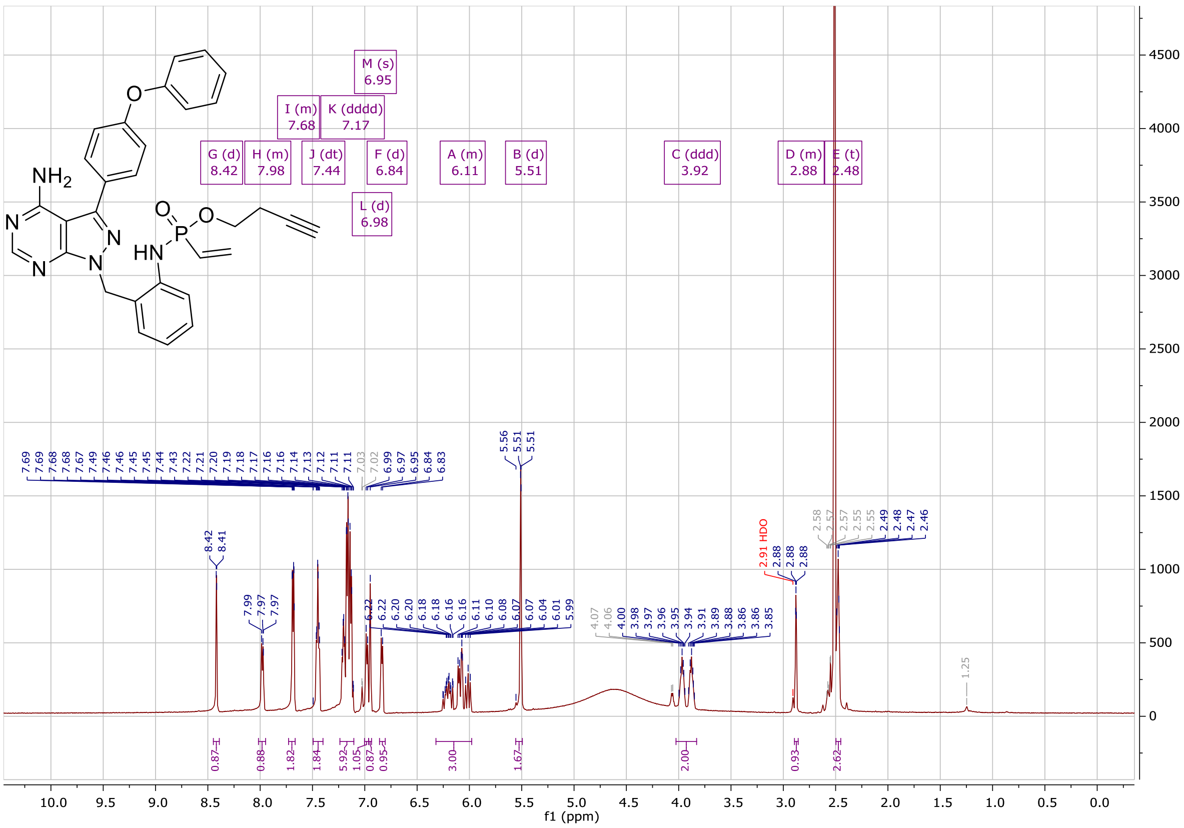

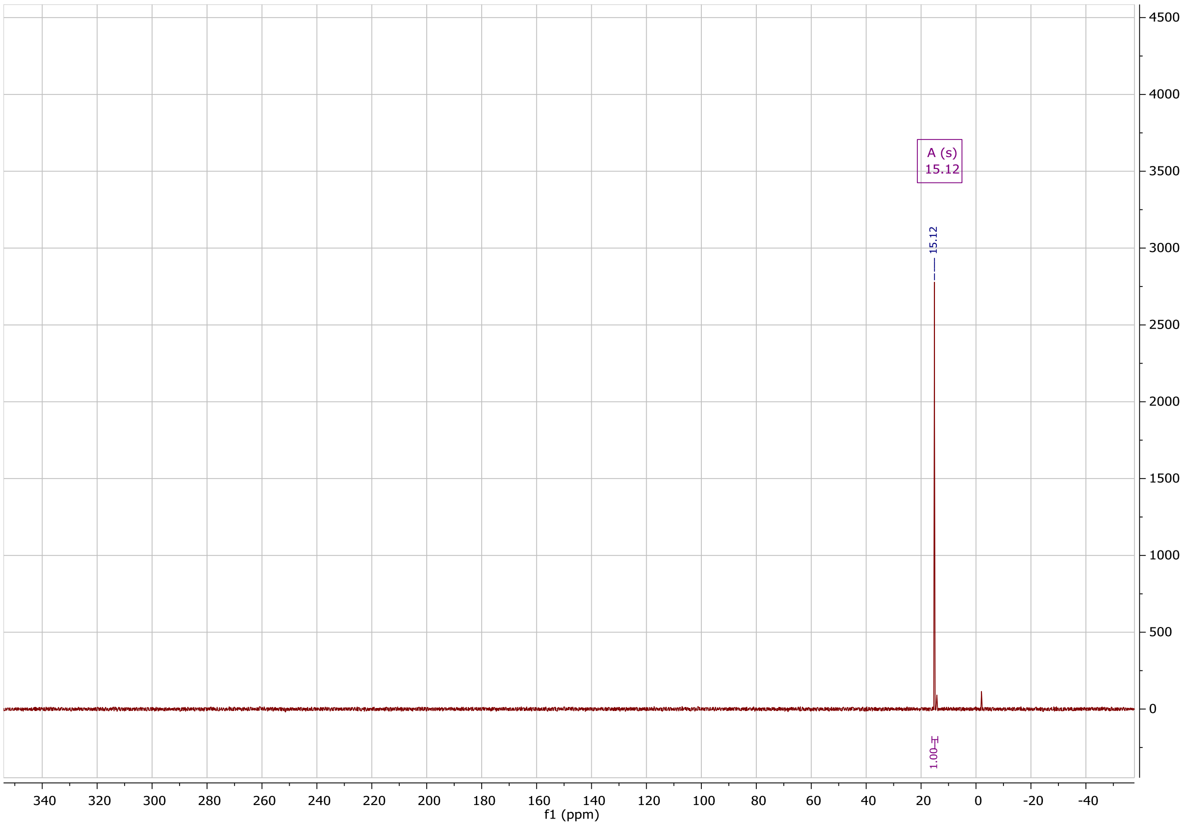

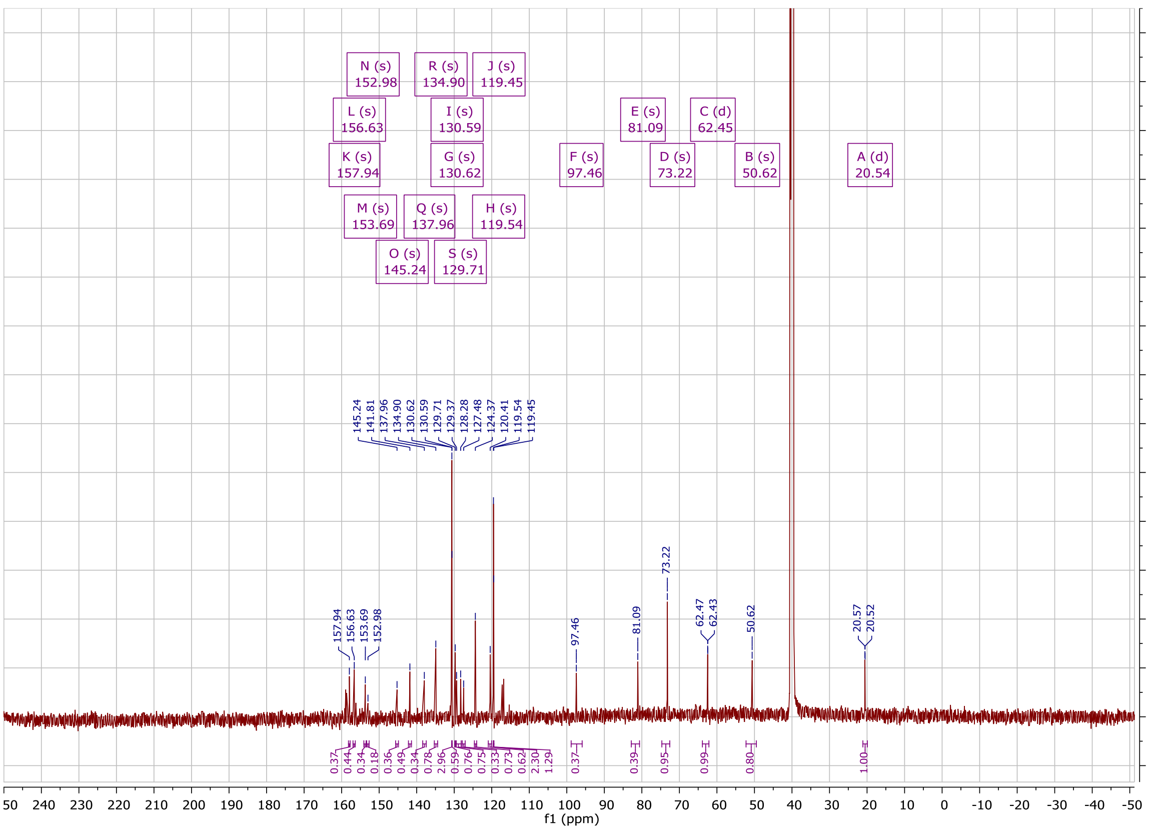

N (s) 152.98
R (s) 134.90
J (s) 119.45
L (s) 156.63
I (s) 130.59
E (s) 81.09
C (d) 62.45
K (s) 157.94
G (s) 130.62
F (s) 97.46
D (s) 73.22
B (s) 50.62
A (d) 20.54
M (s) 153.69
Q (s) 137.96
H (s) 119.54
O (s) 145.24
S (s) 129.71
f1 (ppm)

but-3-yn-1-yl N-(3-((4-amino-3-(4-phenoxyphenyl)-1H-pyrazolo[3,4-d]pyrimidin-1-yl)methyl) phenyl)-P-vinylphosphonamidate (10):

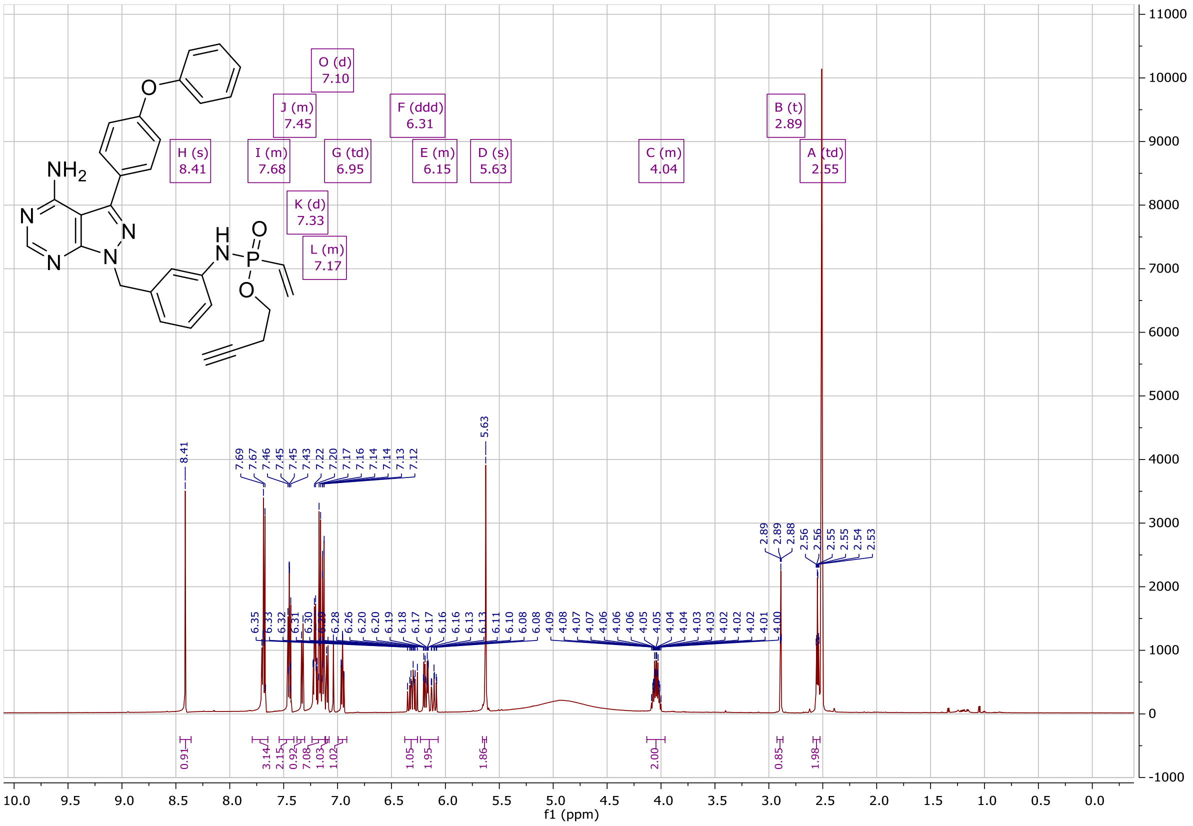

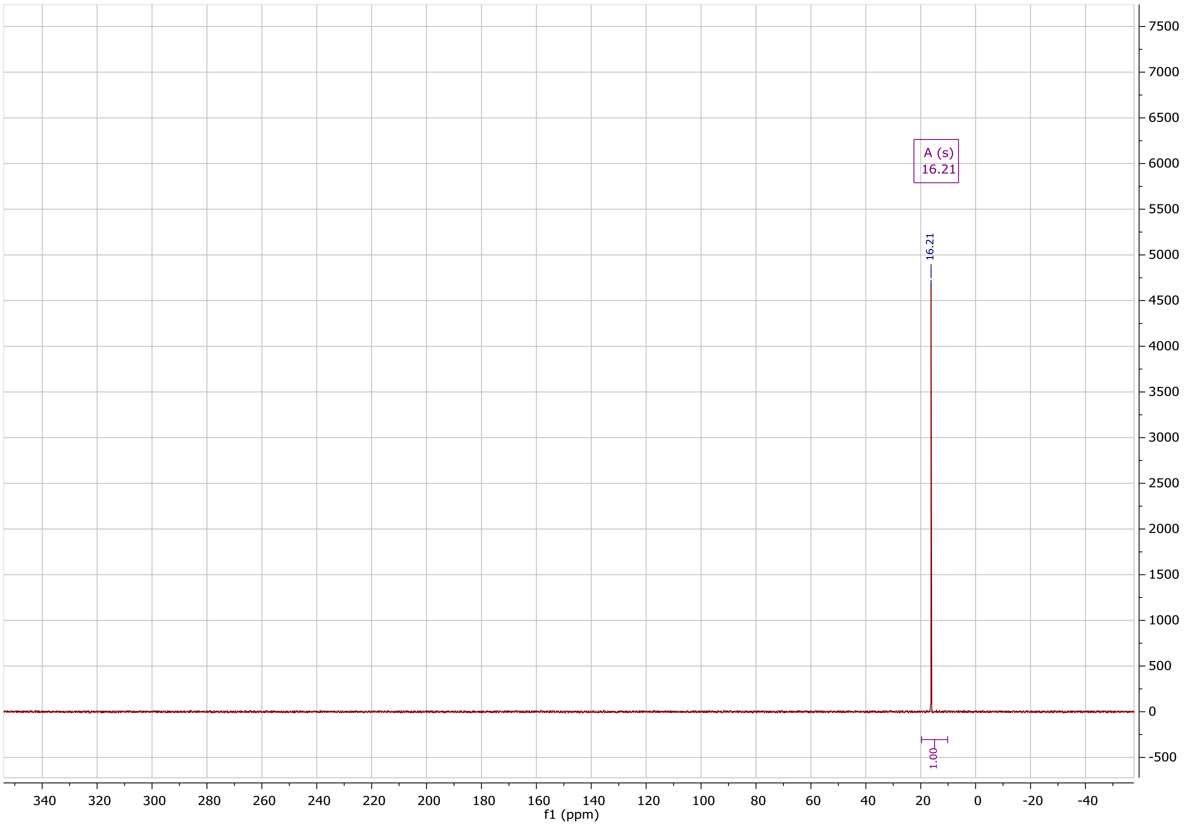

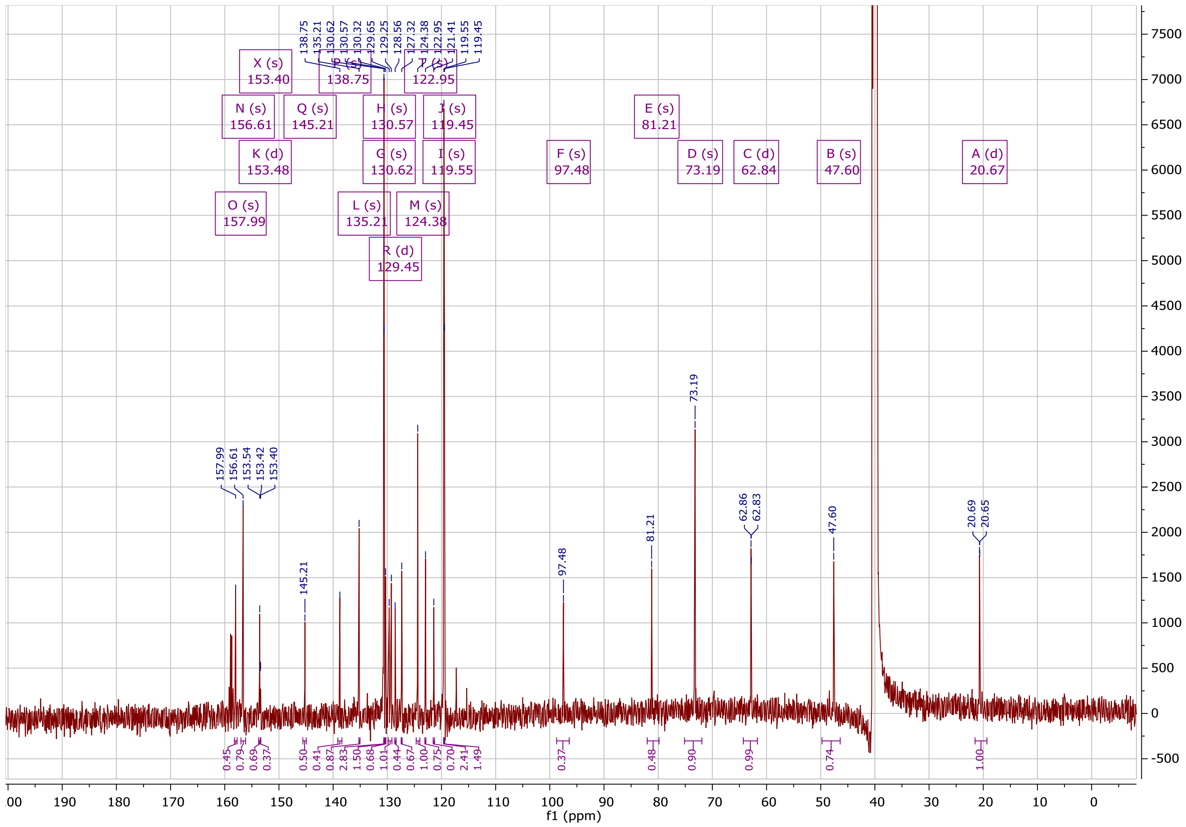

f1 (ppm)

but-3-yn-1-yl N-(4-((4-amino-3-(4-phenoxyphenyl)-1H-pyrazolo[3,4-d]pyrimidin-1-yl)methyl) phenyl)-P-vinylphosphonamidate (11):

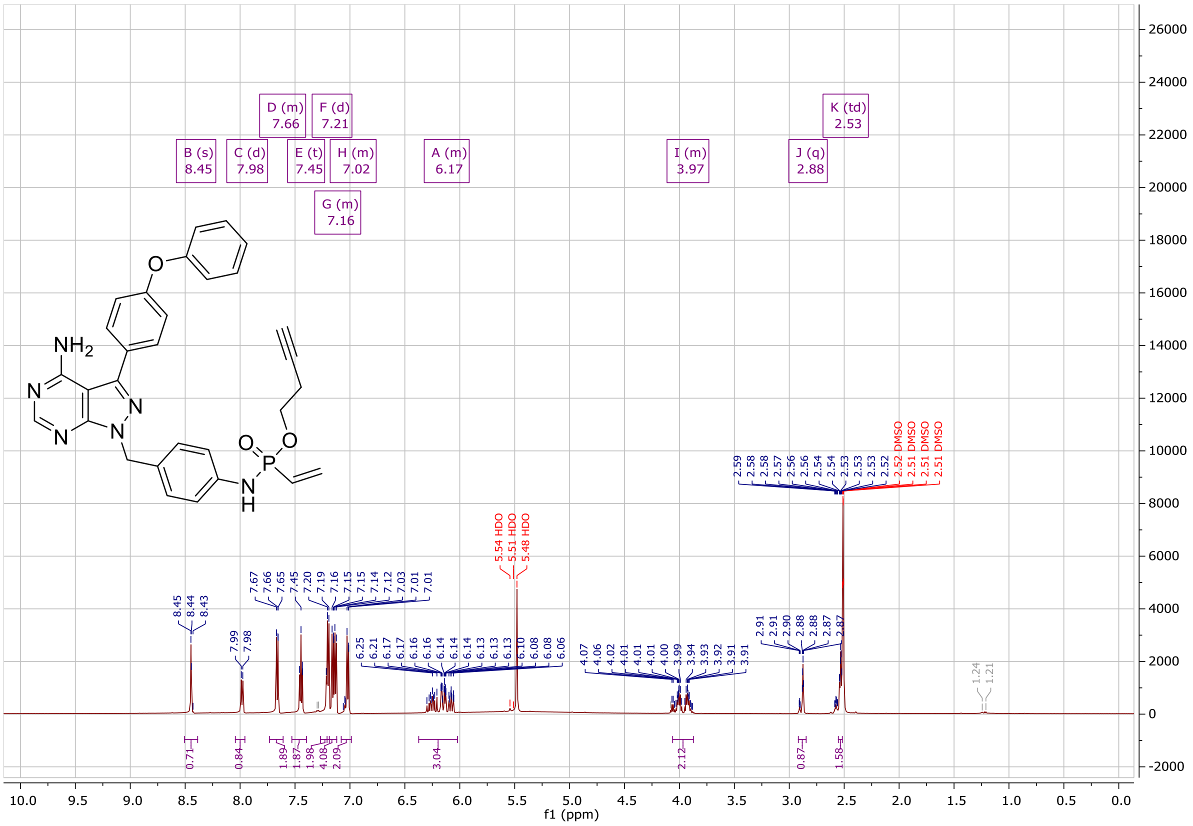

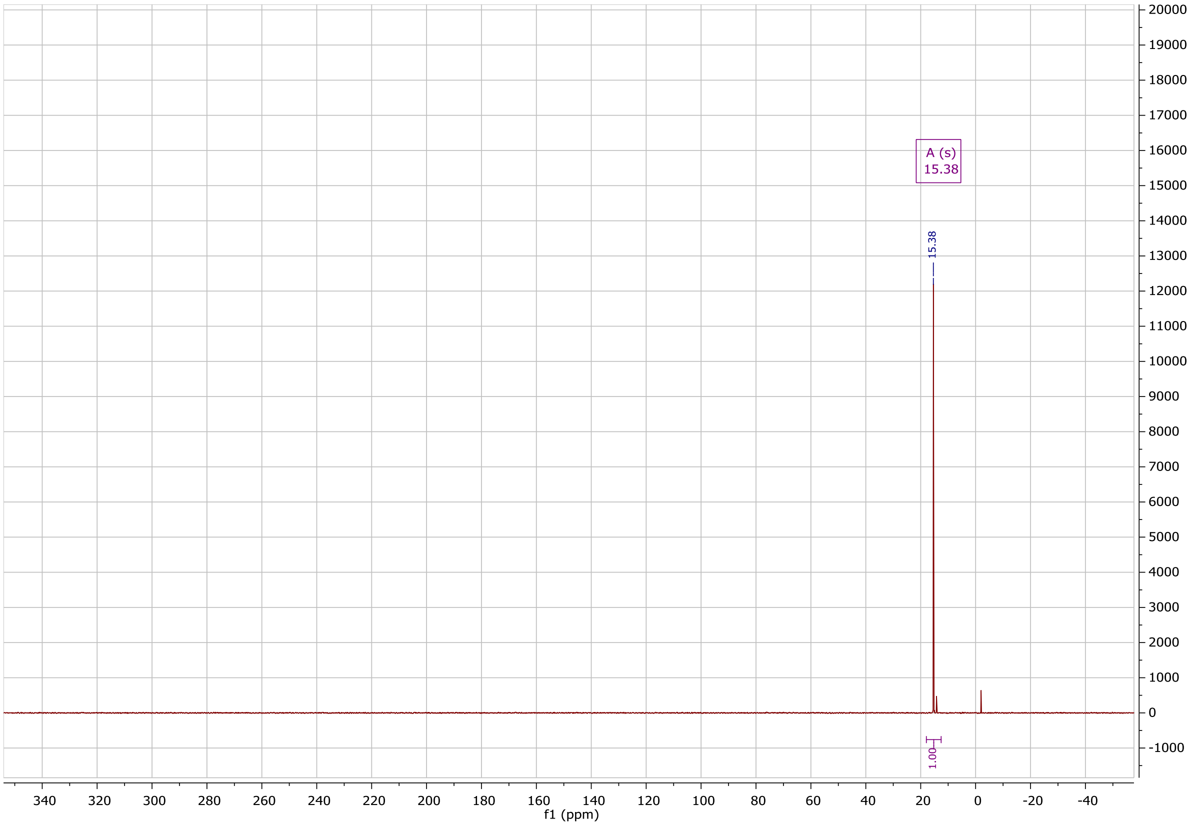

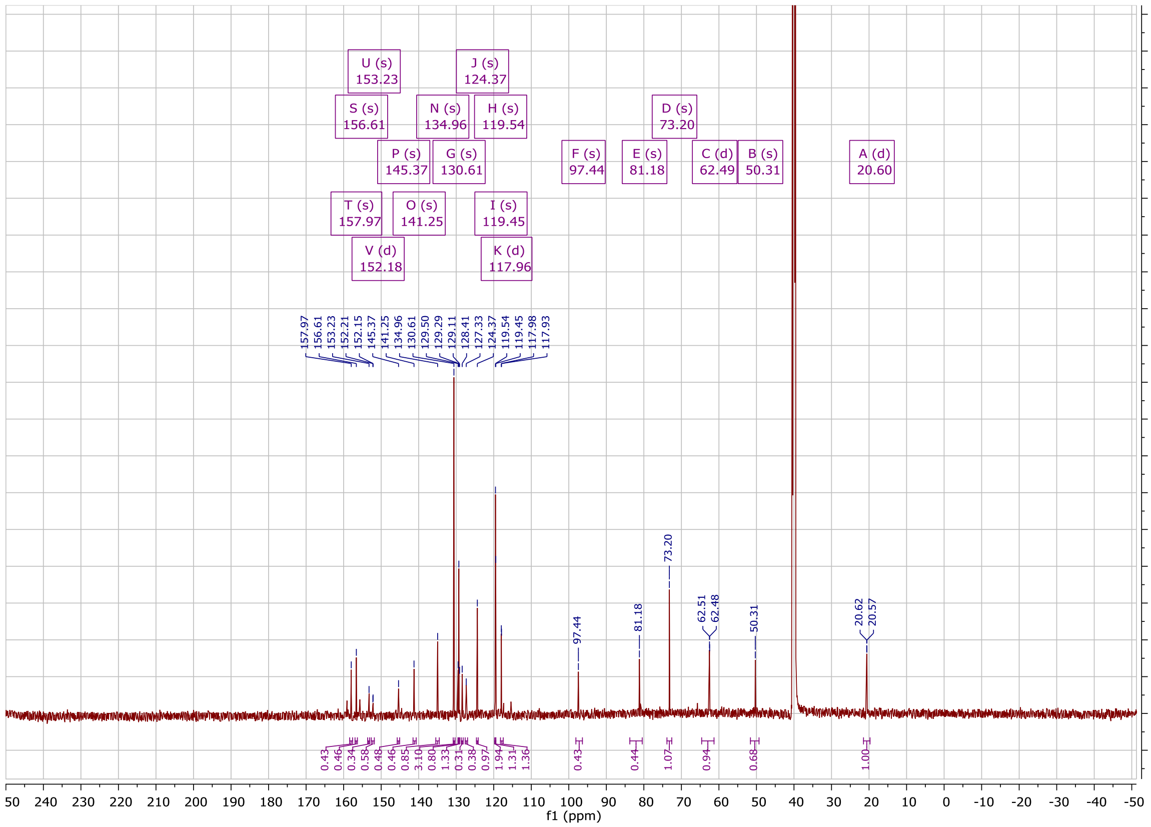

U (s) 153.23
J (s) 124.37
S (s) 156.61
N (s) 134.96
H (s) 119.54
D (s) 73.20
P (s) 145.37
G (s) 130.61
F (s) 97.44
E (s) 81.18
C (d) 62.49
B (s) 50.31
A (d) 20.60
T (s) 157.97
O (s) 141.25
I (s) 119.45
V (d) 152.18
K (d) 117.96
157.97 156.61 153.23 152.21 152.15 145.37 141.25 134.96 130.61 129.50 129.29 129.11 128.41 127.33 124.37 119.54 119.45 117.98 117.93
97.44
81.18
73.20
62.51 62.48
50.31
20.62 20.57
0.43 0.46 0.34 0.58 0.48 0.46 0.85 3.10 0.80 1.33 0.31 0.38 0.97 1.94 1.31 1.36 0.43 0.44 1.07 0.94 0.68 1.00
f1 (ppm)

ethyl N-(2-((4-amino-3-(4-phenoxyphenyl)-1H-pyrazolo[3,4-d]pyrimidin-1-yl)methyl)phenyl)-P-vinylphosphonamidate (9a):

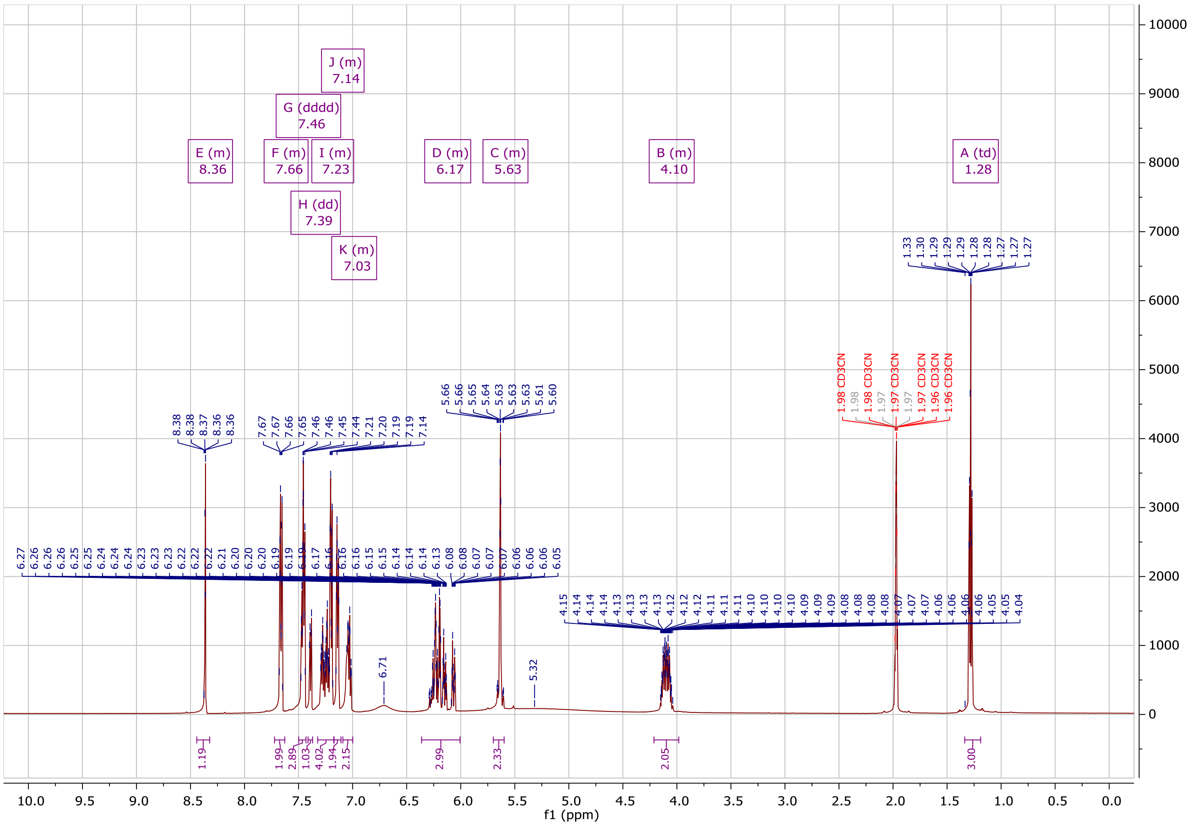

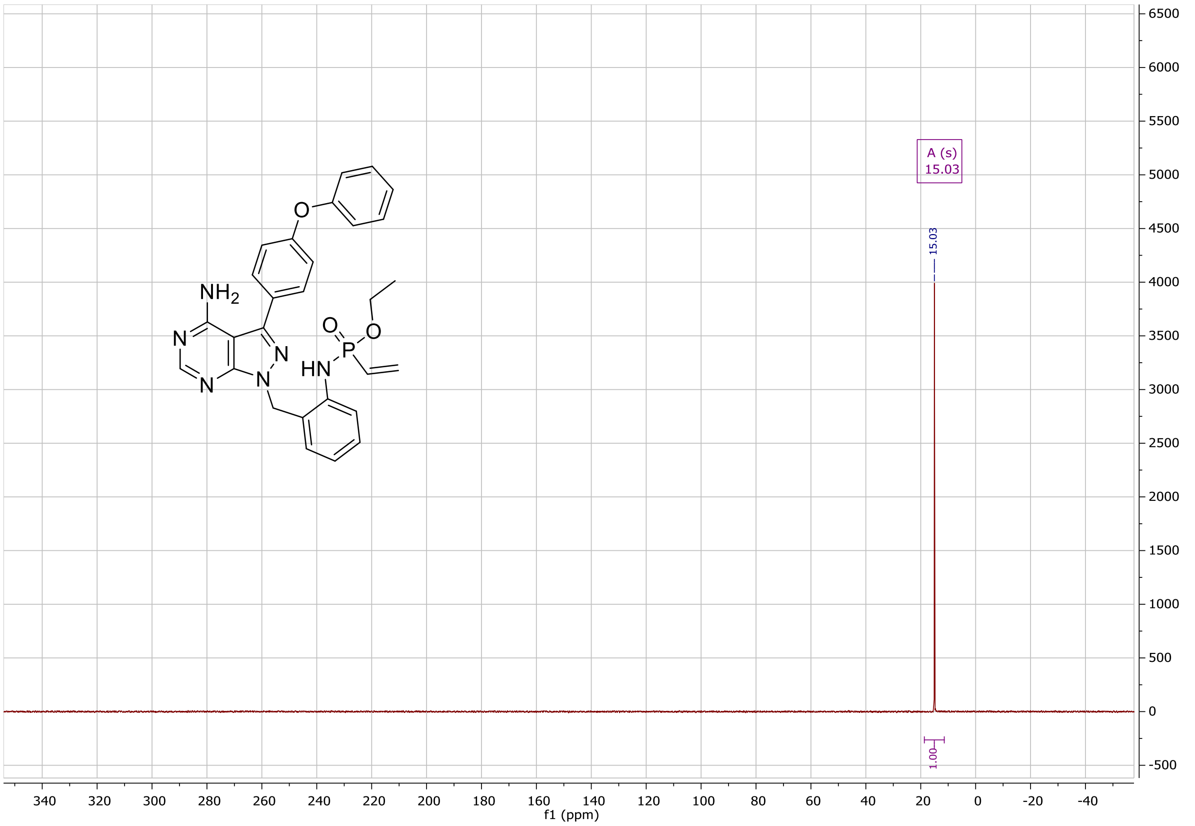

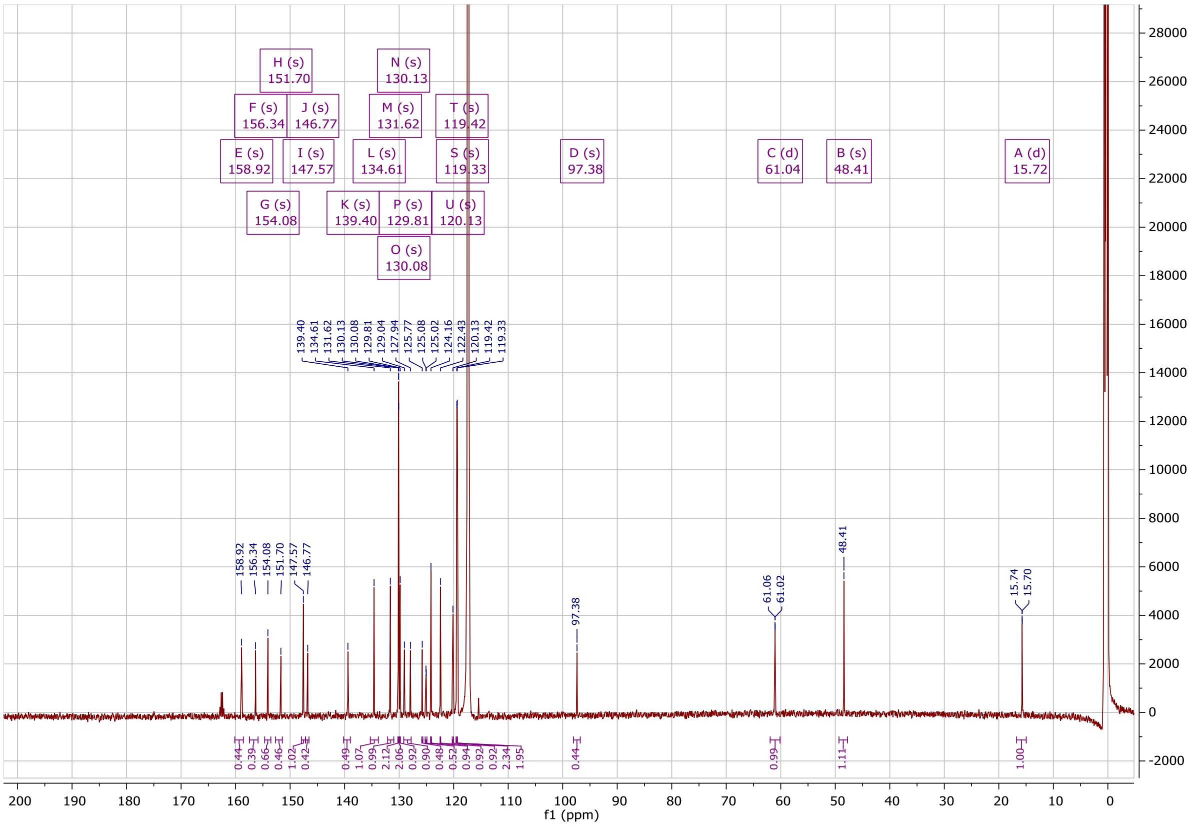

H (s) 151.70
N (s) 130.13
F (s) 156.34
J (s) 146.77
M (s) 131.62
T (s) 119.42
E (s) 158.92
I (s) 147.57
L (s) 134.61
S (s) 119.33
D (s) 97.38
C (d) 61.04
B (s) 48.41
A (d) 15.72
G (s) 154.08
K (s) 139.40
P (s) 129.81
U (s) 120.13
O (s) 130.08
f1 (ppm)

ethyl N-(2-((4-amino-3-(4-phenoxyphenyl)-1H-pyrazolo[3,4-d]pyrimidin-1-yl)methyl)phenyl)-P-ethylphosphonamidate (9b):

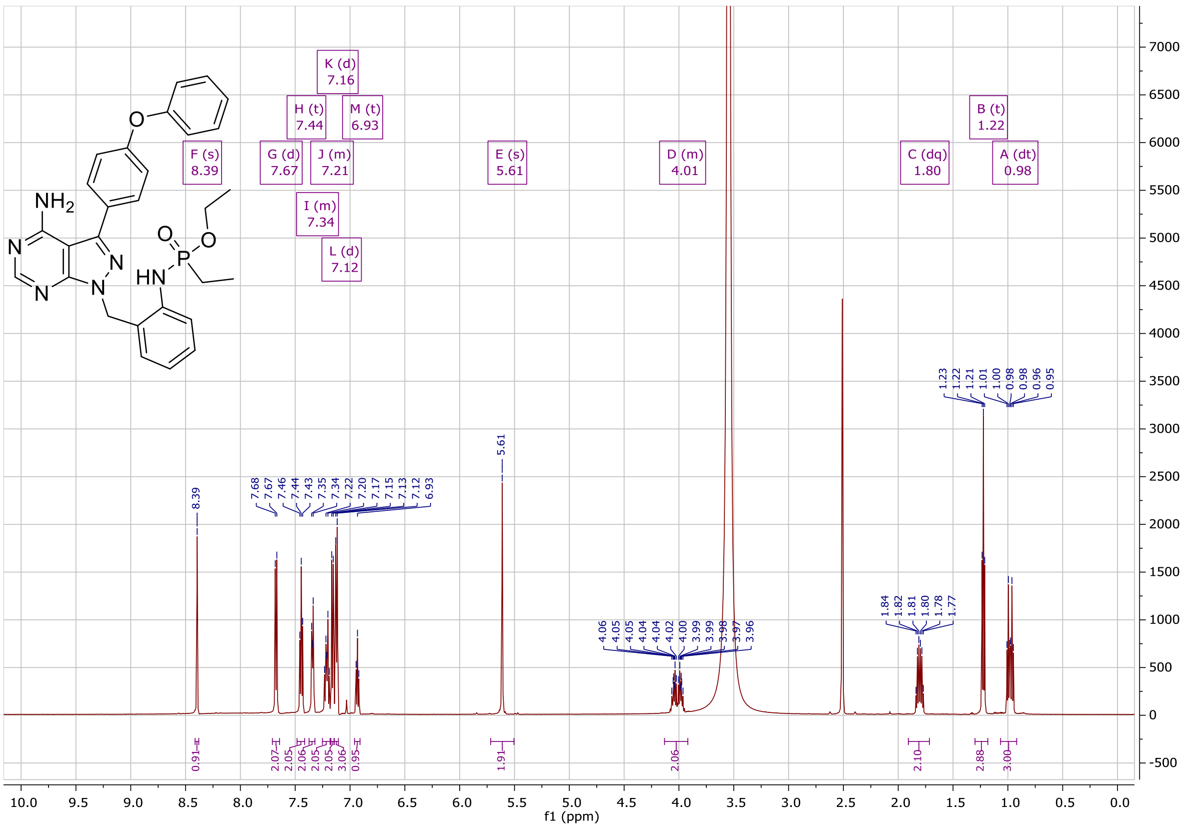

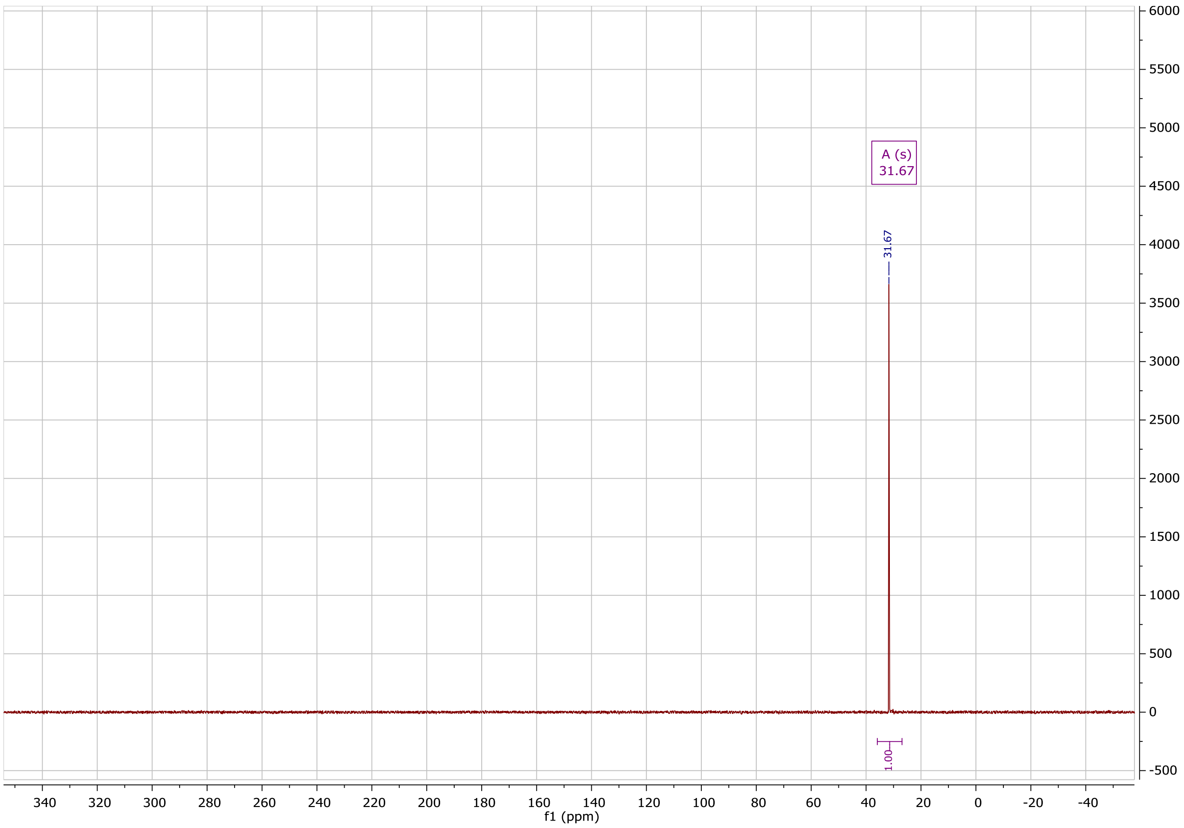

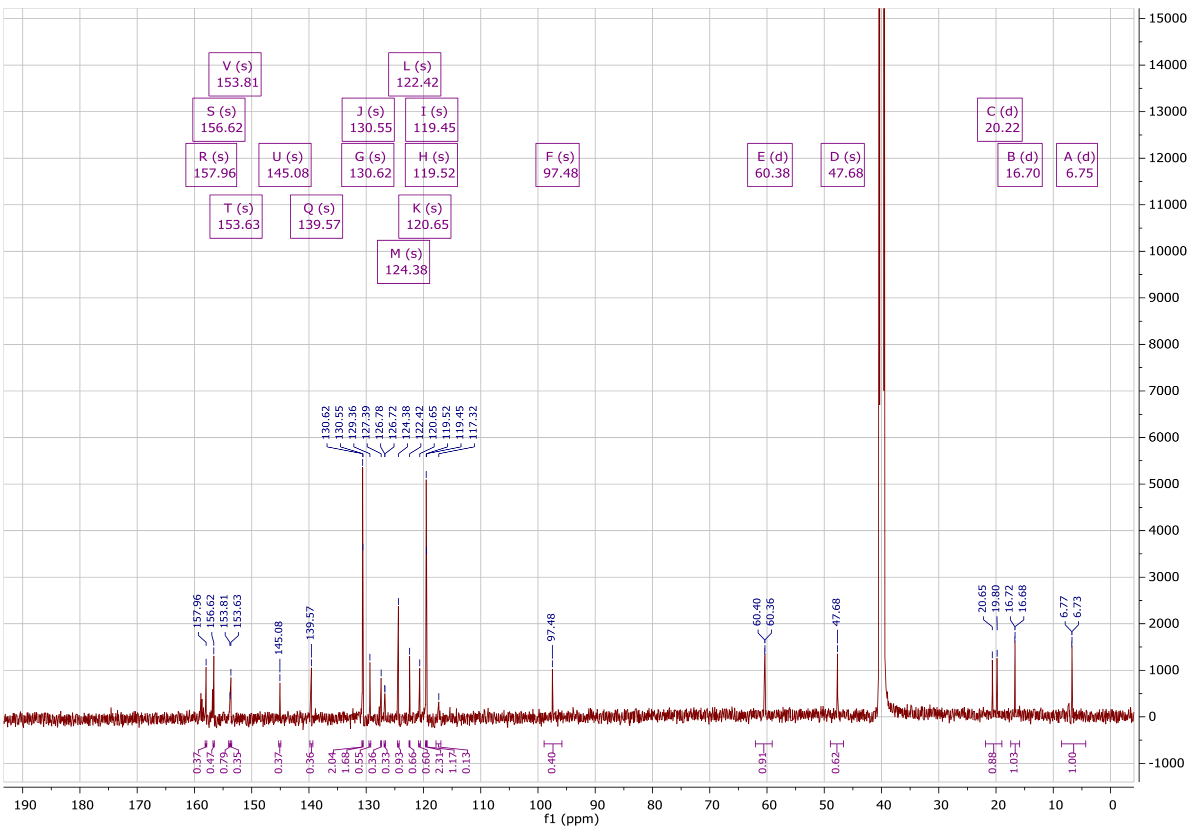
V (s) 153.81
L (s) 122.42
S (s) 156.62
J (s) 130.55
I (s) 119.45
C (d) 20.22
R (s) 157.96
U (s) 145.08
G (s) 130.62
H (s) 119.52
F (s) 97.48
E (d) 60.38
D (s) 47.68
B (d) 16.70
A (d) 6.75
T (s) 153.63
Q (s) 139.57
K (s) 120.65
M (s) 124.38
f1 (ppm)

Uncropped Gels & Blots:

<u>Figure 2d/S1</u>:

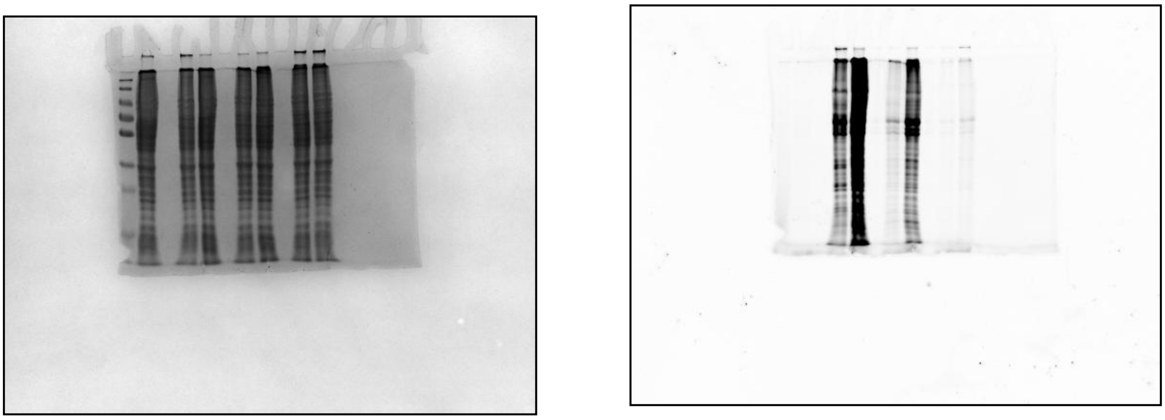

<u>Figure S2</u>:

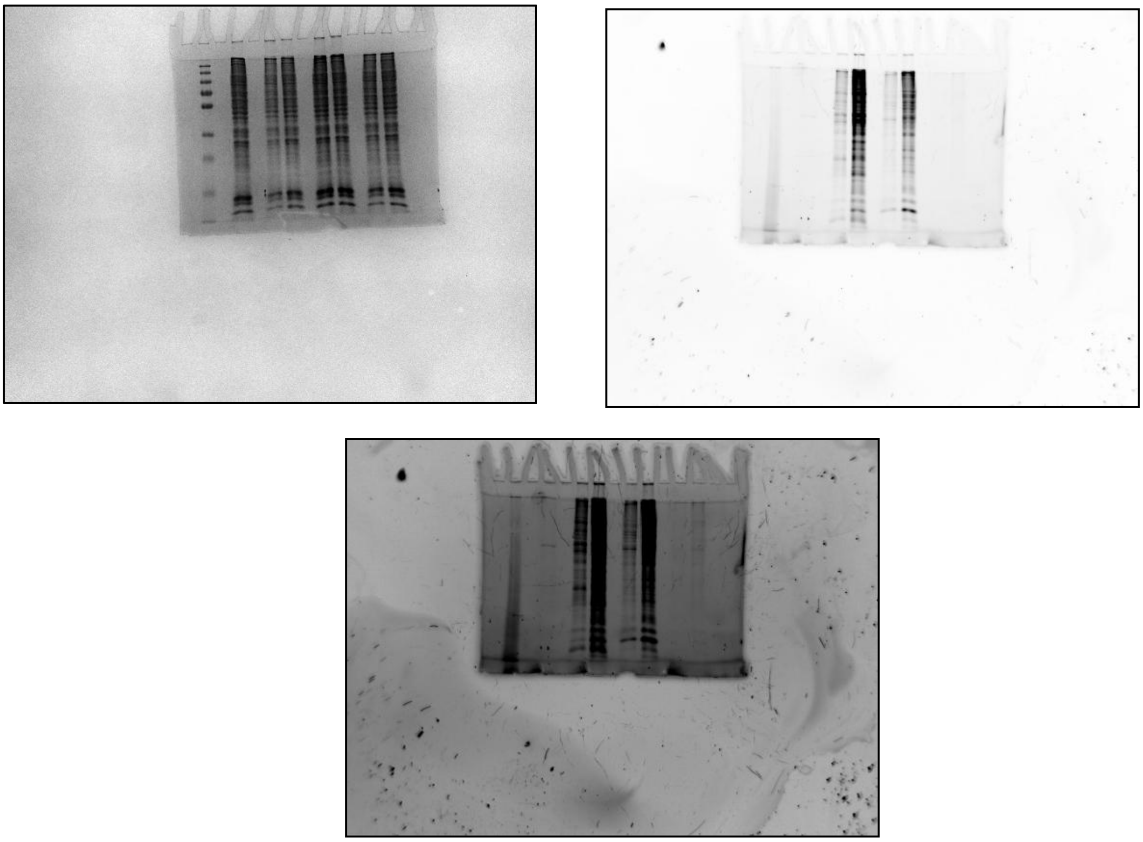

Figure 3d:

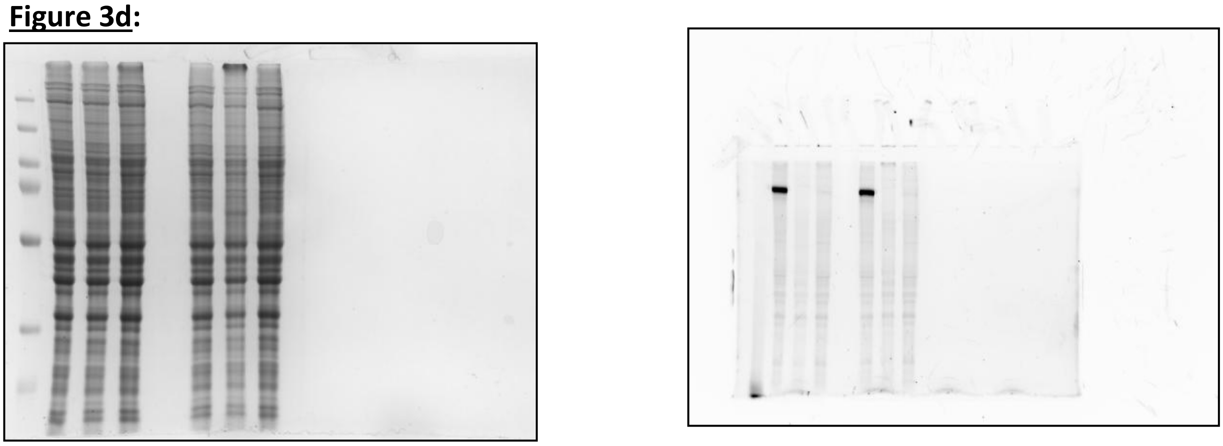

Figure S4:

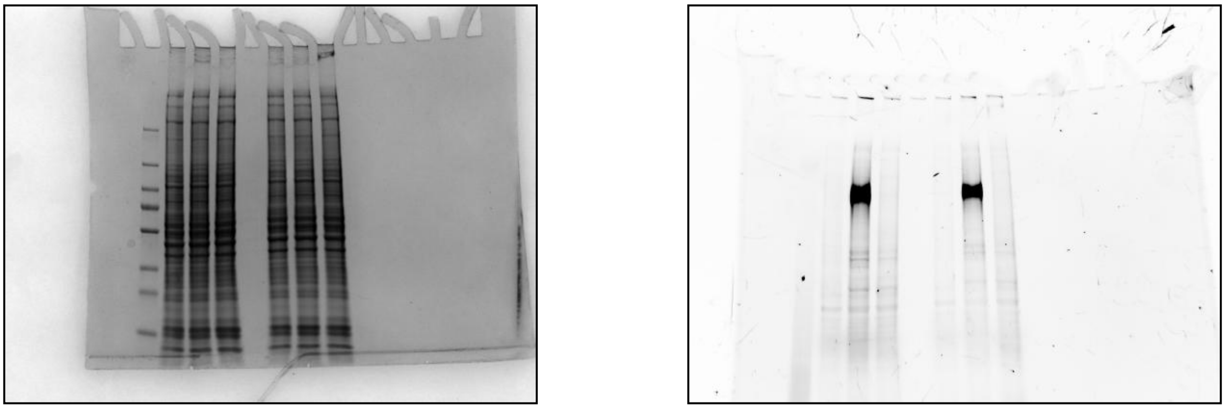

Figure S5:

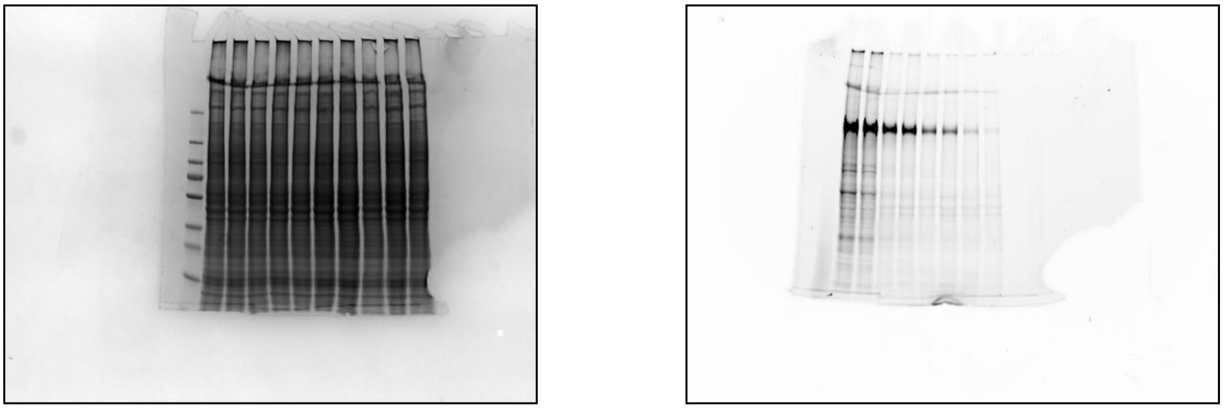

Figure 3e:

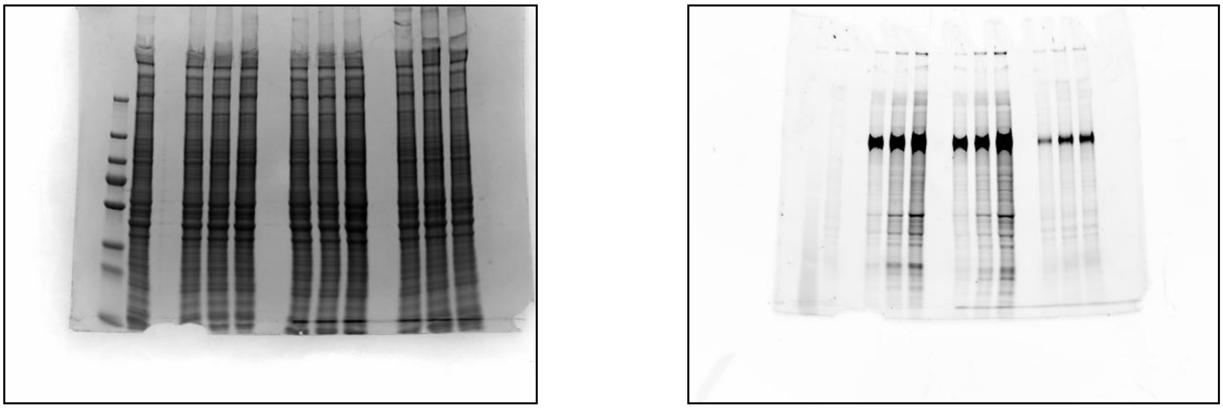

Figure 3f:

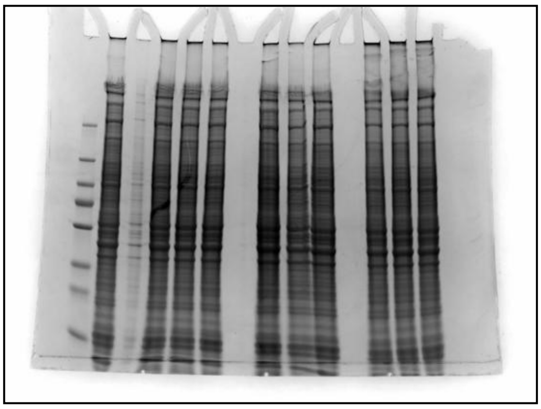

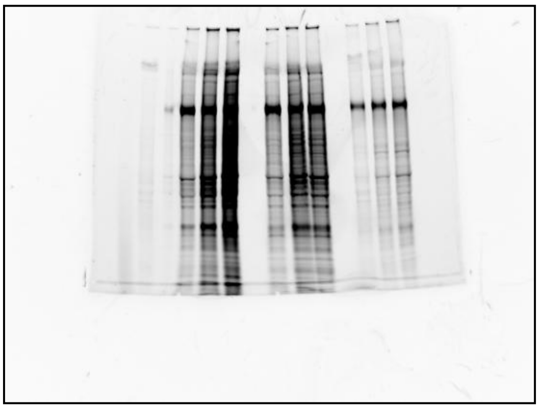

Figure S6:

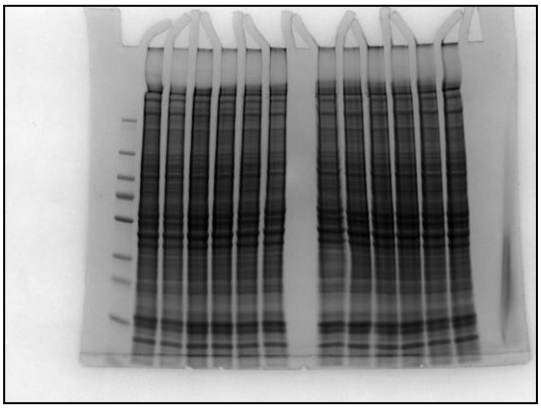

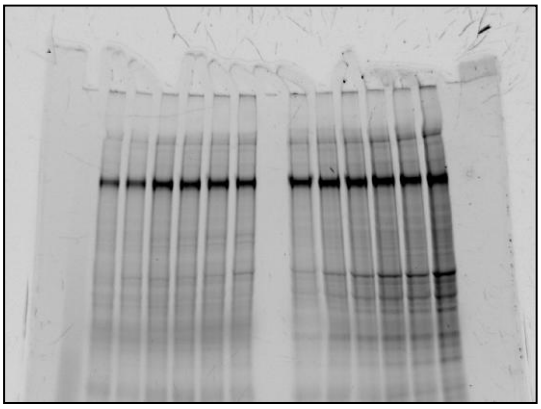

Figure S7a:

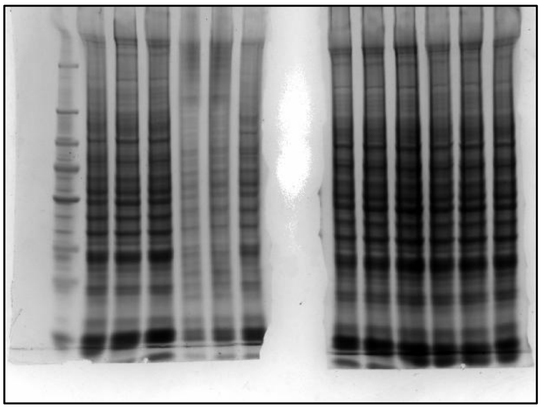

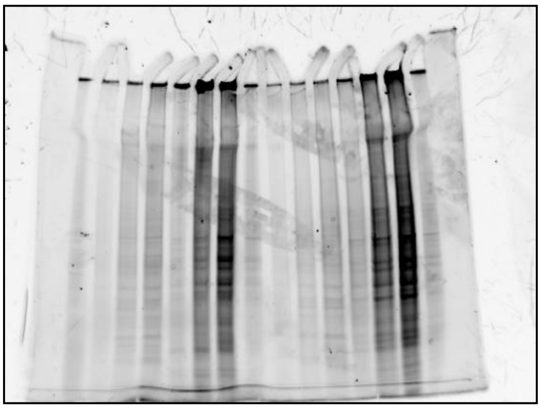

Figure S7a:

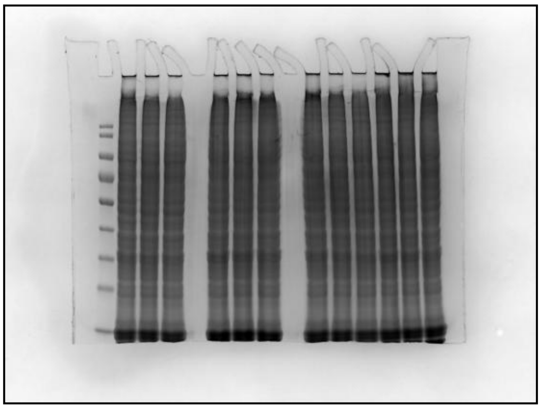

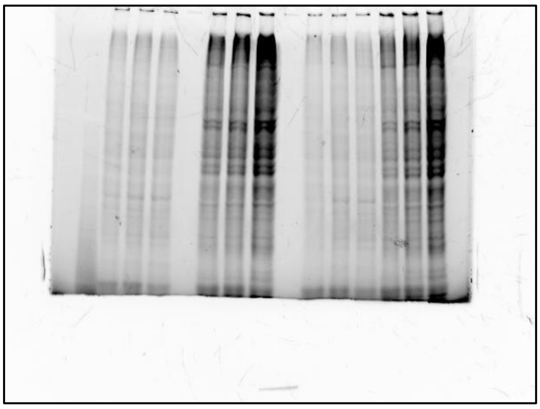

Figure 4b:

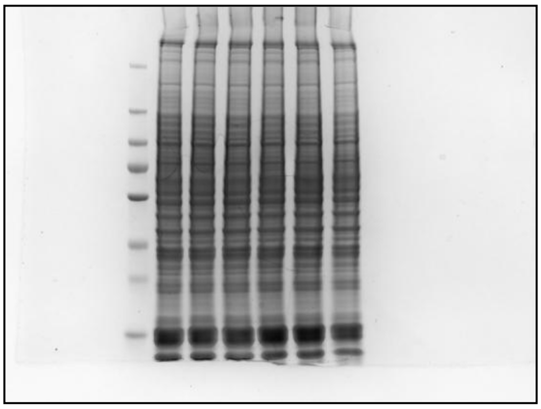

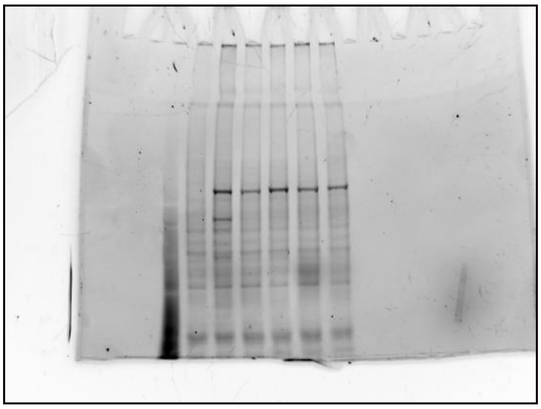

Figure S11:

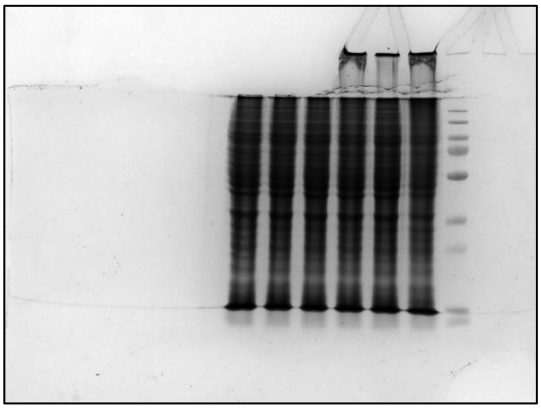

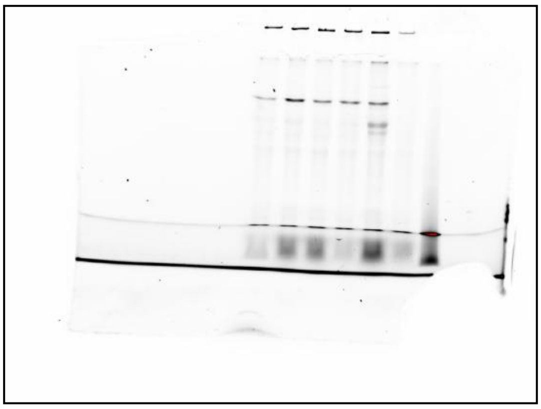

Figure 5b/S13:

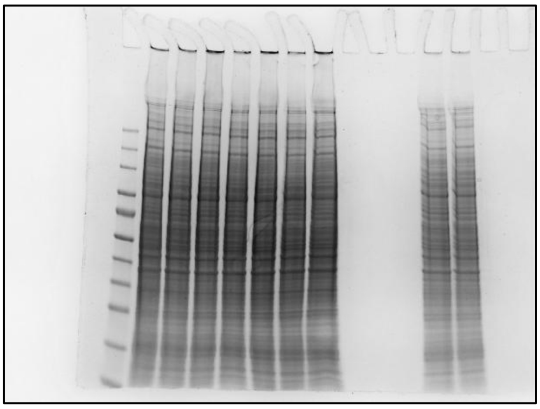

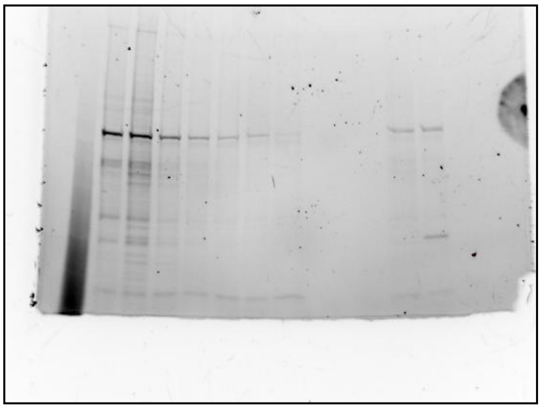

Figure 5c:

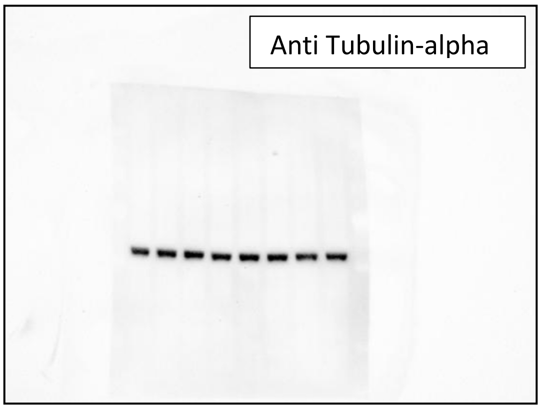

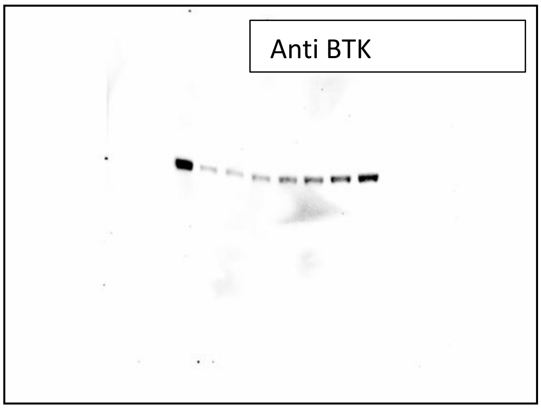

Supplement: Supplementary file 2 [file ja5c22349_si_002.pdf]
